# CD44-SNA1 integrated cytopathology for delineation of high grade dysplastic and neoplastic oral lesions

**DOI:** 10.1371/journal.pone.0291972

**Published:** 2023-09-25

**Authors:** Sumsum P. Sunny, Ravindra D. R., Aditi Hariharan, Nirza Mukhia, Shubha Gurudath, Keerthi G., Subhashini Raghavan, Trupti Kolur, Vivek Shetty, Vidya Bushan R., Avadhesha Surolia, Satyajit T., Pavithra Chandrashekhar, Nisheena R., Hardik J. Pandya, Vijay Pillai, Praveen Birur N., Moni A. Kuriakose, Amritha Suresh

**Affiliations:** 1 Department of Head and Neck Oncology, Mazumdar Shaw Medical Center, Bangalore, India; 2 Integrated Head and Neck Oncology Program (DSRG-5), Mazumdar Shaw Medical Foundation, Bangalore, India; 3 Manipal Academy of Higher Education, Manipal, Karnataka, India; 4 Department of Oral Medicine and Radiology, KLE Society’s Institute of Dental Sciences, Bangalore, India; 5 Department of Molecular Biophysics, Indian Institute of Science, Bangalore, India; 6 Department of Oral and Maxillofacial Pathology, KLE Society’s Institute of Dental Sciences, Bangalore, India; 7 Department of Electronic Systems Engineering, Division of EECS, Indian Institute of Science, Bangalore, India; University of Bari "Aldo Moro", ITALY

## Abstract

The high prevalence of oral potentially-malignant disorders exhibits diverse severity and risk of malignant transformation, which mandates a Point-of-Care diagnostic tool. Low patient compliance for biopsies underscores the need for minimally-invasive diagnosis. Oral cytology, an apt method, is not clinically applicable due to a lack of definitive diagnostic criteria and subjective interpretation. The primary objective of this study was to identify and evaluate the efficacy of biomarkers for cytology-based delineation of high-risk oral lesions. A comprehensive systematic review and meta-analysis of biomarkers recognized a panel of markers (n: 10) delineating dysplastic oral lesions. In this observational cross sectional study, immunohistochemical validation (n: 131) identified a four-marker panel, CD44, Cyclin D1, SNA-1, and MAA, with the best sensitivity (>75%; AUC>0.75) in delineating benign, hyperplasia, and mild-dysplasia (Low Risk Lesions; LRL) from moderate-severe dysplasia (High Grade Dysplasia: HGD) along with cancer. Independent validation by cytology (n: 133) showed that expression of SNA-1 and CD44 significantly delineate HGD and cancer with high sensitivity (>83%). Multiplex validation in another cohort (n: 138), integrated with a machine learning model incorporating clinical parameters, further improved the sensitivity and specificity (>88%). Additionally, image automation with SNA-1 profiled data set also provided a high sensitivity (sensitivity: 86%). In the present study, cytology with a two-marker panel, detecting aberrant glycosylation and a glycoprotein, provided efficient risk stratification of oral lesions. Our study indicated that use of a two-biomarker panel (CD44/SNA-1) integrated with clinical parameters or SNA-1 with automated image analysis (Sensitivity >85%) or multiplexed two-marker panel analysis (Sensitivity: >90%) provided efficient risk stratification of oral lesions, indicating the significance of biomarker-integrated cytopathology in the development of a Point-of-care assay.

## Introduction

Large scale screening and surveillance can reduce the burden of oral cancer, a major public health concern with approximately 400,000 new cases every year [1] and an incidence of 20 per 100,000 in the Indian subcontinent [2]. However, despite easy accessibility, around 60–80% of oral cancer patients are diagnosed at an advanced stage [2]. Screening programs that aim at detecting Oral Potentially Malignant Disorders (OPMDs) or early-stage cancers have demonstrated a definite reduction in mortality. Oral potentially malignant disorders (OPMDs) are defined as “any oral mucosal abnormality that is associated with a statistically increased risk of developing oral cancer” [3]. Early-stage cancer encompasses cancer that is classified within stage I and stage II. Biopsy, the current standard of diagnosis, is invasive with poor compliance and is unsuitable as a screening tool in large populations. Multiple non-invasive diagnostic adjuncts are currently available for early detection, however, pathology-based diagnosis is mandatory to delineate high-risk patients [4–7]. There is, hence, an urgent and unmet need for a minimally invasive, pathology-equivalent, point-of-care (PoC) oral cancer screening tool.

Cytology, a minimally-invasive method is proven to significantly reduce the incidence and down-stage cervical cancers [8]. In contrast, oral cytology lacks definitive diagnostic criteria and multiple studies including ours indicated a low sensitivity in the diagnosis of oral dysplastic lesions [9–11]. Biomarkers representing the carcinogenic processes of cell cycle regulation, signalling, and aberrant glycosylation can be invaluable adjuncts to improve the diagnostic accuracy of oral cytology. Mini-chromosome maintenance proteins (McM2), Laminin γ2, EGFR, CD17 and Ki-67 have been explored as markers in oral cytology [12, 13]. Lectins, known for their carbohydrate-binding specificities [14, 15] have been reported to distinguish cancers of the oral cavity, breast, cervix, and Barrett’s oesophagus [16]. Initial studies identified Wheat Germ Agglutinin (WGA) as capable of distinguishing oral cancer and dysplasia *in-vivo* and *ex-vivo* (sensitivity/specificity>80%) [17–19]. The evidence points out to the significance of biomarkers in improving diagnostic accuracy, however, further studies are essential to assess and validate their clinical applicability in oral cytology.

The spectrum of oral mucosal lesions includes benign, OPMD to invasive cancer with their management varying from conservative to surgical excision. The treatment decision depends on accurate risk stratification of the OPMDs; identification of the markers that achieve this objective will establish their clinical significance. The central hypothesis of this study was that marker-based cytology will delineate oral High-Grade Dysplastic (HGD: Moderate–Severe dysplasia) lesions and cancer and can be automated. The primary objective was to identify and validate markers in cytology towards developing a minimally invasive, PoC cytology method for delineation of Low-Risk lesions (LRL: Benign/Hyperplasia/mild dysplasia) and HGD lesions along with oral cancer.

## Materials and methods

### Study design

**Fig 1:**
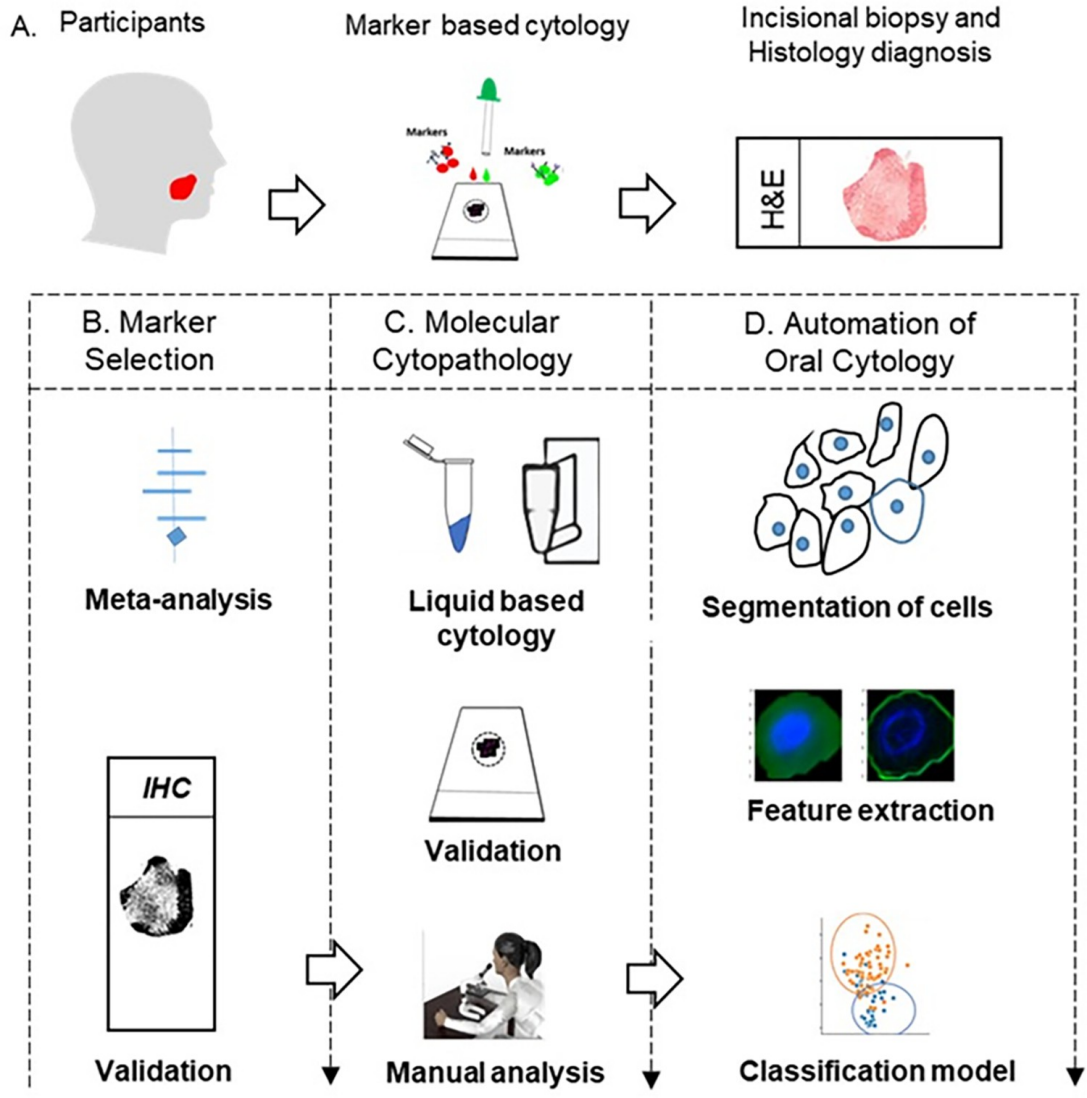
Study design. Participants were recruited according to inclusion and exclusion criteria, and individuals with oral lesions underwent brush biopsy (for marker-based cytology) followed by an incisional biopsy if indicated (A). Markers identified by systematic review and meta-analysis were validated in tissues (B). The liquid-based molecular cytology was performed with selected markers (C), single and multiplexed and marker expression was evaluated manually for the classification of oral cancer and HGD from Low-Risk lesions. The cytology image analysis was further automated by segmenting single cells, feature extraction of single cells and machine learning models developed (D). HGD: High Grade Dysplasia.

The study was implemented to identify and validate the best biomarker panel towards improving the diagnostic efficiency of oral cytology in delineating oral cancer/HGD from LRL. An evidence-based article search was conducted to find the markers used in delineating oral dysplastic from non-dysplastic lesion (Fig 1). The selected panel of markers were validated by IHC in histologically annotated tissues. The best markers selected were validated in cytology and automated the cytology image analysis using artificial intelligence.

### Systematic review and marker selection

A literature review of markers wherein immunohistochemistry (IHC) profiling delineates Dysplastic-OPMD (D-OPMD) from Non-Dysplastic-Oral lesions (ND-OL) was carried out. A search strategy combining terms ("oral premalignant lesions" OR "oral potentially-malignant disorders" OR "oral precancerous" OR "oral dysplasia" OR "oral cancer") and "immunohistochemistry" was used to identify relevant articles (1990 to 2017) in Pubmed (https://pubmed.ncbi.nlm.nih.gov/). The Preferred Reporting Items for Systematic Review and Meta-Analysis (PRISMA) was applied [20] (S1 Fig) and the articles were selected according to specific selection criteria (S1B Fig). The data extracted from the articles included number of true-positives, false-positives, true-negatives, false-negatives and IHC scores (S1 Table). The quality was assessed by Quality Assessment of Diagnostic Accuracy Studies (QUADAS-2) [21] and publication bias was established by funnel plots. The marker selection was based on three benchmarks i) minimum of three studies ii) identified by high-throughput analysis and iii) identify aberrant glycosylation patterns in solid tumors.

### Patient cohorts

The study was approved by Narayana Hrudayalaya Medical Ethics Committee (NHH/MEC-CL-2016-393), Narayana Hrudayalaya, Bangalore, India and, KLE Society’s Institute of Dental Science, Bangalore, India. This cross-sectional prospective investigation was carried out as per the guidelines of the Declaration of Helsinki, and its results were reported in accordance with the **Strengthening the Reporting of Observational studies in Epidemiology** guidelines [22]. The oral epithelial cells (brush biopsy) and tissues were collected from Head and Neck Oncology clinic, Mazumdar Shaw Medical Center, Narayana Hrudayalaya, Bangalore, India and Oral Medicine clinic, KLE, Bangalore, India, after written informed consent (December-2017 to March-2021). The subjects with oral lesion/s clinically suspected to be benign, OPMD, or oral cancer, greater than 18 years of age were included in the study. Subjects suffering from any acute illness or debilitating systemic diseases that preclude biopsy were excluded.

### Immunohistochemistry validation

Patients were categorized as LRL (benign, hyperplasia, mild-dysplasia), HGD (moderate-severe dysplasia) and carcinoma. The selected markers (S2 Table and S1 Appendix) were validated by IHC in formalin-fixed paraffin-embedded tissue sections (FFPE) in two phases using standard protocols [23]. Briefly, the sections were deparaffinised, incubated with the primary antibody (S2 Table) and detected using the secondary detection system (Dako-Real Envision, K5007). The necessary controls were included (positive/negative). The sections were assessed (400x; 5–10 images/slide; Nikon Eclipse E200) for their intensity (2, 4 or 6), percentage positivity and the final score calculated (percentage positivity x intensity). Staining in the nucleus, cytoplasm, and/or cell membranes indicated positive expression and scoring was blinded to the histology diagnosis.

### Immunocytology validation

#### Cell culture and maintenance

Cal-27 (originated from moderately differentiated squamous cell carcinoma of tongue; a gift from Institute of Bioinformatics, Bangalore) was cultured in DMEM (FBS 10% + antibiotics 1x- Penicillin-Streptomycin), while DOK (dysplastic cell line, obtained from RPCI, NY, USA) was cultured in DMEM (FBS 10%+5ug/ml hydrocortisone). The cell lines were expanded using standard protocols [23] and used for cytology experiments.

#### Liquid based cytology and immunocytology

The cells were collected from oral lesions and contralateral normal sites prior to biopsy (S2 Fig and S1 Appendix). In lesions indicated for biopsy (OPMD, cancer), histology diagnosis was considered as reference standard, while clinical diagnosis was considered in other subjects (benign lesions, normal mucosa). In order to establish the nomogram, cells were collected from buccal mucosa, tongue and gingiva of healthy subjects without any habits or oral lesions. A cervical cytology brush or Rover Orocellex brush (Rovers Medical Devices B.V, Netherlands) was used to harvest the cells (BD SurePath, BD Biosciences, USA). The cells were centrifuged using the Cytospin^TM^ 4 (Thermo-Scientific; Cat: A78300003, USA). The slides were incubated with the primary antibody (S2 Fig) and staining in the nucleus, cytoplasm, and/or cell membranes was considered positive expression. The slides were visualized (200x; Nikon Eclipse E200, Nikon, USA) and the intensity, pattern of staining and percentage positivity were assessed (10–15 fields/slide). For the fluorescent conjugated-markers, the cells were washed with phosphate buffered saline (PBS) and incubated for 15 minutes and counterstained with the nuclear stain DAPI. In multiplex cytology the cells were incubated with markers sequentially before being counterstained with DAPI. Images were captured (20x objective, Zeiss C, Axiocam; Zen lite 2012), individual cell pixel intensity measured (50–70 cells, Image J) and compared across the different assays/samples (S1 Appendix). The pixel intensity was measured using ImageJ (https://celldivisionlab.com/2015/08/12/using-imagej-to-measure-cell-fluorescenc/) [24]. The respective H&E slides (cytology) were interpreted by two oral pathologists blinded to histology diagnosis.

### Automated analysis of cytology image analysis

#### Single cell segmentation and extraction of quantitative features

**Fig 2:**
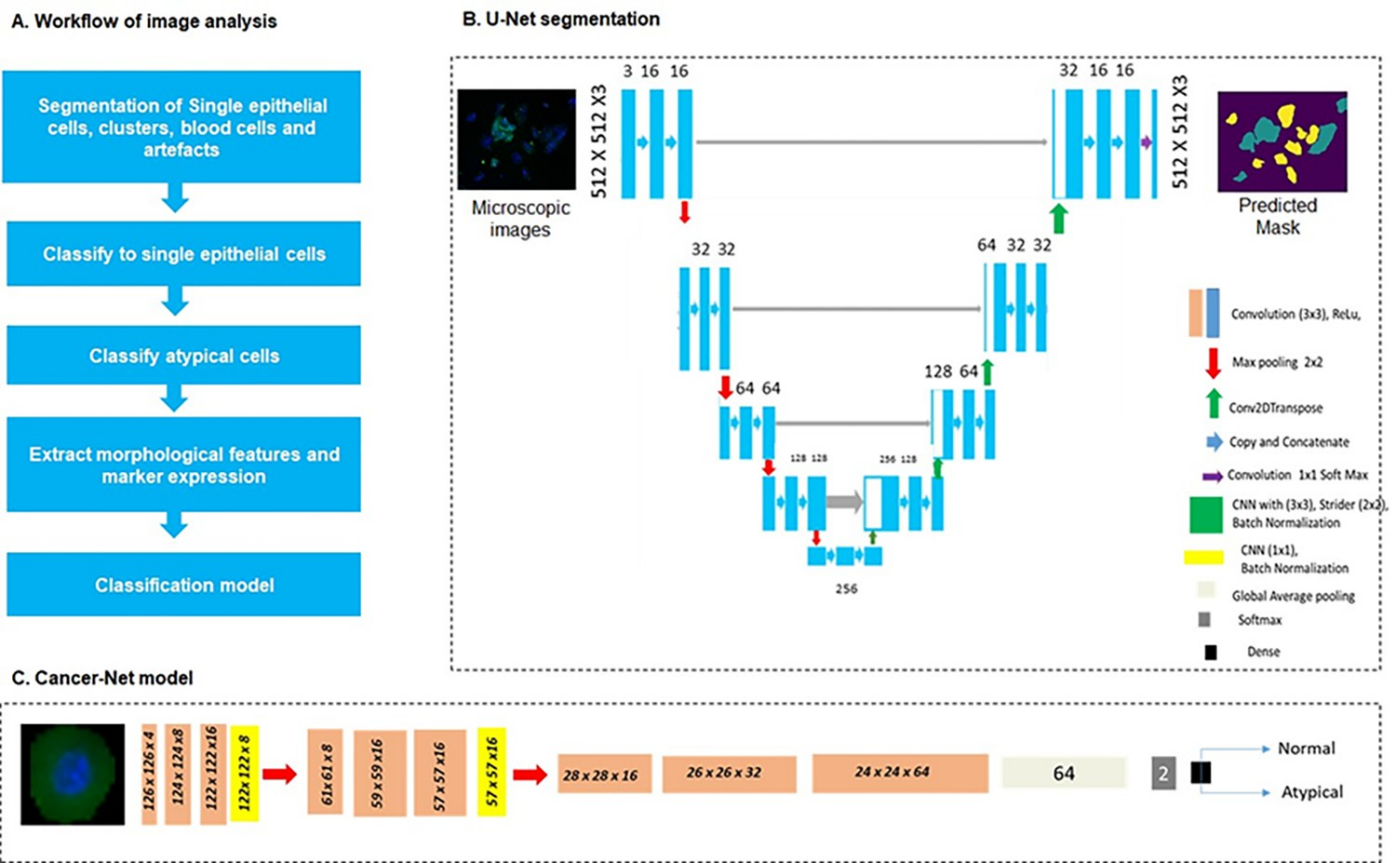
Workflow of image analysis and deep learning models. Fluorescent microscopic cytology images contain epithelial clusters, blood cells, and artefacts along with single epithelial cells (A). Single epithelial cells were segmented using the U-Net model (B) and classified as atypical and normal cells using the Cancer-Net model (C), and quantitative features were extracted for developing the classification model.

Single epithelial cells (Fig 2) were extracted from single marker stained fluorescent images (using U-Net segmentation model) and classified (Artefact-Net model) after pre-processing as explained previously [25]. Atypical cells [11] with increased nuclear-cytoplasmic ratio, irregular nuclear shape, and abnormal cell shapes were selected from segmented cytology images of the patient. In order to classify these cells, Inception-V3 and Cancer-Net, a Convolutional Neural Network (CNN) model with fewer parameters were built. Inception-V3 was trained using the last 164 layers and Cancer-Net was developed with 3x3 CNN filters with ReLu and Batch Normalization for feature extraction with 4, 8 and 16 filters before initial MaxPooling. A CNN with kernel size 1X1 was used before max-pooling. Three blocks of CNN (27 layers) were used before global average pooling as Fig 2C. Both CNN was trained for 150 epochs with batch size 16, loss function as binary cross-entropy, and Adam was used as an optimizer.

Single epithelial cells were smoothened by median filter and images augmented by random rotation, width shift, height shift, shear range, zoom range, horizontal flip, and vertical flip. The evaluation metrics used were accuracy, F1 Score, sensitivity, and specificity. The morphology features (nuclear-cytoplasmic area ratio, diameter ratio, perimeter ratio, major and minor axis ratio, solidity, orientation, eccentricity, and convex area) and marker expression of each cell and nucleus, were extracted from single cells using SkImage-region props library in python. The expression features such as mean intensity of markers and probability score of the atypical CNN model was also considered for feature vector generation. The feature of all cells of each patient was used to develop a statistical aggregate (average, maximum and standard deviation) and these statistical aggregates were used to develop multiple machine learning models.

### Statistical analysis

The systematic review and meta-analysis were performed using Review Manager 5.3 from Cochrane collaboration and MetaDisc Version 1.4. The odds ratio (OR; for dichotomous data) and mean difference (continuous data) were calculated (Forest Plot analysis) to find the association between the marker and dysplasia. Summary estimates were expressed in terms of OR/Summary Area under curve (sAUC). The heterogeneity between the studies was evaluated by Chi-square test (P<0.05) and I^2^ (>50%) with random effects model. A sensitivity analysis was performed if heterogeneity is significant.

The cytology data (marker expression and morphological parameters) were scaled and trained with different machine learning models. For hyper parameter tuning, 3-fold cross-validation of training data was performed and tested in 30%. The diagnostic performance was calculated for two-class system (oral cancer/HGD vs. LRL) and was assessed by comparing sensitivity, specificity and AUC in the training and test data. HGD and cancer was considered as reference standard (histology diagnosis) positive. All the machine learning protocols were performed in Python 3.7.4.

#### Sample size

The markers were positive in 70% of OPMD and 96% of oral cancer. Assuming 80% power (alpha error 5%), the minimum required sample size was 32 per group. Accordingly, 40 samples were taken in each cohort for histology/cytology validations. Descriptive statistics was used to summarize the details of patient demography, clinical features and pathology diagnosis. Kolmogorov-Smirnov test was performed to assess the normal distribution of IHC/cytology scores. All the statistical comparisons were by ANOVA (Kruskal-Wallis test; multiple groups and student’s T-test; 2 groups). P-value<0.05 (2-sided) was considered as significant. All statistical analyses were performed using MedCalc 14.8.1.

## Results

### Systematic review and meta-analysis for marker selection

The literature search returned 1165 articles/abstracts (article: 848; abstracts: 317) and the articles related to oral cancer/OPMD were screened by a sequential process and 170 of them were selected. Further, based on the inclusion and exclusion criteria, 80 articles were selected for data extraction. Among these articles, based on specific criteria (S1, S3–S5 Figs, Table 1), 46 articles were included in the quantitative analysis and 14 markers were identified for individual analysis (S1 Table). Quadas-2 assessment showed a high bias in the selection of patient cohort and interpretation of IHC (S6 and S7 Figs) in the articles. Quantitative analysis indicated that 10/14 markers showed a significant association with dysplastic-OPMD; P53, Ki-67, Podoplanin, CyclinD1, Ecadherin, PCNA, CD44, CDK4, p27 and Syndecan-1 (Table 1).

**Table 1:** Meta-analysis results of markers identified from the systematic review.

Markers	No. of studies	Odds Ratio/ Mean difference	95% confidence Interval	p-Value	I^2^ (p-Value)	No. of Subjects	AUC (sROC)
**P53 (D)**	13	6.28	4.04–9.75	<0.0001	19.9% (0.24)	897	0.775
**Podoplanin(D)**	5	10.16	3.39–30.41	<0.0001	42%(0.14)	432	0.828
**Ki67 (D)**	4	5.3	2.11–13.32	0.0004	0%(0.81)	164	0.754
**P16 (D)**	6	1.29	0.50–3.29	0.6	49%(0.08)	425	0.542
**CyclinD1 (D)**	6	5.15	1.80–14.70	0.002	23%(0.26)	279	0.804
**PCNA(D)**	3	9.4	4.66–18.84	<0.001	88%(0.0003)	270	0.991
**Ecadherin (D)**	3	0.3	0.13–0.68	0.004	0%(0.41)	176	0.371
**P21(D)**	3	0.93	0.04–19.45	0.96	85%(0.001)	248	0.658
**hTERT(D)**	3	2.42	0.24–24.48	0.45	60%(0.08)	79	0.636
**MDM2(D)**	3	1.77	0.92–3.42	0.09	44%(0.17)	221	0.721
**CDK4 (D)**	3	5.85	2.06–16.60	0.0009	0%(0.42)	158	0.791
**syndecan-1(D)**	3	0.03	0.01–0.18	<0.001	0%(0.99)	111	0.046
**p27 (C)**	3	-14.18	-22.5–5.85	0.0008	92%(<0.001	194	
**CD44s(C)**	3	24.1	23.2–24.96	<0.001	0%(0.55)	526	
**p53(C)**	4	9.06	0.94–17.18	0.03	97%(<0.001)	310	
**Ki67(C)**	5	15.41	9.59–21.22	<0.001	95%(<0.001)	395	
**PCNA(C)**	3	28.67	18.72–38.61	<0.001	55%(0.11)	126	

Represents types of data, number of studies, odd ratio/mean difference, heterogeneity (I^2^), number of patients validated and Summary ROC analysis of each marker. Odds ratio represents the result of dichotomous data. AUC: Area Under Curve, sROC: Summary of Receiver Operating characteristic analysis, D:Dichotomus data extracted, C: Continues data extracted

Subsequently, multiple criteria were applied such as non-significant heterogeneity, significant difference in the mean expression, sAUC >75% (ROC analysis), and odds ratio>5 in delineating dysplastic-OPMD from the non-dysplastic cohort. The top markers that scored based on these criteria, Podoplanin (OR: 10.16; CI: 3.39–30.41; n: 432), P53 (OR: 6.28; CI: 4.04–9.75; n: 897), CD44 (mean difference: 24.1; CI: 23.2–24.96: n: 526) and Cyclin D1 (OR: 5.15; CI: 1.80–14.70; n: 279) were carried forward (Table 1; S3 Fig).

As a next step, S100A7, hnRNPK and PTMA were selected from the review of high-throughput proteomics studies [26–28]. Individual analysis for association with dysplastic-OPMD indicated that S100A7 had the highest OR (11.2; CI: 5–24.8; n: 533), followed by PTMA (OR: 7.3; CI: 3.2–16.6; n: 270) and hnRNPK (OR: 5.8; CI: 2.5–13.4; n: 354). The specificity of all three markers was above 80%, while S100A7 and hnRNPK showed a sensitivity above 70% (PTMA: 50%). A search into the markers detecting aberrant glycosylation identified three lectins validated in dysplastic-OPMD, gastric cancer and prostate cancers; Wheat Germ Agglutinin (WGA) [17, 18], Sambucus-Nigra-Agglutinin-1 (SNA-1) [29] and Maackia-Amurensis Agglutinin (MAA) [29, 30]. The panel of markers identified from each analysis (P53, CyclinD1, Podoplanin, CD44, S100A7, hnRNPK, PTMA, WGA, SNA-1, and MAA) was carried forward for validation.

### Immunohistochemical analysis identified the marker profile correlating HGD/cancer

**Fig 3:**
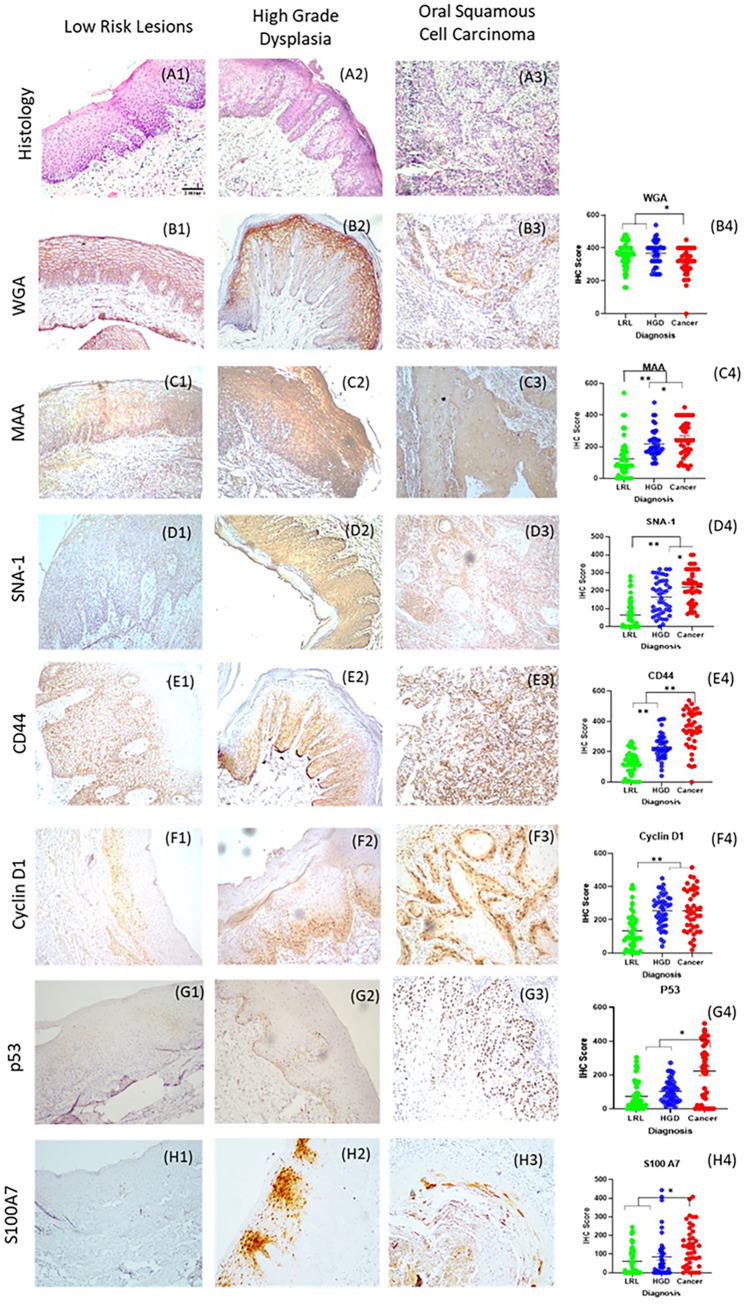
Tissue validation of markers. Histology (A1-A3), immunohistochemistry images and score of WGA (B1-B4), MAA (C1-C4), SNA-1(D1-D4), CD44 (E1-E4), CyclinD1(F1-F4) P53(G1-G4) and S100A7 (H1-H4) with scale bar 0.14 mm (objective 10x) were depicted. IHC scores of markers showed that expression in LRL is significantly less compared to HGD and/or OSCC. * <0.05; ** <0.005. ANOVA showed that SNA-1, CD44 and MAA significantly differentiated three cohorts LRL, HGD and OSCC. CyclinD1 delinated HGD OSCC from LRL. Graph represents mean±Standard error. LRL: Low Risk Lesions (Non-Dysplastic oral lesions; n: 50). HGD: High-Grade Dysplasia (Moderate/Severe Dysplasia; n: 40), OSCC: Oral Squamous Cell Carcinoma (n: 41).

**Fig 4:**
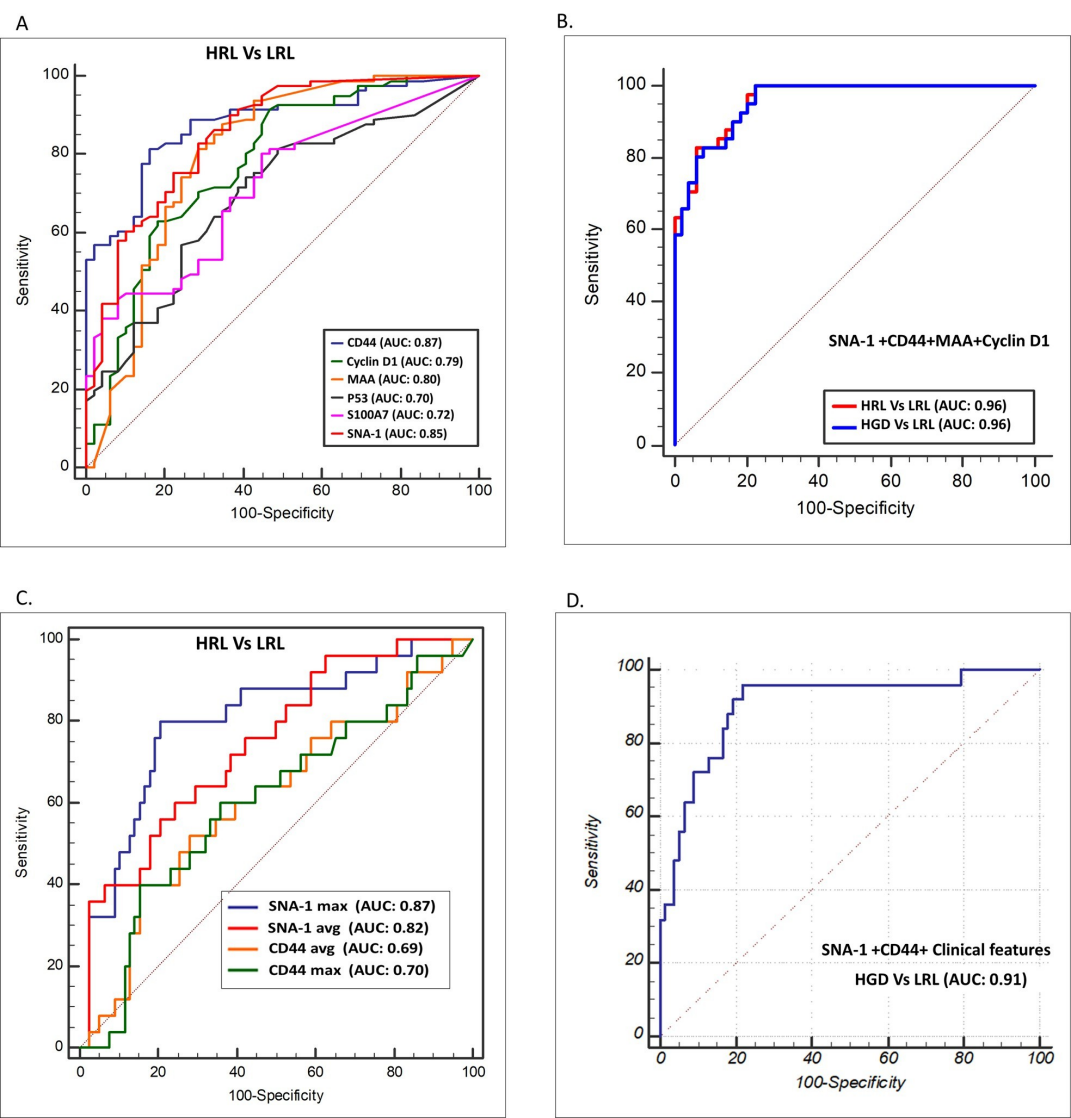
Delineating OSCC and HGD from Low Risk Lesions. Graph depicting Receiver Operating Characteristic (ROC) curve Analysis of immunohistochemistry (A,B) and multiplex-Immunocytology (C,D) validation of markers. Combination of markers delineated HRL/HGD from LRL by immunohistochemistry (AUC: 0.96). CD44 and SNA-1 showed best AUC in histology (A) and cytology (C, D) in delineating HRL and HGD. AUC: Area Under Curve, HGD: High Grade Dysplasia, HRL: High Risk Lesion (OSCC+HGD), LRL: Low Risk Lesions.

The validation of the 10-marker panel was carried out by IHC in two phases. In phase I, all markers were evaluated for the presence of at least one of the criteria; i) differentiate HGD from LRL (n: 20) ii) ROC curve analysis (AUC >0.75) and iii) sensitivity to differentiate HGD/cancer (n: 20) from LRL (>75%). Among the panel, a set of 7 markers, CyclinD1, P53, MAA, SNA-1 (fulfilled all criteria), CD44, S100A7 (AUC, sensitivity >0.75), and WGA (differentiated HGD) (S8–S10 Figs) were validated in an extended cohort (Fig 3 Phase II, n: 131; LRL: 50, HGD: 40, OSCC: 41). In the Phase II, CD44 and SNA-1 showed highest AUC (>0.85) in differentiating cancer/HGD from LRLs (Figs 3, 4A and 4B, S3 Fig, S10 Table).

Additionally, CD44, SNA-1, CyclinD1 and MAA (Fig 3) differentiated HGD from LRL (p<0.05) with high AUC (>0.75) and sensitivity (>75%), with CD44 showing the highest sensitivity (80%; CI: 69.9–88.3) and specificity (84%; CI: 70.9–92.8). Further, logistic regression analysis with the seven markers (stepwise method) identified three markers, CD44 (p<0.0001), CyclinD1 (p: 0.0017) and SNA-1 (p<0.0001) with 87.65% (CI: 78.47–93.92) sensitivity and 88% (CI: 75.69–95.47) specificity (S4 Table, AUC: 0.96; CI: 0.91–0.99). Addition of MAA to the model increased sensitivity (89%; CI: 80.19–94.86) and specificity (92%; CI: 80.40–97.73). This subset of 4 markers was selected for cytology validation (S3 Table).

### CD44 and SNA-1 delineated high grade oral lesions by cytology

Cal-27 (OSCC) and DOK (Dysplastic oral keratinocyte) cells were used to assess the profile of the four markers in addition to standardizing cytology (S11 Fig). In comparison to normal cells of healthy volunteers, SNA1 and MAA had significantly (p<0.001) higher intensity (Image J) in CAL-27 and DOK cells. (S11 Fig). CD44 showed high percentage positivity in Cal27 (90%) and DOK (40%). However, no significant difference was observed in mean intensity or percentage positivity between DOK and CAL-27.

**Fig 5:**
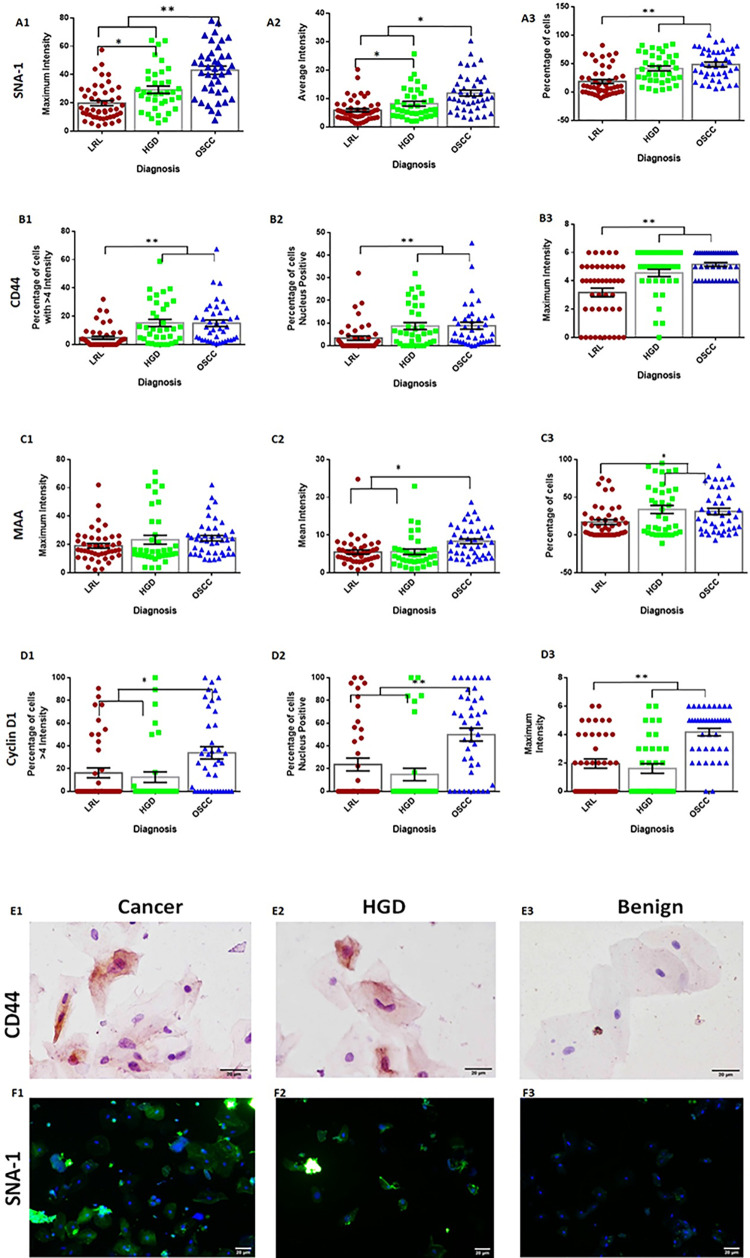
Immunocytology profile of MAA, CyclinD1, CD44 and SNA-1. SNA-1 (A1-A3) and MAA (C1-C3) expression of each patient indicating the maximum intensity, average intensity and percentage of cells showing higher in the lesion site. The profile shows a significant increase as diseases progresses. CD44 (B1-B3) and CyclinD1 (D1-D3) expression parameters, percentage of cells with higher intensity (> 4 intensity score out of 6), nuclear positivity and maximum intensity showed significantly less expression in LRL compared to HGD/OSCC. * <0.05; ** <0.005. Graph represent mean ± Standard error. LRL: Low Risk Lesions (Non-Dysplastic and mild dysplastic oral lesion). HGD: High-Grade Dysplasia (Moderate/Severe Dysplasia), OSCC: Oral Squamous cell carcinoma, HRL: High Risk Lesions (OSCC+HGD). SNA-1 (FITC conjugated and DAPI staining, E1- E3; Magnification 20x objective) and CD44 (F1-F3; Magnification 40x objective) staining of cells from OSCC, HGD and benign subjects. Images of OSCC and HGD patients shows high staining compared benign subjects.

An independent patient (n = 134; OSCC: 42; HGD: 37; LRL: 55) cohort was used for cytology validation of the 4-marker panel. Buccal mucosal lesions were a majority in the cohort (n = 97), with a mean age of 44 years (range: 19–80 years) and a gender distribution of 1.6 (M: F). History of tobacco use was observed in 70% (n = 90) of patients (S12 Fig). Among the two lectins, average (SNA-1avg) and maximum intensity score of SNA-1 (SNA-1max) significantly increased (p<0.05) from LRL to OSCC (Fig 5A1–5A3). CD44 maximum intensity (CD44max), percentage of nuclear positive cells (CD44N+) and high expression (CD44%; intensity >4) were significantly higher (p<0.005) in HGD and OSCC compared to LRL (Fig 5B1–5B3). Cyclin-D1 expression and average intensity of MAA (MAAavg) were higher in cancer cohort as compared to LRL/HGD (p<0.05). In this analysis, SNA-1 (AUC: 0.81; CI: 0.74–0.88) and CD44 (AUC: 0.76; CI: 0.67–0.83) showed high sensitivity (S13 Fig). Logistic regression analysis identified SNA-1max (p<0.001), CD44N+ (<0.001), and CyclinD1N+ (percentage of cells with nuclear positivity; p = 0.02) as the best features (Sensitivity: 84%; CI: 63.92–95.46; Specificity: 84.61%; CI: 54.55–98.08; S5 Table). Random forest also identified these features of SNA-1 and CD44 as best features (Sensitivity: 80%; CI: 59.30–93.17; Specificity: 84.62%; CI: 54.55–98.08; S6 Table).

The best markers (SNA-1 and CD44) identified by both models were combined with clinical features (age, habit history, site of lesions) to develop a classification model. Logistic regression model indicated that SNA-1max (p: 0.002), CD44N+ (p<0.001) and CD44% (p<0.049) provided a high sensitivity (87.5%; CI: 67.64–97.34) and specificity (88.23%; CI: 63.56–98.54). PCA with regularized logistic regression (L2) also provided a test sensitivity of 83% (CI: 62.62–95.26) and specificity of 94% (CI: 63.56–98.54) (Table 2, S7 Table).

**Table 2:** Comparison of different models in molecular cytology based delineation of OSCC and HGD.

*Phase I ICC*: *Single-marker combination (HRL Vs LRL)*					
	Logistic Regression	Regularized Logistic regression	Random Forest	SVM	XG_Boost
**Sensitivity**	87.5(21/24)	83.33 (20/24)	83.33(20/24)	75(18/24)	87.5 (21/24)
**Specificity**	88.23(15/17)	94.12 (16/17)	88.24(15/17)	82.35 (14/17)	70.59(12/17)
**Accuracy**	84.95	87.8	85.36	78.04	78
***Phase II ICC*: *Multiplexing (HRL Vs LRL)***					
**Sensitivity**	79.17(19/24)	91.67(22/24)	87.5 (21/24)	91.67(22/24)	83.33(20/24)
**Specificity**	84.37(27/32)	84.37(27/32)	90.62(29/32)	75(24/32)	90.62(29/32)
**Accuracy**	82.14	87.50	89.29	82.14	87.50

Represents the sensitivity specificity, predictive values and accuracy of machine learning model in differentiating HRL from LRL (manual analysis-test results) in Phase I and Phase II ICC (multiplex) validation. LRL: Non-Dysplastic oral lesion. HGD: Moderate/Severe Dysplasia, OSCC: Oral Squamous cell carcinoma, HRL: OSCC+ HGD

### CD44-SNA1 multiplexing improved the sensitivity of oral cytology

#### Nomogram

Cells were collected from three different sites (n = 45; buccal mucosa, tongue-lateral, gingiva) of healthy volunteers without risk habits (n: 15, sites: 3). The cells were incubated with TRITC-conjugated SNA-1 and FITC-conjugated CD44 (after fixing with 3% PFA) sequentially before being counterstained with DAPI (S1 Appendix). Multiplexed immuno-cytological of healthy oral subsites showed no difference in SNA-1 staining (S14A-S14C Fig). CD44, however, showed a high expression in tongue and gingiva of the elderly age group (>40 years) (S14 Fig).

#### Distinguishing HGD, OSCC from LRL

**Fig 6:**
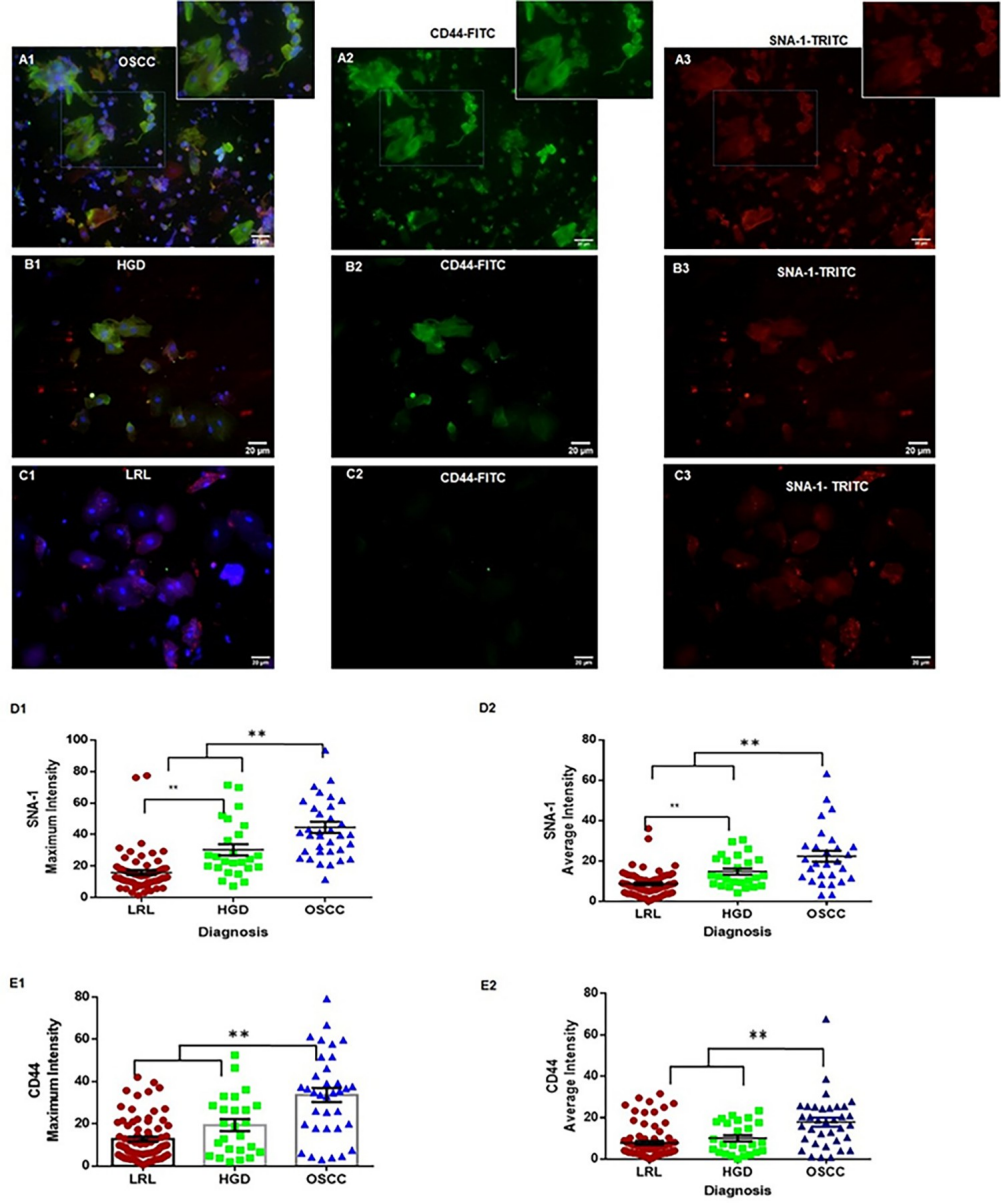
Multiplex validation of SNA-1 and CD44. Multiplex cytology images (FITC conjugated CD44; TRITC conjugated SNA-1; scale bar 20μm, Objective 20X) from the patient cohorts; OSCC (A1-A3), HGD (B1-B3) and LRL (C1-C3). SNA-1 expression (D1, D2) showed significantly high in OSCC/HGD. CD44 (E1, E2) expression showed significantly higher in OSCC. * <0.05; ** <0.005. Graph represent mean ± Standard error. LRL: Low Risk Lesions. HGD: High Grade Dysplasia, OSCC: Oral Squamous cell carcinoma, HRL: High Risk Lesions (OSCC+HGD).

Multiplex validation of CD44 and SNA-1 was performed in an independent cohort (sub-sites: 138; subjects: 102), which included OSCC (n = 35), HGD (n = 25), LRL (n = 33) and normal (n = 45). The mean age was 43 years (range: 18–75) with a gender distribution of 1.8 (M:F). While majority oral samples were collected from buccal mucosa (60%, n = 85), 67% were exposed to tobacco chewing or smoking (S12 Fig). Analysis showed significantly high expression in OSCC and HGD (Figs 4C, 4D and 6A–6E). Logistic regression analysis combining the markers with clinical features (age, habits, and site of lesion) provided a high AUC (HGD/Cancer Vs LRL: 0.90). Among the different machine learning models (S7 Table), Random Forest gave the best test sensitivity (87.5%; CI: 67.64–97.34) and specificity (91%; CI: 74.98–98.02; Table 2). SNA-1/CD44 profiling differentiated OSCC from LRL with high sensitivity (Sensitivity: 90–91%; specificity: 84–92%; AUC: 0.94–0.96; S15 and S16 Figs).

The HGD cohort was also delineated (AUC: 0.85–0.91) with high sensitivity (82–90%) but with comparatively less specificity (80–82%) (S15 Fig, Table 2). A comparison with the conventional cytology in the multiplex validation dataset (n = 122) indicated that the oral pathologist diagnosed OSCC from LRL with a sensitivity and specificity of 80% (CI: 59.30–93.17) and 76.25% (CI: 65.42–85.05) respectively. Significantly, HGDs were delineated with a sensitivity of 29.41% (CI: 10.31–55.96; S8 Table), showing that SNA-1/CD44-based cytology improved the sensitivity/specificity of detecting HGD.

### SNA-1 integrated with image analysis in delineating HGD/cancer

**Fig 7:**
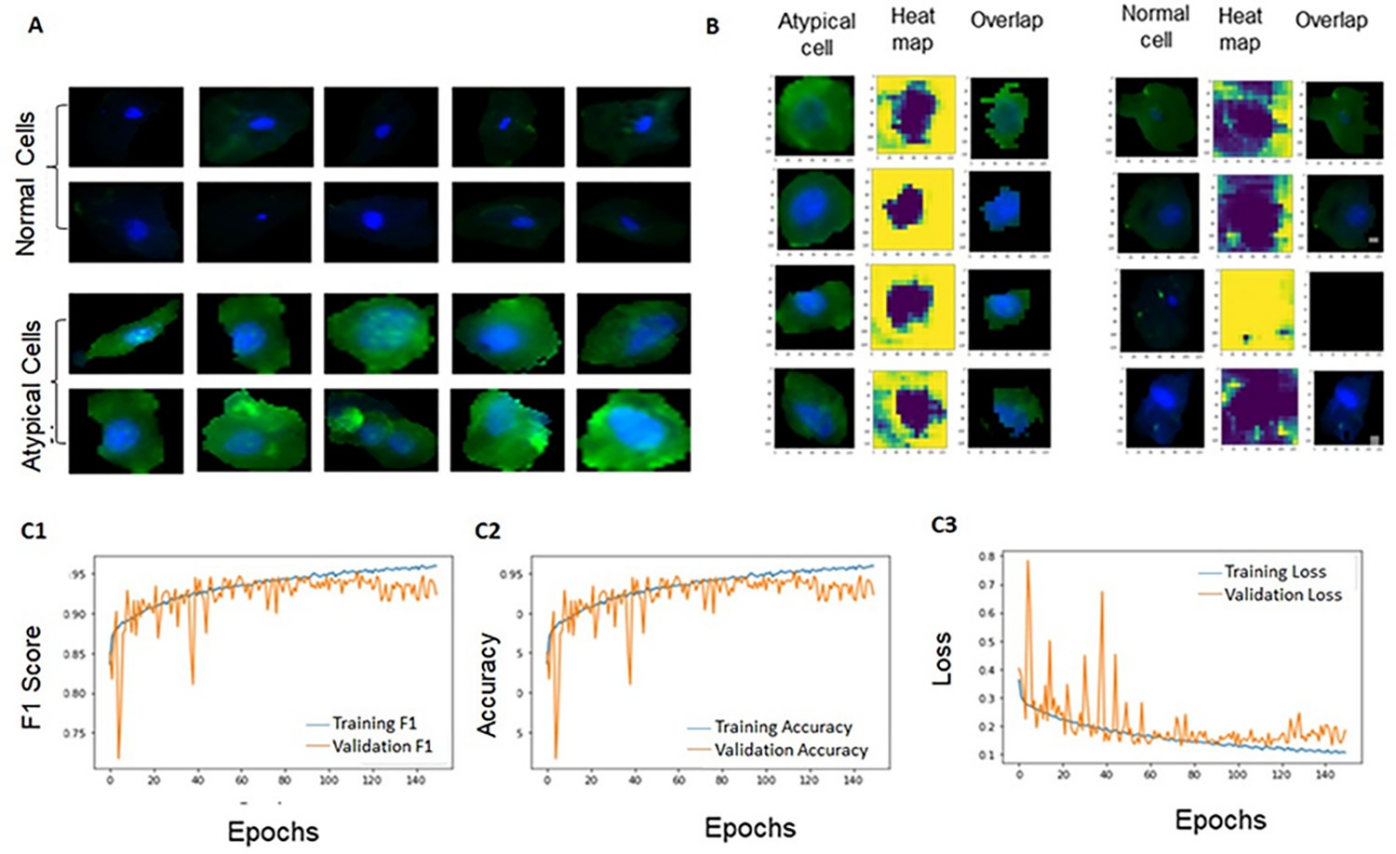
Classification of cells using Cancer-Net model. The Cancer-Net model was employed for classification of segmented single epithelial cells (A). The occlusal maps (visual representation of the regions of interest) showed that nucleus, and cytoplasm around nucleus were used by Cancer-Net model for atypical cell classification. The cell, heat map (occlusal map) and overlay with cell are depicted (B). The training/cross validation metrics for differentiating normal and atypical cells (C1-C3) showed F1Score above 0.90.

As a final step, we attempted to assess the accuracy of SNA-1, when integrated with image automation in delineating HGD/cancer. Manual analysis of the SNA-1 data set provided a sensitivity and specificity of 83% and 71% respectively (S13 Fig). The segmentation (U-Net) and classification models (Artefact-Net) previously developed were used in this study [25]. Lectin-stained cytology datasets (Fig 6A) were used for automation. The classification model was trained using the MAA-FITC dataset (Atypical cells: 730; augmented: 5692; normal cells: 1158; augmented: 8093) and tested in SNA-1 dataset (atypical cells: 53; normal cells: 143). The training for atypical cell classification was performed by Inception-V3 and Cancer-Net (Fig 7; S17 Fig). The Inception-V3 gave a high cross-validation sensitivity (90.27%) and specificity (99.8%), however the model showed less test sensitivity (SNA-1: 75.47%: S9 Table). The Cancer-Net model showed a similar cross-validation sensitivity (90.81%) and specificity (96.22%), but with a higher test sensitivity (88.6%) and specificity (97.90%) for delineating atypical cells from normal cells (Fig 7; S9 Table).

14,357 images were segmented out as cells and clusters from the SNA-1 data set using U-Net [25]. The segmented images were categorized by the Artefact-Net model with 88.16% of images as cells (62.99%; n: 9044) and clusters (25.17%; n: 3614) [25]. The statistical aggregates of mean, maximum, and standard deviation, of cellular features (n: 16) and the Cancer-Net prediction statistics (ratio of cancer/normal cells; average prediction score of cancer cells) were considered as features (S10 Table). The variation of data among the cohorts (LRL, HGD, cancer) was evaluated by Kruskal Wallis and 29 features selected for machine learning using multiple models (S10 Table). SNA-1 intensity and Cancer-Net prediction score measured from segmented cells increased as disease progresses same as manual analysis (S18 Fig). Nuclear cytoplasmic area ratio, convex area of the cell, and convex area of nucleus increases as the disease progresses (LRL-HGD-Oral cancer: S19–S21 Figs).

The important features (S11 and S12 Tables) identified were average neural-network prediction score of atypical cells (p = 0.001), nuclear-cytoplasmic area ratio (p = 0.016), Min-axis ratio (p = 0.008), and maximum convex area of cells (p = 0.09) by tuning VIF and significance by Logistic regression model. The model gave a high training sensitivity (89%)/specificity (82%) as well as test sensitivity (87%) and specificity (82%). *PCA with Regularized Logistic regression Model*: All features were used in this model after dimension reduction with PCA. Ten Principal components, L2 regularization, and ‘logistic_c’ as 2 were selected as best parameters on cross validation. The training sensitivity/specificity mirrored the logistic regression model while the test sensitivity and specificity were 83% and 88% respectively. *Random Forest Model*: The most important features identified were the neural network prediction score, convex area of nucleus, eccentricity of the nucleus, minimum axis ratio, nuclear-cytoplasmic area ratio, and SNA-1 intensity. The model was over-fit with test sensitivity (83%) and specificity (88%) same as Regularized Logistic regression model (S12 Table).

## Discussion

Cytopathology in oral cancer, despite being a minimally-invasive assay for early detection, has been challenged by subjectivity in interpretation [11] and lack of well-defined diagnostic criteria. Several methods including OralCDx, OralCyte and ClearPrep improved brush biopsy, however diagnostic value for dysplasia has been 30–40% [31]. A study from our group reported that liquid-based conventional cytology delineated HGD with 25% sensitivity [11]. The present study addresses the poor diagnostic accuracy of oral cytology and the critical lacunae that limits its wider clinical application by applying molecular markers as an adjunct. Herein, markers selected from a systematic review were validated sequentially by histochemistry and cytology to arrive at a panel that can improve accuracy of oral cytology. Our study revealed that Cyclin D1, CD44, MAA and SNA-1 offered a sensitivity of 89% and specificity of 92% in delineating HGD and oral cancer. In cytology, classification models integrating clinical parameters with SNA-1 (detects aberrant glycosylation) and CD44 (glycoprotein) differentiated OSCC/HGD from LRL with high sensitivity /specificity (>84%; n: 272). Multiplexed cytology models as well as SNA-1 integrated with image automation indicated the significance of molecular markers in improving oral cytology-based detection.

Meta-analysis evaluating diagnostic efficacy of markers assessed by IHC in delineating dysplastic-OPMD are comparatively few. Our study is the first, to the best of our knowledge, to identify biomarkers (n = 10) associated with dysplasia through meta-analysis. A recent meta-analysis identified Ki67 as capable of distinguishing degrees of dysplasia in actinic cheilitis patients, albeit with high heterogeneity [32]. The risk of bias/heterogeneity was high in our study, mostly attributed to variations in IHC scoring method. Further, QUADAS assessment indicated a high risk of bias in the interpretation of index test and applicability in case selection. This was primarily due to a majority of the studies focusing on clinical diagnosis as the reference standard, while our question was pertaining to delineating dysplasia. This challenge was addressed by carrying out sensitivity analysis to reduce the heterogeneity. After the sensitivity analysis, CD44 and P53, investigated in maximum number of dysplastic-OPMD subjects (CD44: 526; P53: 897) with a higher AUC (0.80), were carried forward for validation in cytology. A recent meta-analysis identified that P53 overexpression was associated with a two-fold risk of malignant transformation of OPMD [33], while CD44 is a well-established marker in many solid malignancies promoting tumour cell growth and migration [34]. As observed in our review, multiple studies have reported increased expression in OPMD [35] and with dysplastic progression [23], however their relevance in cytology-based assessment is not known.

Lectins, which detect aberrant glycosylation patterns, have been identified as markers of neoplastic changes in many cancers [17, 29, 30]. This study identified three lectins (WGA, MAA and SNA-1) reported as differentials in multiple cancers in various studies including ours [19], wherein local application of WGA on oral lesions significantly differentiated OPMD. However, our current results indicate that WGA (binds to N-acetyl D-Glucosamine and sialic acid) could not significantly differentiate HGD from LRL by histochemistry, indicating a difference in histological staining as compared to topical application. MAA is known to specifically bind to alpha-2, 3 sialic acid, while SNA-1, elderberry bark tree lectin (Sambucus Nigra; Elderberry Bark), detects the aberrant glycosylation in alpha-2, 6 sialic acid; both reported previously in detection of prostate cancer [29, 30]. In our study, SNA-1 staining pattern in cytology significantly increased as the disease progressed from LRL to OSCC, while MAA showed high sensitivity in detection of HGD/OSCC indicating the significance of specific glycosylation patterns (α 2,6 and α 2,3 linkages) in oral cancer progression. Although further studies are essential to delineate the exact role of lectins during carcinogenesis, this study clearly identified SNA-1 as a significant adjunct to delineate HGD.

In a recent study [36], oral cytology was shown to have sensitivity and specificity of 79% and 94%, respectively. However, the study cohort had low representation of HGD (12%) and OSCC (6.7%). Biomarker-based oral cytology has been attempted in a few studies, McM2 and Laminin γ2 chain in pre-invasive or invasive squamous cells in brush biopsies showed an increased sensitivity when compared to conventional cytology [13]. A recent study also reported that αvβ6, EGFR, CD17, McM2, Geminin and Ki-67 could differentiate oral cancer and HGD with 78% sensitivity [9]. Our study pointed out a definite clinical relevance of markers in cytopathology, wherein a two-marker panel of CD44 and SNA-1 improved the efficacy of cytology-based delineation of OSCC/HGD when used individually with clinical parameters (Sensitivity: 83%) and upon multiplexing (Sensitivity: 92%).

The nomogram of biomarker cytology can help to identify significant confounding factor/s given the heterogeneity in the sites of oral cavity and patient clinical parameters. CD44-SNA1 nomogram indicated that CD44 expression correlated with sites and age (high in tongue, gingiva with age >40 years). Interaction of markers with clinical parameters was hence included during feature engineering and development of the integrated classification model. In our study, combining clinical parameters with CD44-SNA1 multiplexed oral cytology profile gave a high sensitivity (92%) and specificity (84%). This data analysis pipeline combining marker profile with clinical parameters, for cytology-based diagnosis of HGD and cancer, has not been attempted previously.

Image automation approach in cytology enables integration of multiple parameters, objective assessment, and thereby improved accuracy. In our previous study [11] we have used InceptionV3 for the classification of Hematoxylin-Eosin stained atypical cells from normal cells with sensitivity of 73% in HGD- OPMD. The transfer learning model used in the current study showed slightly over fit in the test data. In this study, we used the Cancer-Net for feature extraction and classification of atypical cells. The logistic regression model provided the best test sensitivity (87%) and specificity (82%), an accuracy which was an improvement on the manual analysis of SNA-1-based oral cytology. The most significant features identified were neural network prediction score, nuclear-cytoplasmic area ratio, minimum axis ratio, and cell convexity. These features [11] were identified as important in the previous study for the classification of cancer/atypical cells by pathologist’s interpretation, however they could not delineate HGD. In our study, Random Forest provided a good training sensitivity/specificity of 95% with bootstrap sampling. Previous studies using molecular markers- EGFR, Ki67 had an accuracy of 70% [37] for delineation of cancer and pre-cancer lesions. The risk stratification machine learning model using Logistic regression (L1 regularization) combining morphological parameters and molecular markers-αvβ6, EGFR, CD17, McM2, geminin, and Ki-67 differentiated cancer and HGD from LRL with sensitivity and specificity of 78% and 88% respectively [9]. Our study provides an improvement on oral cytology with SNAI-based cytopathology integrated with automated image analysis (Sensitivity: 86%). Automated image analysis of multiplexed images will improve the accuracy further, studies are currently ongoing to develop the pipeline. The markers were tested in independent cohorts over the course of two phases, and automation produced results that were comparable; nevertheless, the study still requires external validation.

In conclusion, use of biomarkers in oral cytology improves the accuracy of the approach in risk-stratification of the high-grade lesions. Our study indicated that use of a biomarkers (CD44/SNA-1) integrated with clinical parameters or SNA-1 with automated image analysis improved the accuracy to >85%, while multiplexed 2-marker panel analysis further improved it to >90%. Given that implementation of oral cytology in a large scale will be extremely relevant and feasible towards oral cancer early detection and down-staging of the disease, identifying specific markers and establishing their clinical relevance is a significant step towards developing a pathology-equivalent, point-of-care diagnostic tool.

## Supporting information

S1 Fig PRISMA flow chart and eligibility criteria. The Pubmed search terms returned 848 articles and 317 abstracts, which were taken forward after reviewing titles (A). The studies on unrelated research questions, studies on non-human tissues/cell lines, prognostic studies, and articles that did not report OPMD cases were removed after reviewing the abstract. A total of 170 full-text research articles were assessed based on the inclusion/exclusion criteria (B) and 80 studies were selected. For the quantitative data synthesis (46 articles), markers with a minimum of three studies, were selected for marker-wise meta-analysis. N: number; D-OPMD: Dysplastic Oral Potentially Malignant Disorders; ND-OL: Non-Dysplastic Oral Lesions, IHC: Immunohistochemistry.
(TIF)

S2 Fig Workflow of sample collection and ICC (phase I). The cytology brush rotated (cervical brush) 10 times for an ulcerated/proliferative lesion or more than 25 times using cervical cytology brush/orocellex brush for other lesions till seeing a blood tint on the site (A). If the mouth opening of the patients was less than two finger width or lesion on floor of mouth, palate and retro molar trigone Rover orocellex brush was used. The tip of brush was then triturated into a cell preservative solution (in 1.5 ml Eppendorf tube) by rotating 10–15 times in one direction and stored in BD SurePath (B; cell preservative) before experiment. Immunocytology was carried out using two standard protocols using selected markers. The cytology slides were prepared using the Cytospin (Thermo-Scientific) at 500rpm for 5 minutes. The prepared slides were then incubated with the primary antibody (C; HRP-DAB method) as per specific dilutions for 1 hour and staining was detected using the secondary detection system (Dako Real Envision, K5007). A known positive and negative control was stained for each antibody to confirm the presence of appropriate immunostaining activity. Staining in the nucleus, cytoplasm, and/or cell membranes indicated positive expression. The slides were visualized at 200x and 400x magnification (Nikon Eclipse E200) and the intensity, pattern of staining and percentage positivity were assessed (15 images/slide; 200x) (Nikon DSFi2 and NIS elements D4 20.0). For the Lectin molecules (C; SNA-1/MAA; FIC conjugated) the slide was washed with phosphate buffered saline (PBS) and incubated with Lectin markers for 30 minutes and counterstained with the nuclear stain DAPI. Images were taken using fluorescent microscope (D; Zeiss C, Axiocam and Zen lite 2012), the intensity of uptake measured (50–70 cells, Image J) and compared across the different assays/samples (D).
(TIF)

S3 Fig Forest plot analysis of selected markers. Forest plot analysis of best markers- P53 (A), Podoplanin (B), CyclinD1 (C) and CD44 (D) with non-significant heterogeneity. Podoplanin showed the highest odds ratio in differentiating D-OPMD from ND-OL. ND-OL: Non-Dysplastic Oral Lesions, D-OPMD: Dysplastic-Oral Potentially Malignant Disorders.
(TIF)

S4 Fig Forest plot analysis of other markers (not selected for IHC validation). The Figure depicts the forest plot analysis of markers: Ki-67, p16, p27.E-Cadherin, P21, hTERT, MDM2, CDK4, and Syndecan. The forest plots show the Odds Ratio and heterogeneity in the study. Ki67, E Cadherin, CDK4, and Syndecan showed significant Odds Ratio and non-significant heterogeneity.
(TIF)

S5 Fig Summary Receiver Operating Characteristic (sROC) curve analysis of markers which showed dichotomous data presentation. Representing sROC analysis showing Area Under Curve (AUC) and standard error of markers. P53, Cyclin D1, Podoplanin, CDK4, E-Cadherin, hTERT, Ki-67, MDM2, p16, P21, PCNA, and Syndecan. PCNA, CycinD1, Podoplanin, CDK4, and Ki67 showed the high AUC (>0.75).
(TIF)

S6 Fig Funnel plot. Depicting the funnel plot analysis of markers involved in meta-analysis. Most of the studies showed asymmetry and the potential risk of bias. The continuous data of all the markers showed less asymmetry.
(TIF)

S7 Fig Summary of bias risk assessment results for QUADAS-2. Quadas-2 showed high risk of bias and uncertainty in the index test. The IHC scoring is highly heterogeneous in different studies increased the risk of bias and uncertainty in Index test. H: High; L: Low; U: Unclear.
(TIF)

S8 Fig Box whisker plot of IHC analysis of Phase I IHC validation. IHC score distribution of markers CyclinD1, P53, s100A7, CD44, Podoplanin, hnRNPK, PTMA, WGA, SNA-1 and MAA. The graph showed the distribution of Low-Risk Lesions (LRL), High Grade Dysplasia (HGD), and Oral Squamous Cell Carcinoma (OSCC). P53, CyclinD1, S100A7, SNA-1 and MAA significantly higher expression in HGD and OSCC. The graph represents mean ± standard error.
(TIF)

S9 Fig ROC curve analysis of IHC markers in Phase I IHC study. Receiver operating characteristic (ROC) curves of markers (n = 10) in differentiating Low-Risk Lesions (LRL) from High Grade Dysplasia (HGD), and Oral Squamous Cell Carcinoma (OSCC). SNA-1 (AUC = 0.92) and S100A7 (AUC = 0.85) had the highest Area Under Curve, which significantly differentiate LRL (n = 20) from HRL (HGD+OSCC; n = 20).
(TIF)

S10 Fig ROC curve analysis of IHC markers in Phase II IHC study. Receiver operating characteristic (ROC) curves of markers (n = 7) in differentiating Low-Risk Lesions (LRL) from High-Grade Dysplasia (HGD), and Oral Squamous Cell Carcinoma (OSCC). SNA-1 (AUC = 0.85), CD44 (AUC = 0.87), MAA (AUC = 0.80) and CyclinD1 (AUC = 0.79) highest Area Under Curve (AUC), which significantly differentiate LRL (n = 50) from HRL (HGD+OSCC; n = 81).
(TIF)

S11 Fig Marker expression in cell lines and normal cells. Fluorescent Images (A) showing MAA and SNA-1 staining of cultured cell lines (CAL 27/DOK) and oral epithelial cells from normal subject. Images being taken with fluorescent microscope (Magnification: 200x, scale bar = 0.09mm). Immunocytology images showing the CD44 and CyclinD1 staining of CAL27/DOK and normal epithelial cells (magnification: 100X; scale bar = 0.14mm, Inset: 400X). Box and whisker plot showing the intensity differences of cells in the cell lines in comparison with the buccal cells from normal subject of MAA and SNA-1. CAL 27, DOK shows significantly high staining of markers compared to normal oral epithelial cells (p<0.0001).
(TIF)

S12 Fig Demographics of the patient cohort validated by immunocytology. Age distribution (A) of patients in the two validation phases does not show a significant difference, however, patients in both phases showed a high median age. Patients with tobacco chewing/smoking history were higher in OSCC/HGD cohort (B) and tobacco history was less noticed in phase II of LRL cohort. Gender distribution depicts (C) that the male-female ratio was high in both phases. The majority of the oral lesions (D) were present in buccal mucosa in both the phases of ICC. LRL: Non-Dysplastic oral lesion. HGD: Moderate/Severe Dysplasia, OSCC: Oral Squamous cell carcinoma.
(TIF)

S13 Fig Logistic regression analysis of individual marker (Phase I ICC). The individual marker features (SNA-1/MAA: mean intensity, maximum intensity and percentage of cells; CD44/Cyclin D1: N+, maximum intensity and percentage of cells with high intensity) were evaluated for single marker efficacy by logistic regression analysis. The features showed higher Receiver Operating-Area Under Curve (AUC) for SNA-1 (0.81) and CD44 (0.71), with high sensitivity (>80%).
(TIF)

S14 Fig Nomogram of markers (Phase II ICC): Multiplexed immuno-cytological analysis of baseline intensity carried out in the cells of healthy volunteers (n = 15; sites: 45) with regard to age-specific (A, B) and site (C) changes, showed no-significant difference in SNA-1 staining (**A-C**). CD44, however, showed high expression in tongue and gingiva sites in the elderly age group (>40 years; B, **D**).
(TIF)

S15 Fig Receiver operating characteristic curve analysis of combination of markers in delineating cancer and HGD from LRL (Phase I ICC). Combination of marker features (CD44 and SNA-1) (logistic regression analysis) differentiate cancer from Low Risk Lesions (LRL) with a sensitivity of 90% (A; AUC: 0.94). High Grade Dysplasia (HGD) was differentiated from LRL with a sensitivity and specificity >80% (B; AUC: 0.85).
(TIF)

S16 Fig Receiver operating characteristic curve analysis of combination of markers in delineating cancer and HGD from LRL (Phase II ICC). Multiplex validation of marker features (CD44 and SNA-1; logistic regression analysis) differentiated Cancer from Low Risk Lesions (LRL) with a sensitivity and specificity greater than 90% (A; AUC: 0.96). High Grade Dysplasia (HGD) cohort was also differentiated from LRL with a sensitivity of 90% (B; AUC: 0.91).
(TIF)

S17 Fig Atypical cell classification using inception V3 and Cancer-Net models. Graph depicting training and cross-validation F1 Score, accuracy and total loss of Inception V3 and Cancer Net model.
(TIF)

S18 Fig Box and whisker plot analysis of SNA-1 and DAPI expression. Graph depicting the intensity scores of SNA-1 and DAPI markers in LRL, HGD, and cancer evaluated after cell segmentation (SNA-1 data set, Phase I ICC).
(TIF)

S19 Fig Box and whisker plot analysis of cell measurement ratio. Graph depicting the different cellular and nuclear measurements and their ratios in LRL, HGD and cancer from SNA-1 stained single epithelial cells evaluated after cell segmentation (SNA-1 data set, Phase I ICC).
(TIF)

S20 Fig Box and whisker plot analysis of neural network features. The graph depicting the average, standard deviation, maximum and ratio of atypical cells values of Cancer Net Model (SNA-1 data set, Phase I ICC).
(TIF)

S21 Fig Heat map showing correlation between features. Correlation between features (SNA-1 data set Phase I ICC). Lectin intensity = SNA-1 Intensity; N_C ratio = Nuclear cytoplasmic area ratio, prob_cancer = CancerNet prediction, Maj_axis = Major axis ratio, Min_axis = Minor axis ratio. Class (Cancer/HGD = 1; LRL = 0).
(TIF)

S1 Table Data extracted from articles for meta-analysis. Data included author details, year of publication, marker, Pubmed ID and case number both in Non-Dysplastic Oral Lesions (ND-OL) and Dysplastic Oral Potentially Malignant Disorders (D-OPMD).
(DOCX)

S2 Table Details of the markers selected for IHC and ICC validation. Marker details, binding property, dilution factor and methodology used for analysis.
(DOCX)

S3 Table Immunohistochemical analysis of Phase II validation. Sensitivity, Specificity and Receiver Operating Characteristic Curve (ROC-AUC) of the markers in differentiating LRL from OSCC/HGD is shown. Table also depicts IHC scores of different cohorts.
(DOCX)

S4 Table Logistic regression model combining all IHC markers by step-wise method. Best combination of markers selected by logistic regression were CD44, Cyclin D1 and SNA-1. These markers were significant and showed a Receiver Operating Characteristic Curve Area under Curve (ROC-AUC) of 0.95 in delineating LRL from HGD/OSCC.
(DOCX)

S5 Table Logistic regression model of Phase 1 ICC. Selection of best combination of markers was performed by logistic regression analysis. The best markers selected were SNA-1, CD44 and Cyclin D1 with a test sensitivity and specificity of 84% (AUC = 0.88).
(DOCX)

S6 Table Random forest model for feature selection. Random Forest model was given sensitivity and specificity of 80% and 85% respectively. The most important features selected were SNA-1 and CD44 in delineating LRL from HGD/OSCC (HRL).
(DOCX)

S7 Table Training and test results of machine learning models for ICC validation. Multiple machine learning models were trained and validated using single marker Phase I ICC (SNA-1 +CD44) and multiplex marker Phase II ICC in delineating LRL from HGD/OSCC (HRL).
(DOCX)

S8 Table Phase II ICC vs conventional cytology. Molecular multiplex cytology was compared with conventional Haematoxylin and Eosin slides interpreted by pathologist.
(DOCX)

S9 Table Validation and testing of neural network for atypical cell classification. Sensitivity and specificity of neural network for classification of cancer cells from normal cells.
(DOCX)

S10 Table Normality and ANOVA test of patients wise features. The normal distribution and variance among cohorts were tested using the Normality test and ANOVA respectively. The colored rows showed significant features (p<0.05). Lectin intensity = SNA-1 Intensity; N_C ratio = Nuclear cytoplasmic ratio, prob_cancer = CancerNet prediction, Maj_axis = Major axis ratio, Min_axis = Minor axis ratio.
(DOCX)

S11 Table Logistic regression model. Final model developed by tuning the VIF and significance. VIF = Variation Inflation Factor (automated Phase I ICC SNA-1 data).
(DOCX)

S12 Table Comparison of models. Sensitivity, specificity of machine learning models for classifying oral cancer and HGD from LRL. TP = true positive; TN = True Negative; FP = False Positive; FN = False Negative (automated Phase I ICC SNA-1 data).
(DOCX)

S1 Appendix (DOCX)
