# Supplementary figures and images for: CD44-SNA1 integrated cytopathology for delineation of high grade dysplastic and neoplastic oral lesions
Source: PLoS One. 2023 Sep 25;18(9):e0291972. doi: 10.1371/journal.pone.0291972 (PMC10519609; doi:10.1371/journal.pone.0291972)

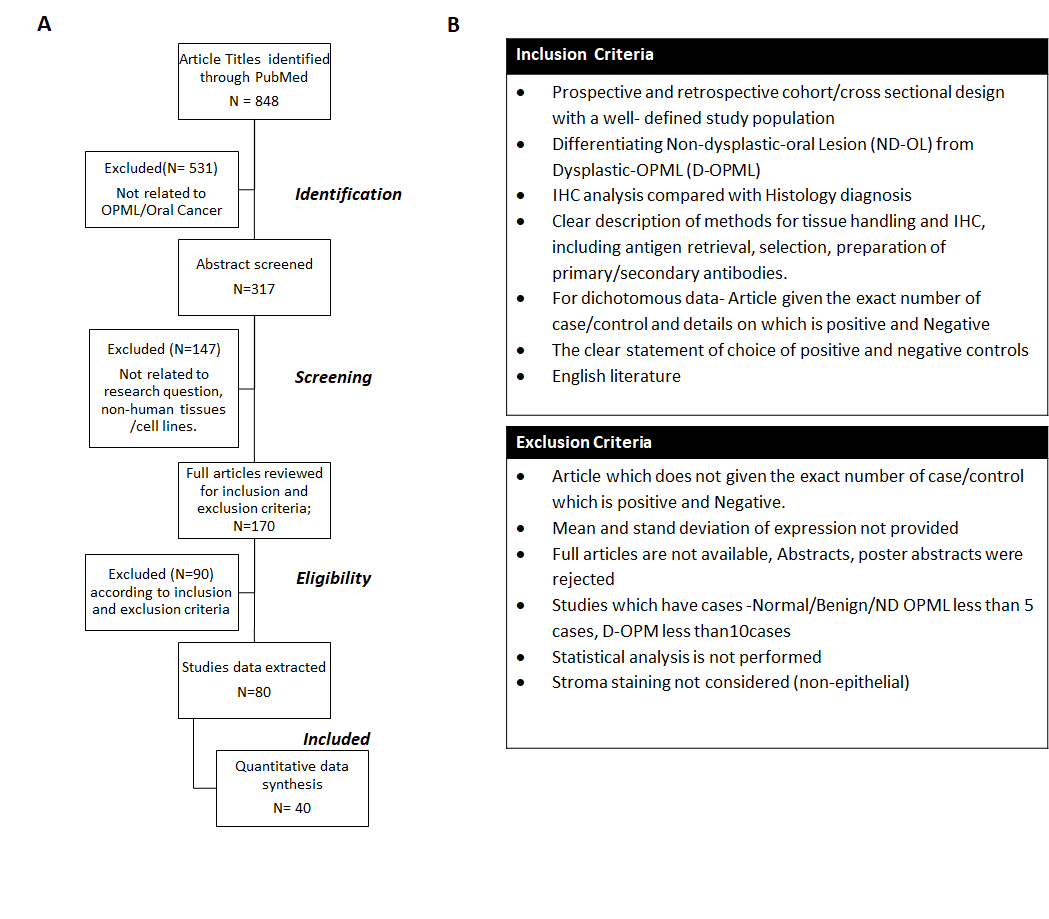

Supplement: S1 Fig — The Pubmed search terms returned 848 articles and 317 abstracts, which were taken forward after reviewing titles (A). The studies on unrelated research questions, studies on non-human tissues/cell lines, prognostic studies, and articles that did not report OPMD cases were removed after reviewing the abstract. A total of 170 full-text research articles were assessed based on the inclusion/exclusion criteria (B) and 80 studies were selected. For the quantitative data synthesis (46 articles), markers with a minimum of three studies, were selected for marker-wise meta-analysis. N: number; D-OPMD: Dysplastic Oral Potentially Malignant Disorders; ND-OL: Non-Dysplastic Oral Lesions, IHC: Immunohistochemistry. (TIF) [file pone.0291972.s001.tif]

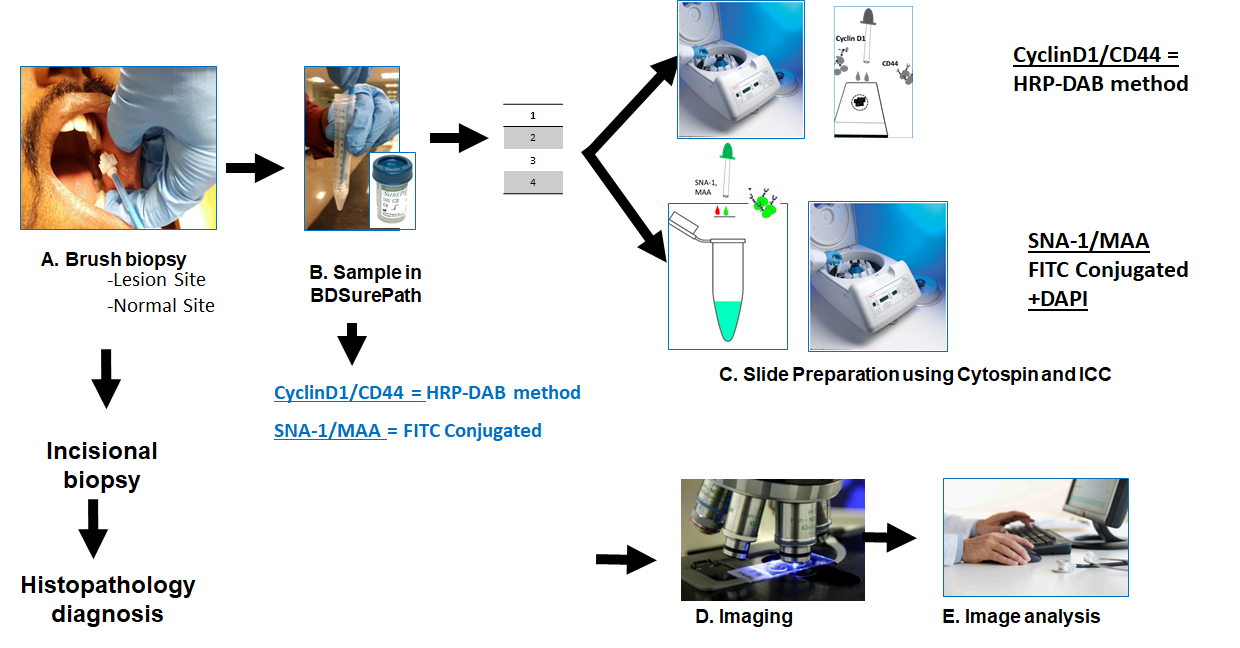

Supplement: S2 Fig — The cytology brush rotated (cervical brush) 10 times for an ulcerated/proliferative lesion or more than 25 times using cervical cytology brush/orocellex brush for other lesions till seeing a blood tint on the site (A). If the mouth opening of the patients was less than two finger width or lesion on floor of mouth, palate and retro molar trigone Rover orocellex brush was used. The tip of brush was then triturated into a cell preservative solution (in 1.5 ml Eppendorf tube) by rotating 10–15 times in one direction and stored in BD SurePath (B; cell preservative) before experiment. Immunocytology was carried out using two standard protocols using selected markers. The cytology slides were prepared using the Cytospin (Thermo-Scientific) at 500rpm for 5 minutes. The prepared slides were then incubated with the primary antibody (C; HRP-DAB method) as per specific dilutions for 1 hour and staining was detected using the secondary detection system (Dako Real Envision, K5007). A known positive and negative control was stained for each antibody to confirm the presence of appropriate immunostaining activity. Staining in the nucleus, cytoplasm, and/or cell membranes indicated positive expression. The slides were visualized at 200x and 400x magnification (Nikon Eclipse E200) and the intensity, pattern of staining and percentage positivity were assessed (15 images/slide; 200x) (Nikon DSFi2 and NIS elements D4 20.0). For the Lectin molecules (C; SNA-1/MAA; FIC conjugated) the slide was washed with phosphate buffered saline (PBS) and incubated with Lectin markers for 30 minutes and counterstained with the nuclear stain DAPI. Images were taken using fluorescent microscope (D; Zeiss C, Axiocam and Zen lite 2012), the intensity of uptake measured (50–70 cells, Image J) and compared across the different assays/samples (D). (TIF) [file pone.0291972.s002.tif]

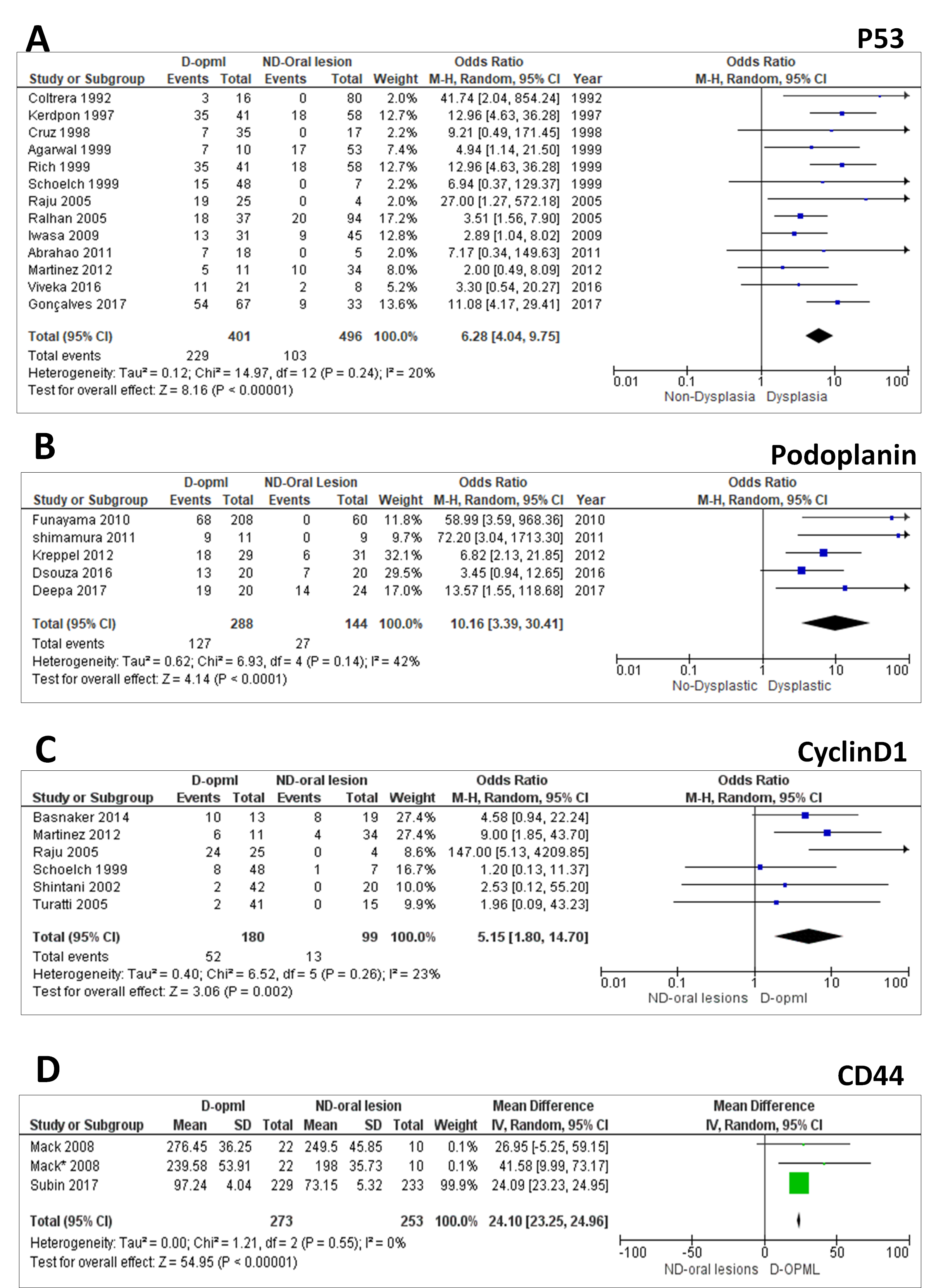

Supplement: S3 Fig — Forest plot analysis of best markers- P53 (A), Podoplanin (B), CyclinD1 (C) and CD44 (D) with non-significant heterogeneity. Podoplanin showed the highest odds ratio in differentiating D-OPMD from ND-OL. ND-OL: Non-Dysplastic Oral Lesions, D-OPMD: Dysplastic-Oral Potentially Malignant Disorders. (TIF) [file pone.0291972.s003.tif]

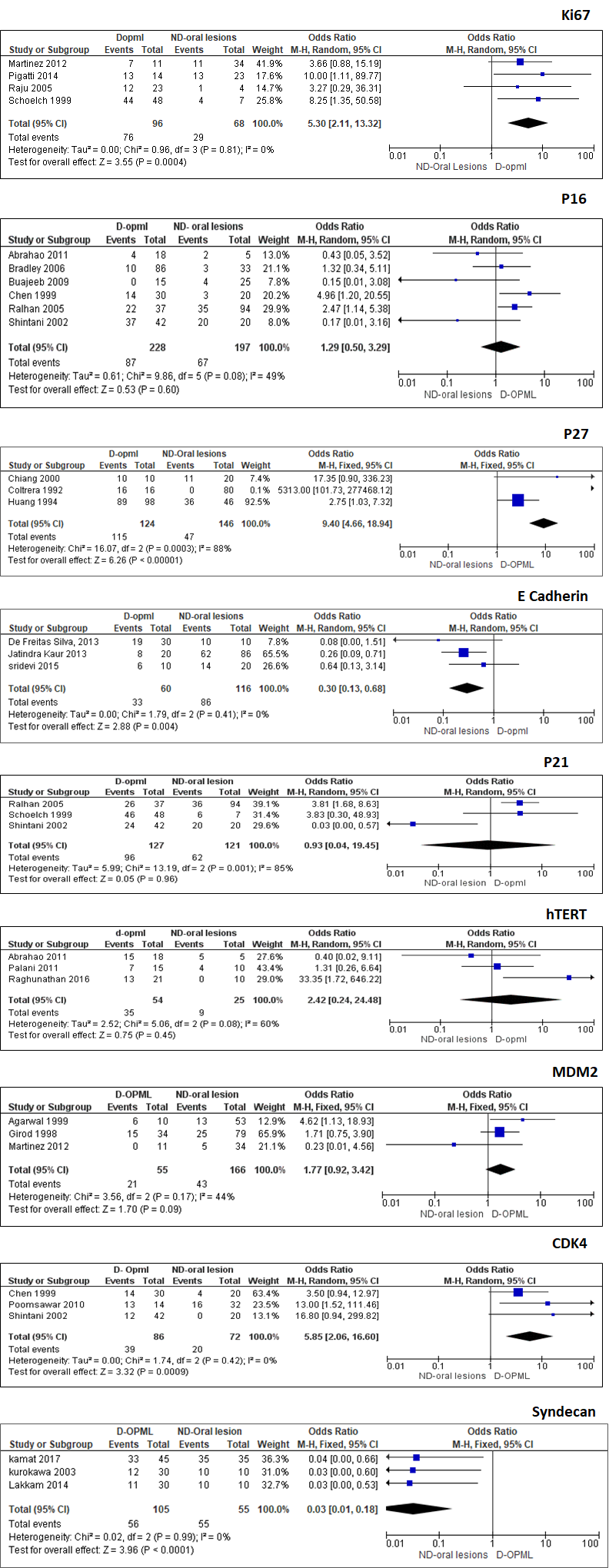

Supplement: S4 Fig — The Figure depicts the forest plot analysis of markers: Ki-67, p16, p27.E-Cadherin, P21, hTERT, MDM2, CDK4, and Syndecan. The forest plots show the Odds Ratio and heterogeneity in the study. Ki67, E Cadherin, CDK4, and Syndecan showed significant Odds Ratio and non-significant heterogeneity. (TIF) [file pone.0291972.s004.tif]

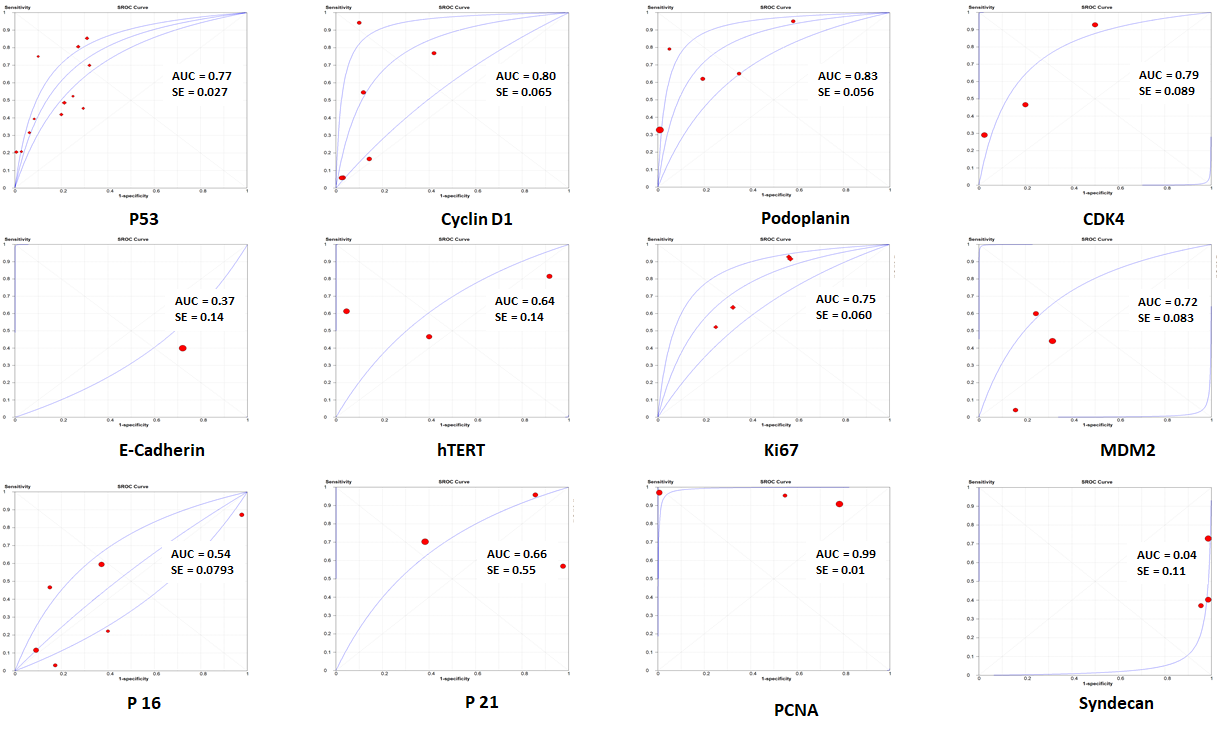

Supplement: S5 Fig — Representing sROC analysis showing Area Under Curve (AUC) and standard error of markers. P53, Cyclin D1, Podoplanin, CDK4, E-Cadherin, hTERT, Ki-67, MDM2, p16, P21, PCNA, and Syndecan. PCNA, CycinD1, Podoplanin, CDK4, and Ki67 showed the high AUC (>0.75). (TIF) [file pone.0291972.s005.tif]

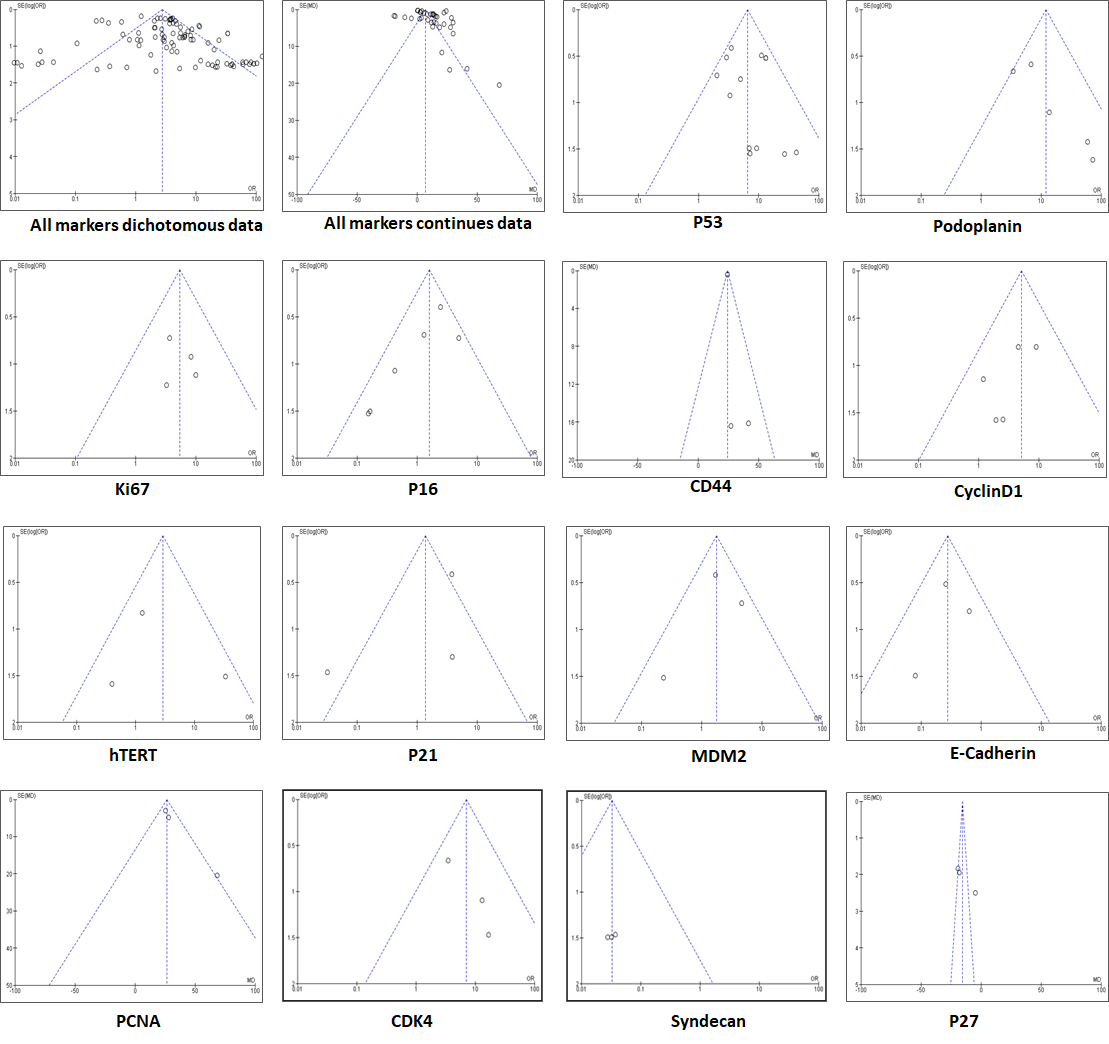

Supplement: S6 Fig — Depicting the funnel plot analysis of markers involved in meta-analysis. Most of the studies showed asymmetry and the potential risk of bias. The continuous data of all the markers showed less asymmetry. (TIF) [file pone.0291972.s006.tif]

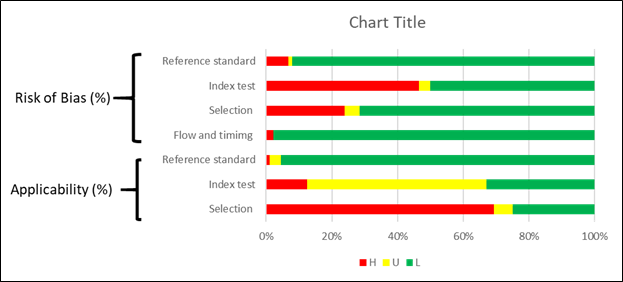

Supplement: S7 Fig — Quadas-2 showed high risk of bias and uncertainty in the index test. The IHC scoring is highly heterogeneous in different studies increased the risk of bias and uncertainty in Index test. H: High; L: Low; U: Unclear. (TIF) [file pone.0291972.s007.tif]

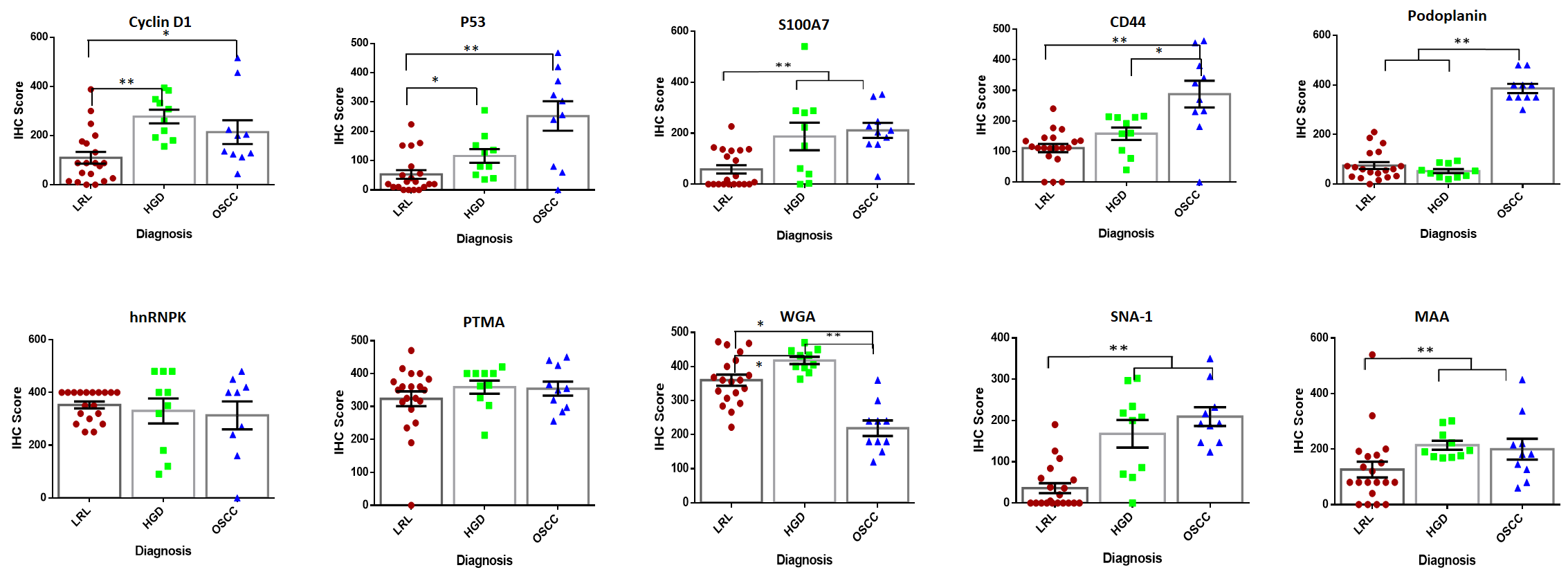

Supplement: S8 Fig — IHC score distribution of markers CyclinD1, P53, s100A7, CD44, Podoplanin, hnRNPK, PTMA, WGA, SNA-1 and MAA. The graph showed the distribution of Low-Risk Lesions (LRL), High Grade Dysplasia (HGD), and Oral Squamous Cell Carcinoma (OSCC). P53, CyclinD1, S100A7, SNA-1 and MAA significantly higher expression in HGD and OSCC. The graph represents mean ± standard error. (TIF) [file pone.0291972.s008.tif]

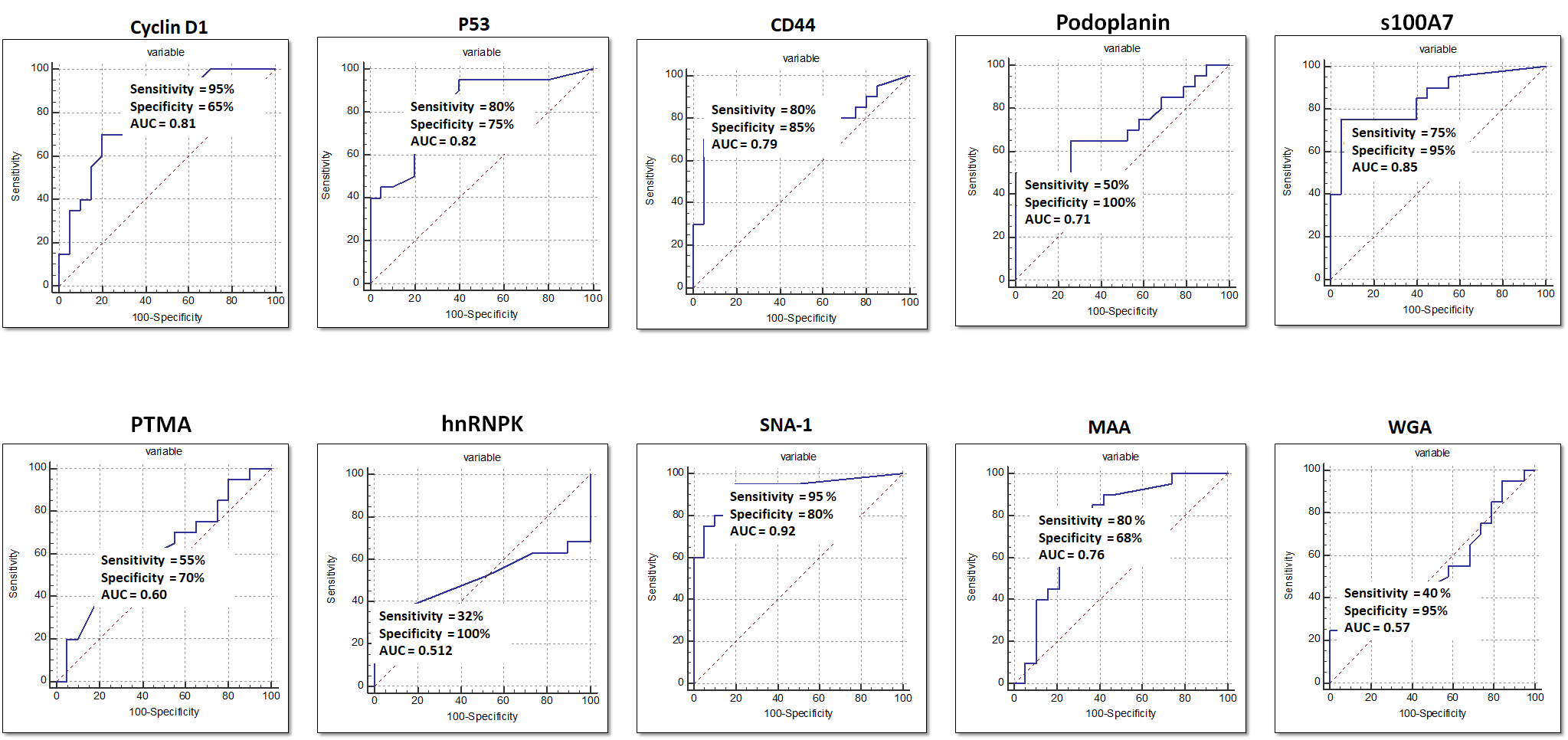

Supplement: S9 Fig — Receiver operating characteristic (ROC) curves of markers (n = 10) in differentiating Low-Risk Lesions (LRL) from High Grade Dysplasia (HGD), and Oral Squamous Cell Carcinoma (OSCC). SNA-1 (AUC = 0.92) and S100A7 (AUC = 0.85) had the highest Area Under Curve, which significantly differentiate LRL (n = 20) from HRL (HGD+OSCC; n = 20). (TIF) [file pone.0291972.s009.tif]

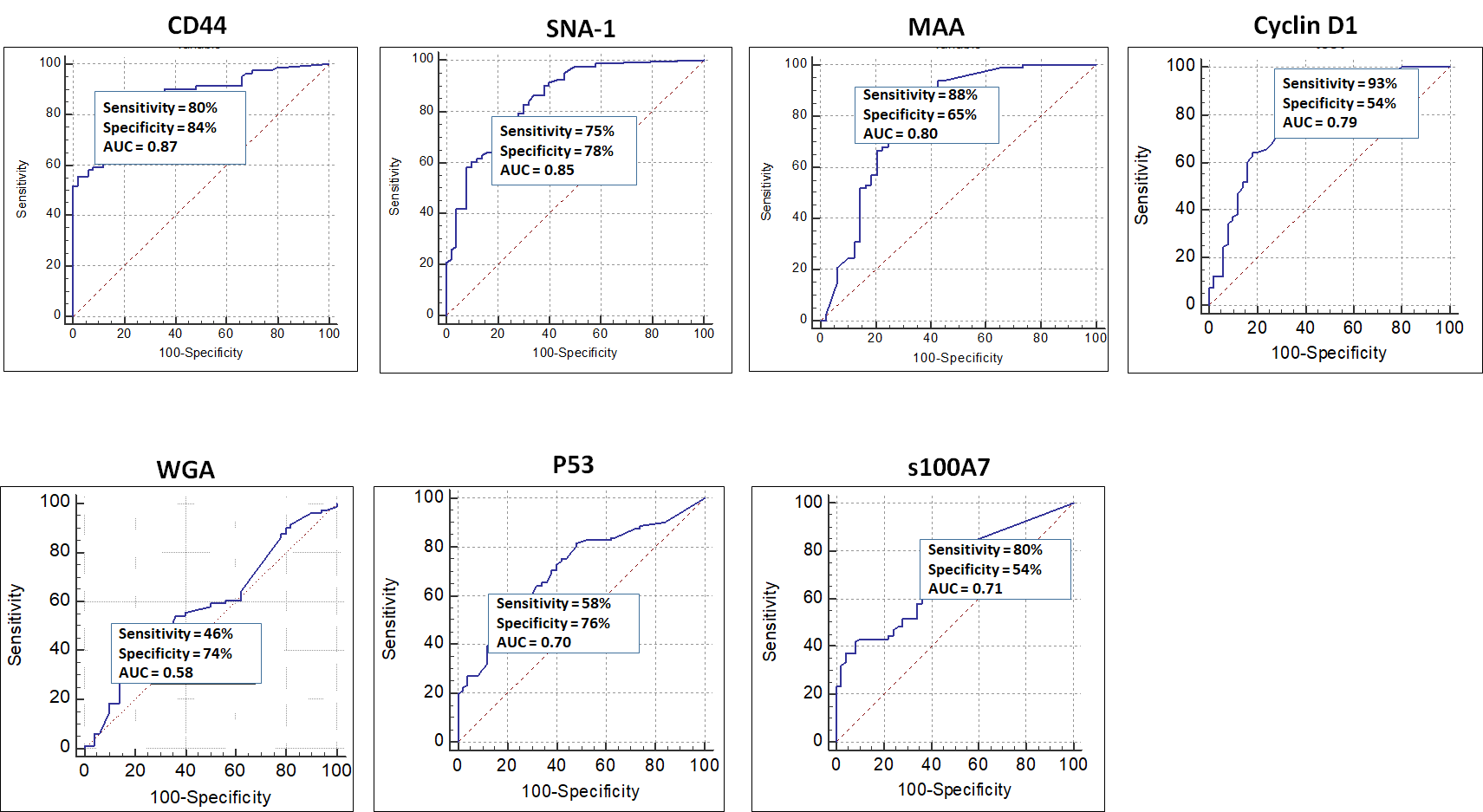

Supplement: S10 Fig — Receiver operating characteristic (ROC) curves of markers (n = 7) in differentiating Low-Risk Lesions (LRL) from High-Grade Dysplasia (HGD), and Oral Squamous Cell Carcinoma (OSCC). SNA-1 (AUC = 0.85), CD44 (AUC = 0.87), MAA (AUC = 0.80) and CyclinD1 (AUC = 0.79) highest Area Under Curve (AUC), which significantly differentiate LRL (n = 50) from HRL (HGD+OSCC; n = 81). (TIF) [file pone.0291972.s010.tif]

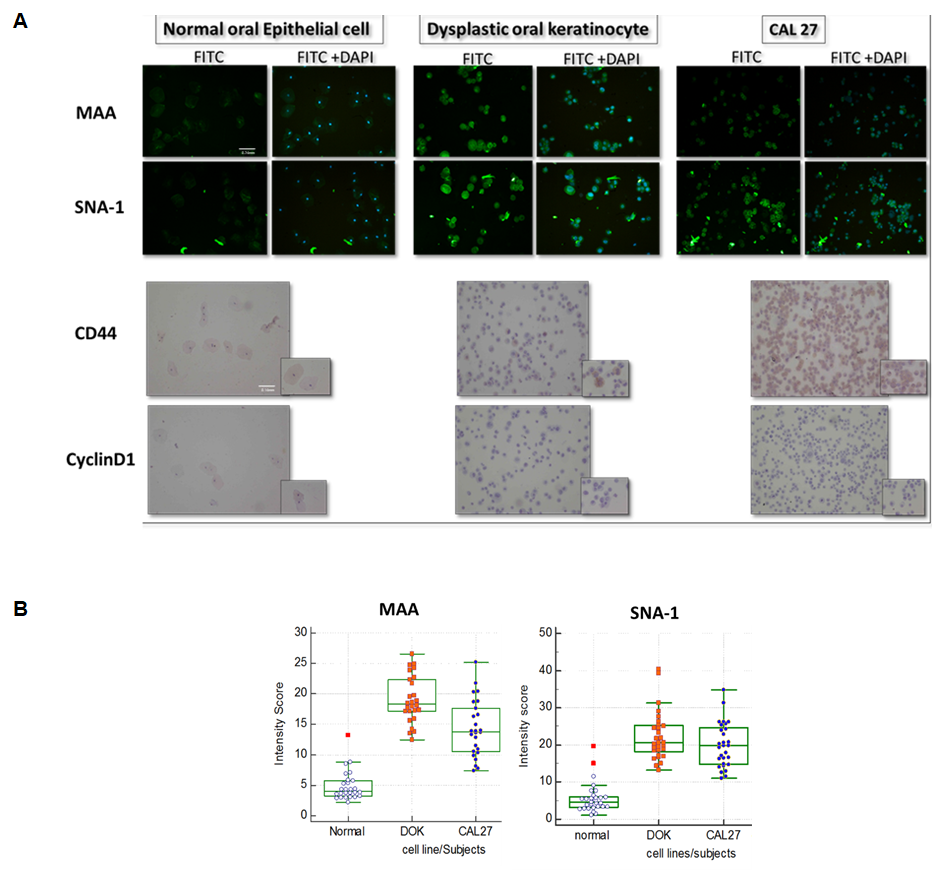

Supplement: S11 Fig — Fluorescent Images (A) showing MAA and SNA-1 staining of cultured cell lines (CAL 27/DOK) and oral epithelial cells from normal subject. Images being taken with fluorescent microscope (Magnification: 200x, scale bar = 0.09mm). Immunocytology images showing the CD44 and CyclinD1 staining of CAL27/DOK and normal epithelial cells (magnification: 100X; scale bar = 0.14mm, Inset: 400X). Box and whisker plot showing the intensity differences of cells in the cell lines in comparison with the buccal cells from normal subject of MAA and SNA-1. CAL 27, DOK shows significantly high staining of markers compared to normal oral epithelial cells (p<0.0001). (TIF) [file pone.0291972.s011.tif]

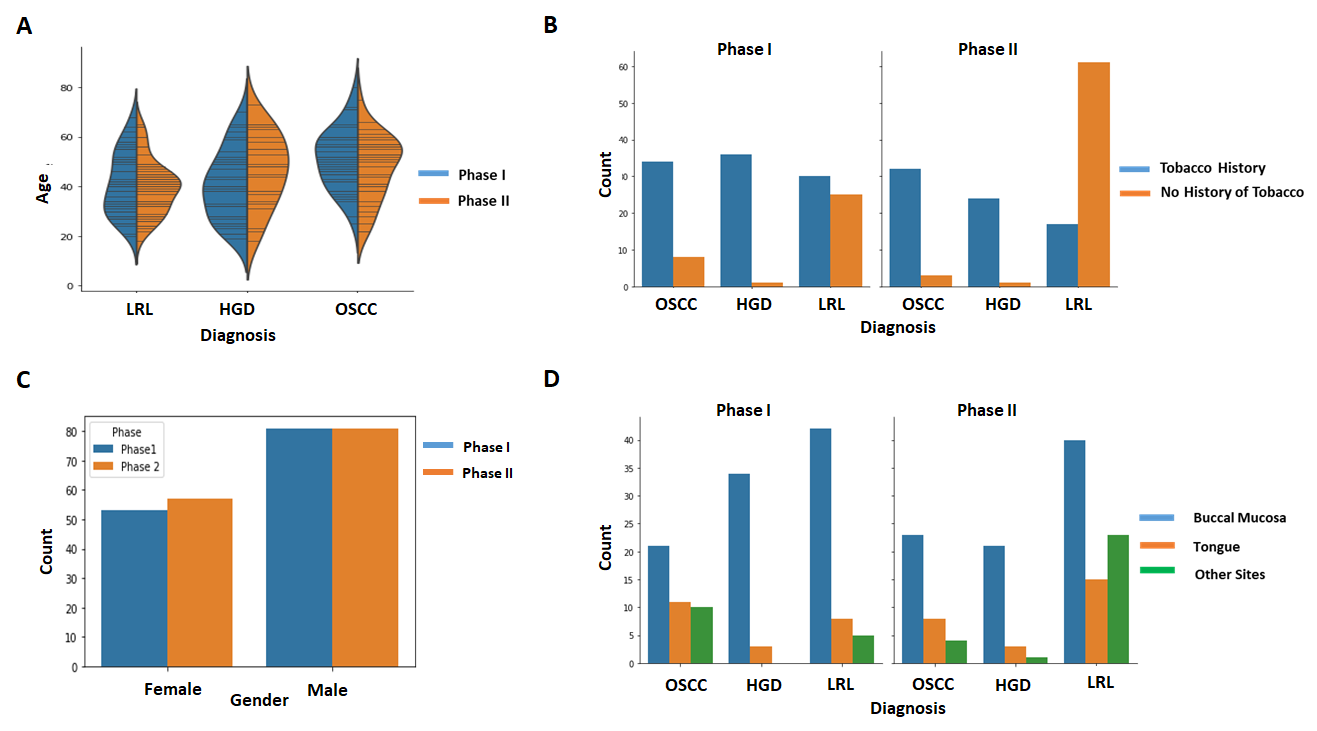

Supplement: S12 Fig — Age distribution (A) of patients in the two validation phases does not show a significant difference, however, patients in both phases showed a high median age. Patients with tobacco chewing/smoking history were higher in OSCC/HGD cohort (B) and tobacco history was less noticed in phase II of LRL cohort. Gender distribution depicts (C) that the male-female ratio was high in both phases. The majority of the oral lesions (D) were present in buccal mucosa in both the phases of ICC. LRL: Non-Dysplastic oral lesion. HGD: Moderate/Severe Dysplasia, OSCC: Oral Squamous cell carcinoma. (TIF) [file pone.0291972.s012.tif]

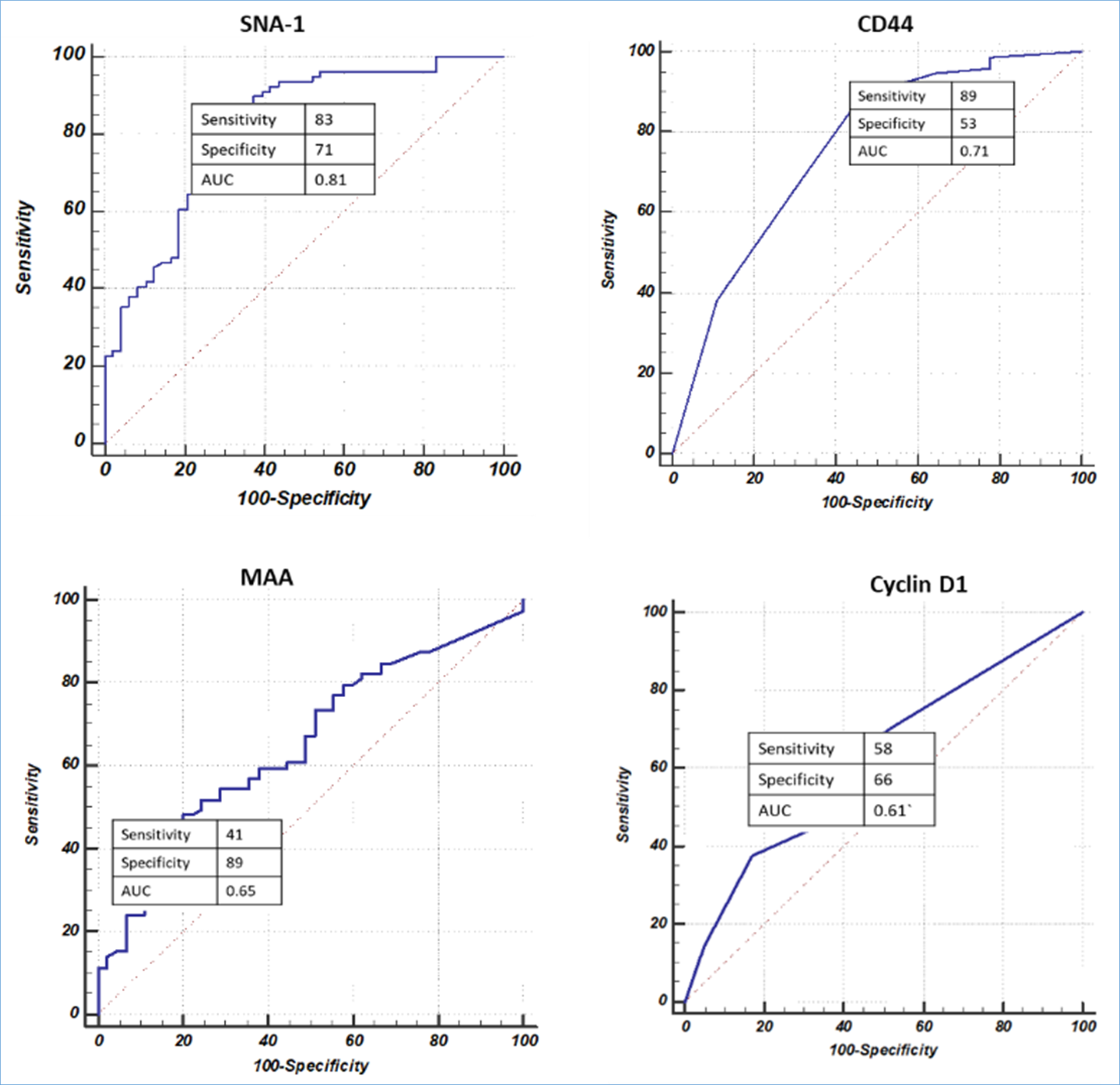

Supplement: S13 Fig — The individual marker features (SNA-1/MAA: mean intensity, maximum intensity and percentage of cells; CD44/Cyclin D1: N+, maximum intensity and percentage of cells with high intensity) were evaluated for single marker efficacy by logistic regression analysis. The features showed higher Receiver Operating-Area Under Curve (AUC) for SNA-1 (0.81) and CD44 (0.71), with high sensitivity (>80%). (TIF) [file pone.0291972.s013.tif]

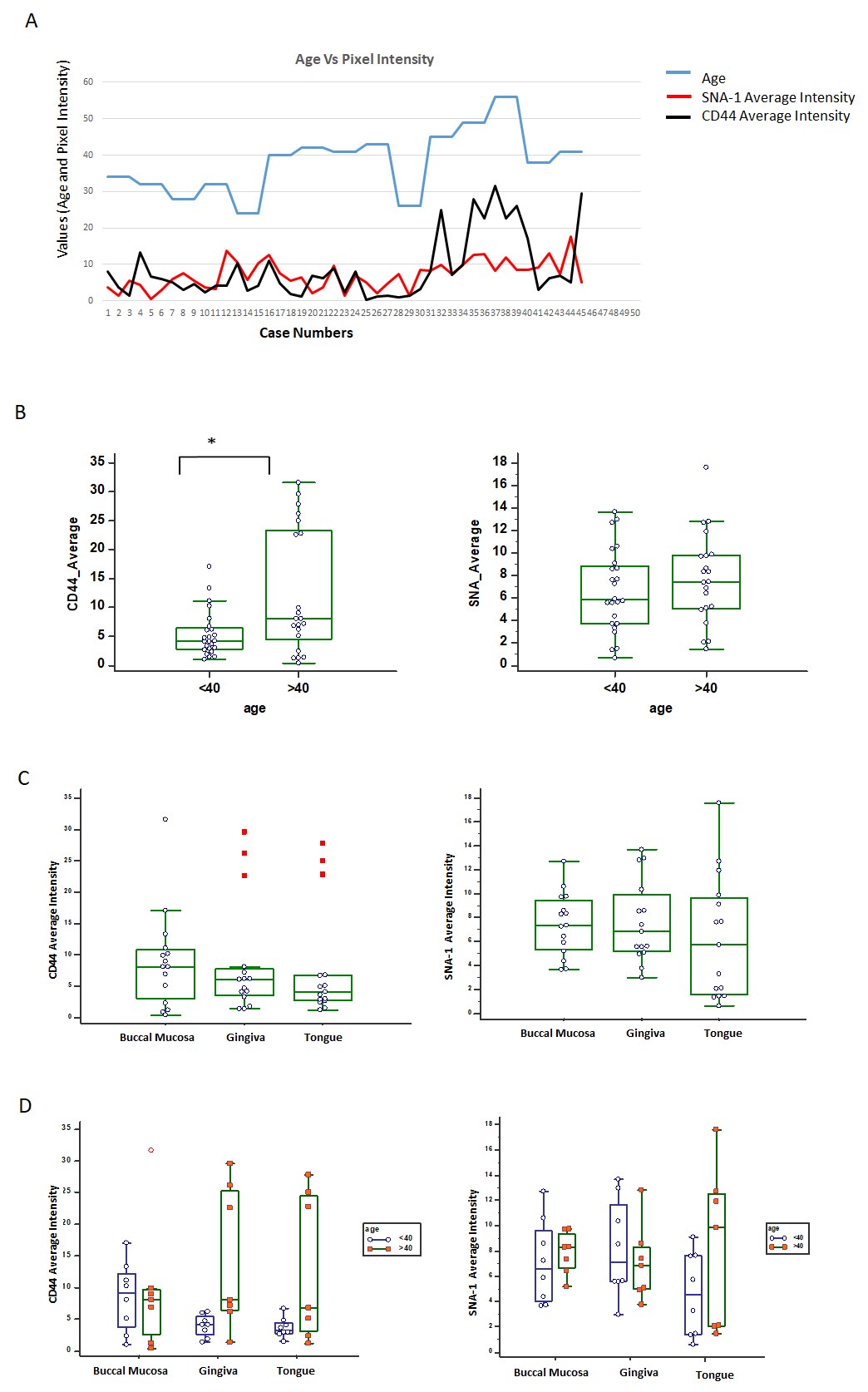

Supplement: S14 Fig — Nomogram of markers (Phase II ICC): Multiplexed immuno-cytological analysis of baseline intensity carried out in the cells of healthy volunteers (n = 15; sites: 45) with regard to age-specific (A, B) and site (C) changes, showed no-significant difference in SNA-1 staining (A-C). CD44, however, showed high expression in tongue and gingiva sites in the elderly age group (>40 years; B, D). (TIF) [file pone.0291972.s014.tif]

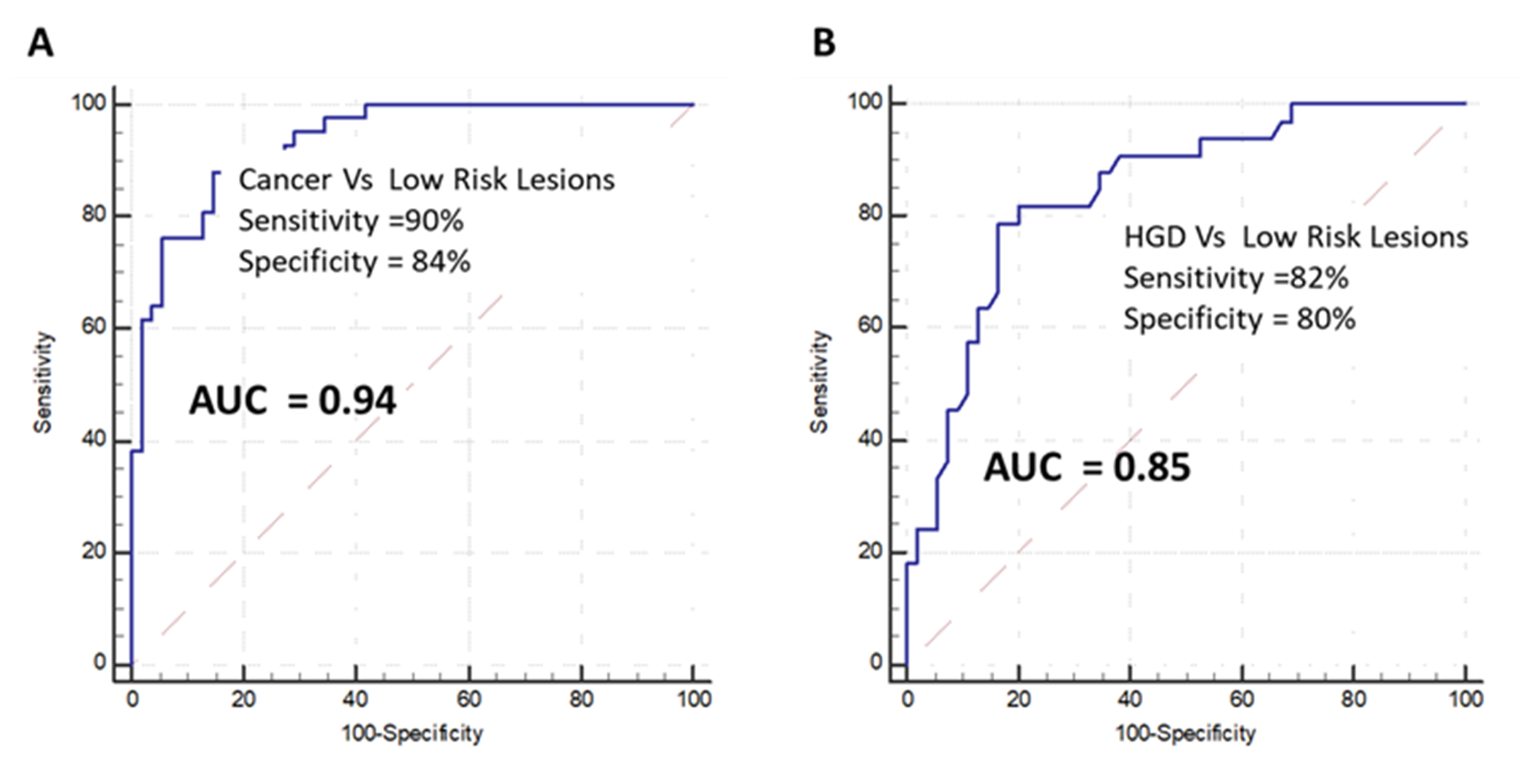

Supplement: S15 Fig — Combination of marker features (CD44 and SNA-1) (logistic regression analysis) differentiate cancer from Low Risk Lesions (LRL) with a sensitivity of 90% (A; AUC: 0.94). High Grade Dysplasia (HGD) was differentiated from LRL with a sensitivity and specificity >80% (B; AUC: 0.85). (TIF) [file pone.0291972.s015.tif]

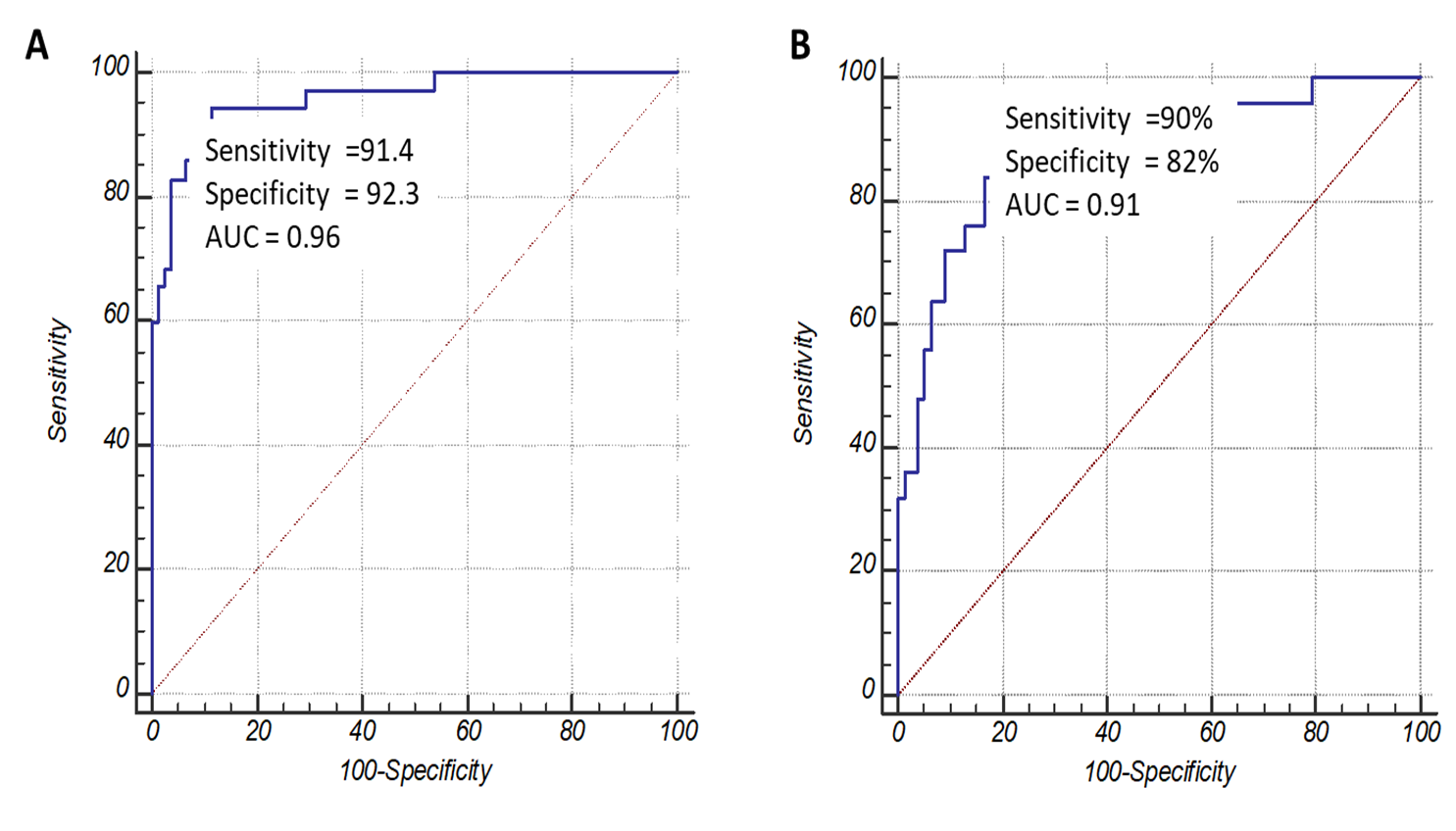

Supplement: S16 Fig — Multiplex validation of marker features (CD44 and SNA-1; logistic regression analysis) differentiated Cancer from Low Risk Lesions (LRL) with a sensitivity and specificity greater than 90% (A; AUC: 0.96). High Grade Dysplasia (HGD) cohort was also differentiated from LRL with a sensitivity of 90% (B; AUC: 0.91). (TIF) [file pone.0291972.s016.tif]

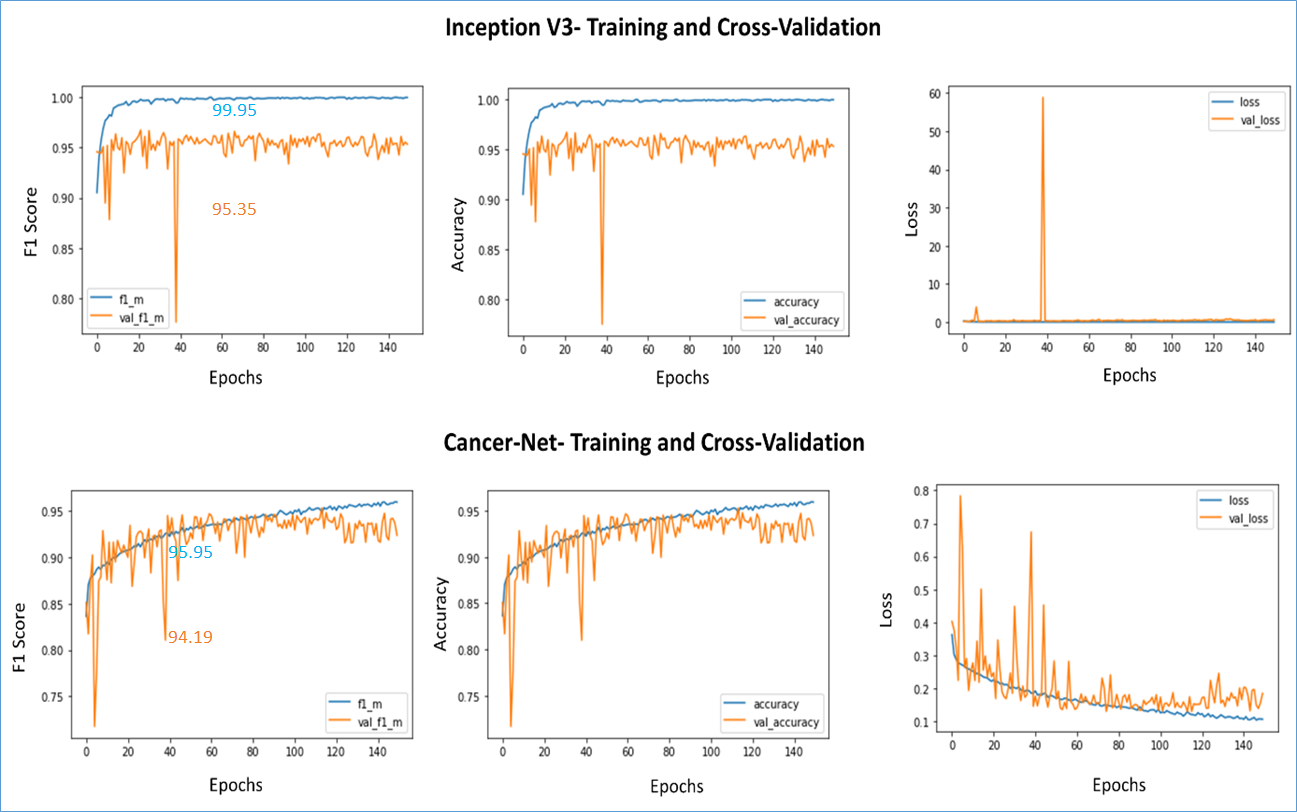

Supplement: S17 Fig — Graph depicting training and cross-validation F1 Score, accuracy and total loss of Inception V3 and Cancer Net model. (TIF) [file pone.0291972.s017.tif]

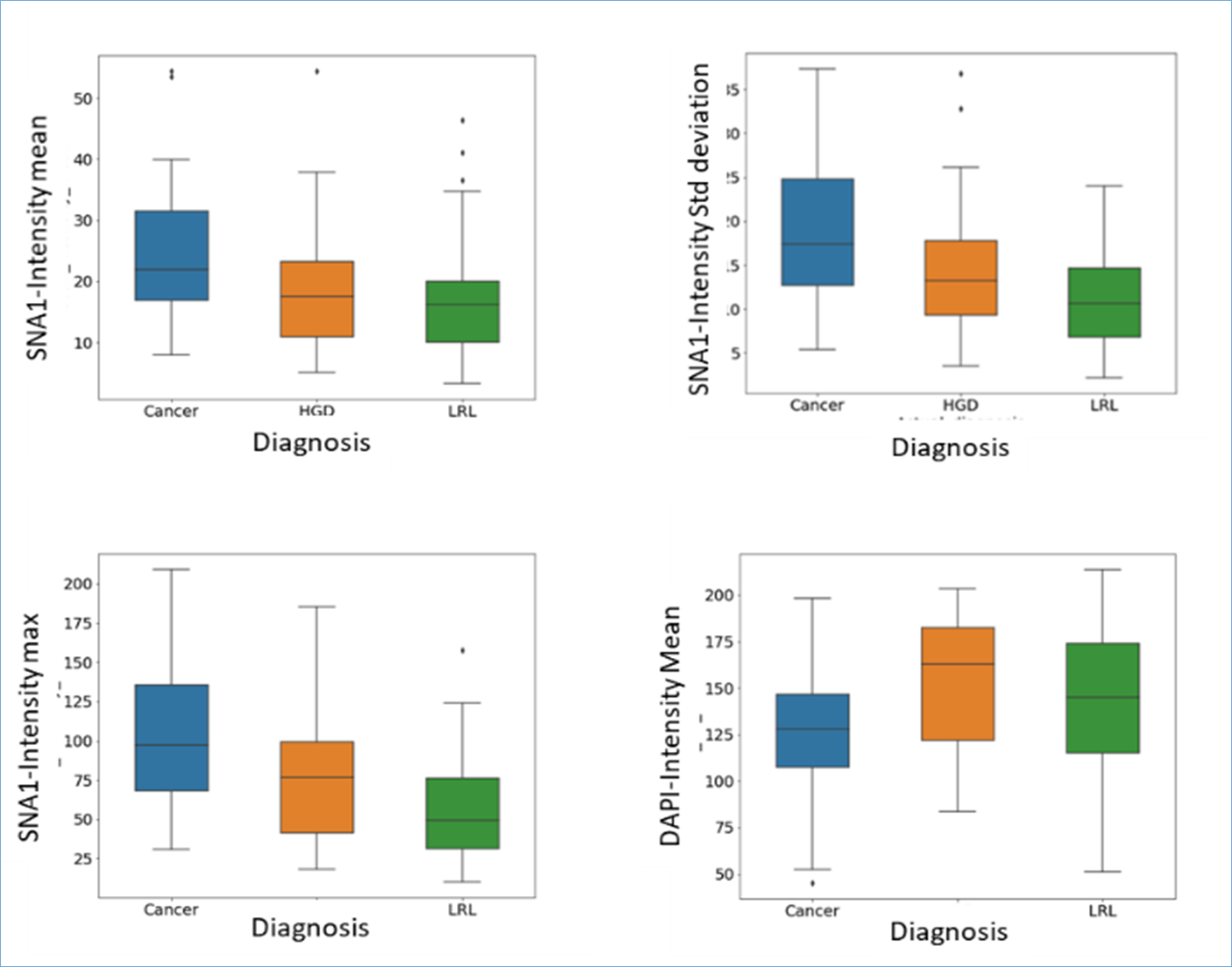

Supplement: S18 Fig — Graph depicting the intensity scores of SNA-1 and DAPI markers in LRL, HGD, and cancer evaluated after cell segmentation (SNA-1 data set, Phase I ICC). (TIF) [file pone.0291972.s018.tif]

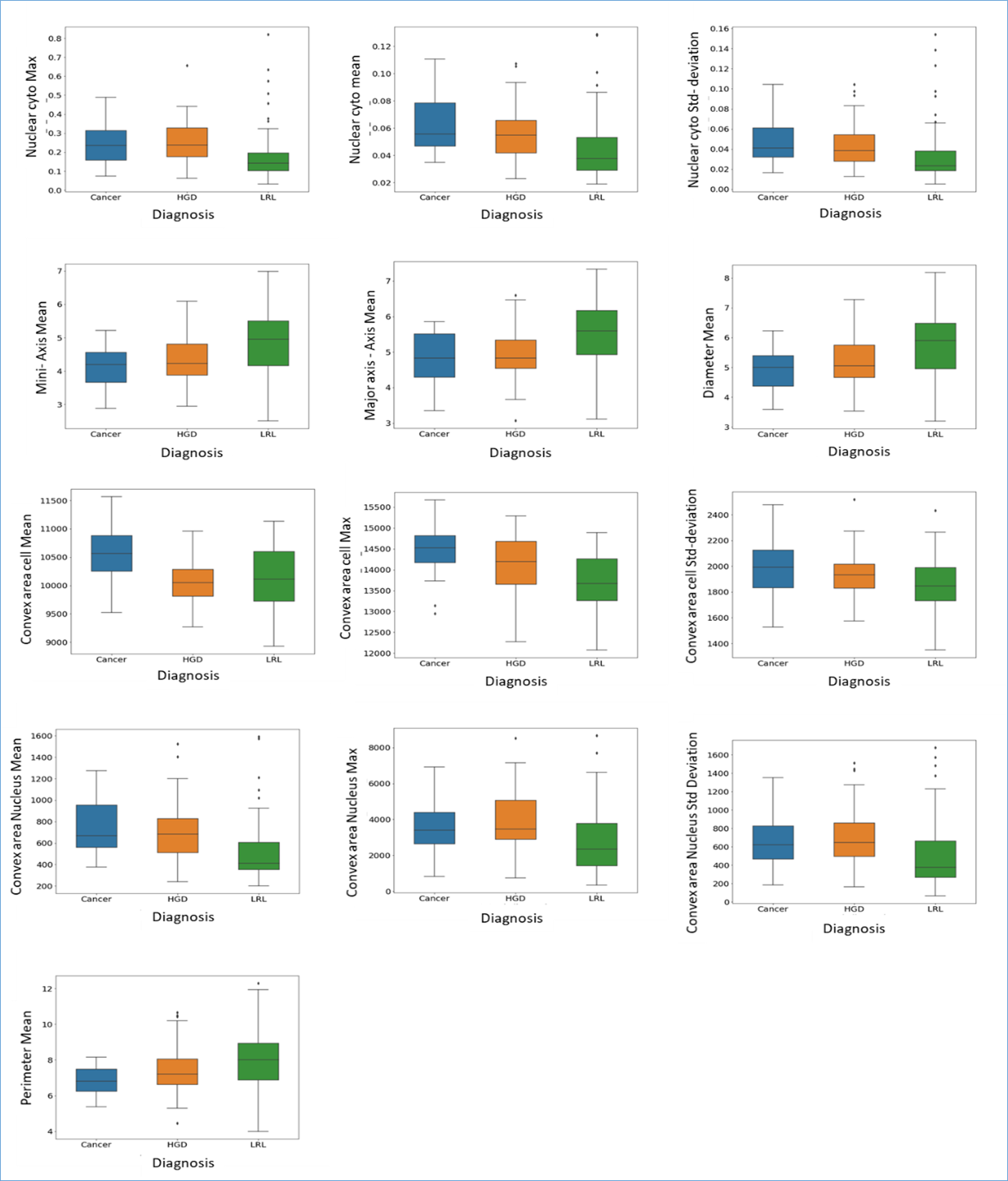

Supplement: S19 Fig — Graph depicting the different cellular and nuclear measurements and their ratios in LRL, HGD and cancer from SNA-1 stained single epithelial cells evaluated after cell segmentation (SNA-1 data set, Phase I ICC). (TIF) [file pone.0291972.s019.tif]

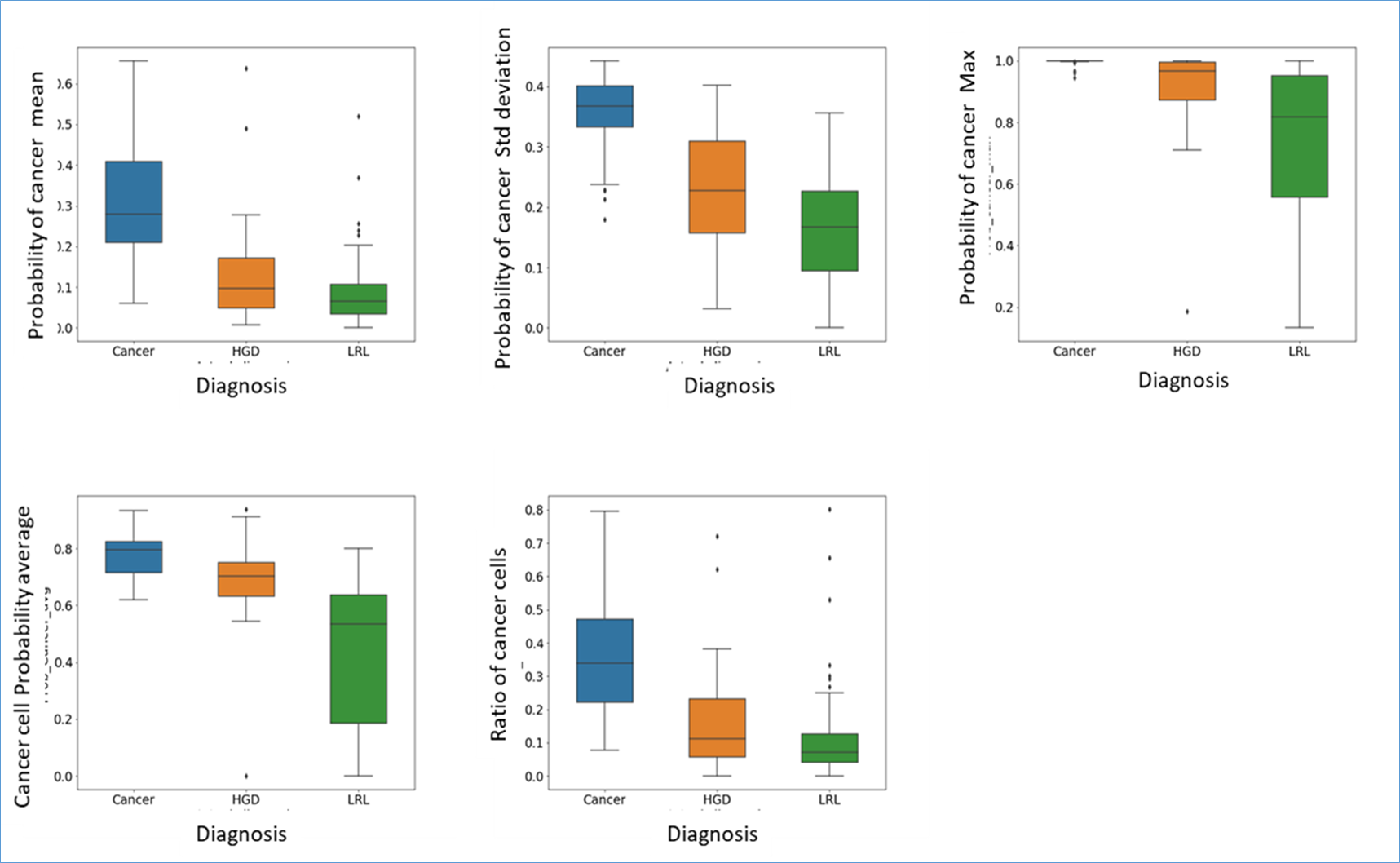

Supplement: S20 Fig — The graph depicting the average, standard deviation, maximum and ratio of atypical cells values of Cancer Net Model (SNA-1 data set, Phase I ICC). (TIF) [file pone.0291972.s020.tif]
