# Supplementary material for: CD44-SNA1 integrated cytopathology for delineation of high grade dysplastic and neoplastic oral lesions
Source: PLoS One. 2023 Sep 25;18(9):e0291972. doi: 10.1371/journal.pone.0291972 (PMC10519609; doi:10.1371/journal.pone.0291972)
Supplement: S1 Table — Data included author details, year of publication, marker, Pubmed ID and case number both in Non-Dysplastic Oral Lesions (ND-OL) and Dysplastic Oral Potentially Malignant Disorders (D-OPMD). (DOCX) [file pone.0291972.s022.docx]

| **No** | ***Author year*** | ***Marker*** | ***PubMed Id*** | ***staining*** | ***ND-OL*** | ***D-OPMD*** | ***True Positive*** | ***False Positive*** | ***False Negative*** | ***True Negative*** | ***ND-OL*** | | ***D-OPMD*** |  |
| --- | --- | --- | --- | --- | --- | --- | --- | --- | --- | --- | --- | --- | --- | --- |
|  |  |  |  |  |  |  |  |  |  |  | ***Mean*** | ***Std Dev*** | ***Mean*** | ***Std Dev*** |
| *1* | Abrahao 2011[1] | P53 | 21359449 | nuclear | 5 | 18 | 7 | 0 | 11 | 5 |  |  |  |  |
|  | Abrahao 2011[1] | hTERT | 21359449 | nuclear | 5 | 18 | 15 | 5 | 3 | 0 |  |  |  |  |
|  | Abrahao 2011[1] | P16 | 21359449 | nuclear, Cytoplasmic | 5 | 18 | 4 | 2 | 14 | 3 |  |  |  |  |
| *2* | Agarwal 1999[2] | P53 | 10435158 | nuclear, Cytoplasmic | 53 | 10 | 7 | 17 | 3 | 36 | 0.53 | 0.92 | 1.3 | 1.15 |
|  | Agarwal 1999[2] | MDM2 | 10435158 | nuclear, cytoplasmic | 53 | 10 | 6 | 13 | 4 | 40 | 0.46 | 0.92 | 1 | 1.05 |
| 3 | Gonçalves 2017[3] | P53 | 28941745 | nucleus | 33 | 67 | 54 | 9 | 13 | 24 |  |  |  |  |
| *4* | Bradley 2006[4] | p16 | 16799478 | nucleus, cytoplasm | 33 | 86 | 10 | 3 | 76 | 30 |  |  |  |  |
| *5* | Mack 2008[5] | CD44s | 18852874 | cell membrane | 10 | 22 | 0 | 0 | 22 | 10 | 249.5 | 45.85 | 276.5 | 36.25 |
|  | Mack 2008[5] | CD44V6 | 18852874 | cell membrane | 10 | 22 | 0 | 0 | 22 | 10 | 198 | 35.73 | 239.6 | 53.91 |
| *6* | Buajeeb 2009[6] | p16 | 19192055 | nuclear/ cytoplasmic | 25 | 15 | 0 | 4 | 15 | 21 |  |  |  |  |
| *7* | Chandrashekhar 2015[7] | PCNA | 26266215 | Nuclear | 10 | 10 |  |  |  |  | 160.6 | 41.73 | 229 | 49.39 |
| *8* | chen 1999[8] | P16 | 10235368 | nuclear, cytoplasm | 20 | 30 | 14 | 3 | 16 | 17 |  |  |  |  |
|  | chen 1999[8] | CDK4 | 10235368 | nuclear | 20 | 30 | 14 | 4 | 16 | 16 |  |  |  |  |
| *9* | Chiang 2000[9] | PCNA | 10899674 | nuclear | 20 | 10 | 10 | 11 | 0 | 9 | 17.16 | 3.96 | 44.9 | 15.4 |
| *10* | Coltrera 1992[10] | PCNA | 1384338 | nuclear | 80 | 16 | 16 | 0 | 0 | 80 |  |  |  |  |
|  | Coltrera 1992[10] | P53 | 1384338 | Nuclear | 80 | 16 | 3 | 0 | 13 | 80 |  |  |  |  |
| *11* | Cruz 1998[11] | P53 | 9664901 | nucleus | 17 | 35 | 7 | 0 | 28 | 17 |  |  |  |  |
| *12* | D’souza 2016[12] | Podoplanin | 27153448 | cytoplasm | 24 | 20 | 19 | 14 | 1 | 10 |  |  |  |  |
| *13* | Deepa 2017[13] | podoplanin | 29410757 | Cytoplasm | 20 | 20 | 13 | 7 | 7 | 13 |  |  |  |  |
| 14 | De Freitas Silva, 2013[14] | Ecadherin | 23772858 | cytoplasm | 10 | 30 | 19 | 10 | 11 | 0 |  |  |  |  |
| *15* | Funayama 2010[15] | Podoplanin | 21613804 | cytoplasm | 60 | 208 | 68 | 0 | 140 | 60 |  |  |  |  |
| *16* | Gadbail 2017[16] | Ki67 | 28672080 | nuclei | 56 | 115 |  |  |  |  | 12.47 | 2.34 | 34.78 | 9.74 |
| *17* | Girod 1998[17] | P53 | 9762452 | nucleus | 79 | 34 | 13 | 23 | 21 | 56 |  |  |  |  |
|  | Girod 1998[17] | MDM2 | 9762452 | nucleus | 79 | 34 | 15 | 25 | 19 | 54 |  |  |  |  |
| *18* | Huang 1994[18] | PCNA | 7534896 | Nuclear | 46 | 98 | 89 | 36 | 9 | 10 |  |  |  |  |
| *19* | Iwasa 2009[19] | PCNA | 7534896 | nuclear | 45 | 31 |  |  |  |  | 19.4 | 6.6 | 44.6 | 16 |
|  | Iwasa 2009[19] | P53 | 7534896 | nuclear | 45 | 31 | 13 | 9 | 18 | 36 |  |  |  |  |
| 20 | Jatindra Kaur 2013[20] | ECadherin | 23840677 | membrane | 86 | 20 | 8 | 62 | 12 | 24 |  |  |  |  |
| *21* | Palani 2011[21] | hTERT | 21891916 | nuclear | 10 | 15 | 7 | 4 | 8 | 6 | 64.9 | 30.7 | 85.6 | 25.1 |
| *22* | Jordan 1998[22] | p27 | 9466585 | nuclear | 10 | 36 | 0 | 0 | 36 | 10 | 49.6 | 5.8 | 31.4 | 4 |
|  | Jordan 1998[22] | Ki67 | 9466585 | nuclear | 10 | 36 |  |  |  |  | 16.3 | 3.3 | 30.2 | 4.5 |
| *23* | Kawaguchi 2008[23] | podoplanin | 18202409 | cell membrane | 101 | 49 | 24 | 32 | 25 | 69 |  |  |  |  |
| *24* | Kerdpon 1997[24] | P53 | 9467348 | nucleus | 58 | 41 | 35 | 18 | 6 | 40 | 32 | 7 | 41 | 18 |
| 25 | Kodani 2001[25] | Ki67 | 11872961 | Nuclear | 12 | 69 |  |  |  |  | 9.6 | 3.464 | 24 | 11.629 |
|  | Kodani 2001[25] | P53 | 11872961 | Nuclear | 12 | 69 | 0 | 0 | 69 | 12 | 0.6 | 1.385 | 16 | 14.952 |
|  | Kodani 2001[25] | P27 | 11872961 | Nuclear | 12 | 69 | 0 | 0 | 69 | 12 | 24.4 | 6.928 | 19.8 | 12.46 |
|  | Kodani 2001[25] | P21 | 11872961 | Nuclear | 12 | 69 |  |  |  |  | 24.7 | 6.581 | 14.6 | 9.1373 |
| *26* | Kreppel, 2012[26] | Podoplanin | 22471854 | cytoplasm | 31 | 29 | 18 | 6 | 11 | 25 |  |  |  |  |
| *27* | Kurokawa 2003[27] | syndecan-1 | 12969225 | membrane | 35 | 30 | 12 | 35 | 18 | 0 |  |  |  |  |
| *28* | Lakkam 2014[28] | syndecan-1 | 24762495 | Membrane | 10 | 30 | 11 | 10 | 19 | 0 |  |  |  |  |
| *29* | Basnaker 2014[29] | CyclinD1 | 24995250 | nuclear | 19 | 13 | 10 | 8 | 3 | 11 |  |  |  |  |
| *30* | Martinez 2012[30] | CyclinD1 | 22783442 | nuclear | 34 | 11 | 6 | 4 | 5 | 30 |  |  |  |  |
|  | Martinez 2012[30] | P53 | 22783442 | nuclear | 34 | 11 | 5 | 10 | 6 | 24 |  |  |  |  |
|  | Martinez 2012[30] | MDM2 | 22783442 | nuclear | 34 | 11 | 0 | 5 | 11 | 29 |  |  |  |  |
|  | Martinez 2012[30] | Ki67 | 22783442 | nuclear | 34 | 11 | 7 | 11 | 4 | 23 |  |  |  |  |
| 31 | Pigatti 2014[31] | Ki67 | 25265990 | Nuclear | 23 | 14 | 13 | 13 | 1 | 10 |  |  |  |  |
| *32* | Poomsawat 2010[32] | CDK4 | 20618617 | Nuclear | 32 | 14 | 13 | 16 | 1 | 16 |  |  |  |  |
| *33* | Raghunathan 2016 [33] | hTERT | 27194869 | nuclear | 10 | 21 | 13 | 0 | 8 | 10 |  |  |  |  |
| *34* | Raju 2005[34] | p53 | 16334163 | nuclear | 4 | 25 | 19 | 0 | 6 | 4 |  |  |  |  |
|  | Raju 2005[34] | CyclinD1 | 16334163 | nuclear | 4 | 25 | 24 | 0 | 1 | 4 |  |  |  |  |
|  | Raju 2005[34] | Ki67 | 16334163 | nuclear | 4 | 23 | 12 | 1 | 11 | 3 |  |  |  |  |
| 35 | Ralhan 2005[35] | P53 | 16161051 | Not mentioned | 94 | 37 | 18 | 20 | 19 | 74 |  |  |  |  |
|  | Ralhan 2005[35] | p21 | 16161051 | Not mentioned | 94 | 37 | 26 | 36 | 11 | 58 |  |  |  |  |
|  | Ralhan 2005[35] | p16 | 16161051 | Not mentioned | 94 | 37 | 22 | 35 | 15 | 59 |  |  |  |  |
| *36* | Rich 1999[36] | P53 | 10452165 | Nuclear | 58 | 41 | 35 | 18 | 6 | 40 |  |  |  |  |
| *37* | Saito 1999[37] | p53 | 10226946 | nuclear | 10 | 57 | 0 | 0 | 57 | 10 | 5.1 | 1.62 | 16.52 | 9.28 |
|  | Saito 1999[37] | p16 | 10226946 | nuclear | 10 | 57 |  |  |  |  | 2 | 1.62 | 4.363 | 4.153 |
|  | Saito 1999[37] | Ki67 | 10226946 | nuclear | 10 | 57 |  |  |  |  | 14.9 | 4.28 | 36.97 | 8.25 |
|  | Saito 1999[37] | p27 | 10226946 | nuclear | 10 | 57 | 0 | 0 | 57 | 10 | 53 | 4.28 | 33.73 | 9.31 |
| *38* | Viveka 2016[38] | p53 | 26838208 | nucleus | 8 | 21 | 11 | 2 | 10 | 6 |  |  |  |  |
| *39* | Varun 2012[39] | p53 | 23776093 | nucleus | 10 | 20 | 0 | 0 | 20 | 10 | 15.1 | 9 | 37.6 | 12.6 |
| *40* | Turatti 2005[40] | CyclinD1 | 16050486 | nucleus | 15 | 41 | 2 | 0 | 39 | 15 |  |  |  |  |
| *41* | Kamat 2013[41] | syndecan-1 | 24019799 | cell membrane, cytoplasm | 35 | 45 | 33 | 35 | 12 | 0 |  |  |  |  |
| *42* | Subin 2017[42] | CD44 | 29224811 | Cell membrane | 233 | 229 | 0 | 0 | 229 | 233 | 73.15 | 5.32 | 97.24 | 4.04 |
| *43* | Sridevi 2015[43] | Ecadherin | 26430364 | membrane | 20 | 10 | 6 | 14 | 4 | 6 |  |  |  |  |
| *44* | Schoelch 1999[44] | p53 | 10621856 | nuclear | 7 | 48 | 15 | 0 | 33 | 7 |  |  |  |  |
|  | Schoelch 1999[44] | CyclinD1 | 10621856 | nuclear | 7 | 48 | 8 | 1 | 40 | 6 |  |  |  |  |
|  | Schoelch 1999[44] | Ki67 | 10621856 | nuclear | 7 | 48 | 44 | 4 | 4 | 3 |  |  |  |  |
|  | Schoelch 1999[44] | p21 | 10621856 | nuclear | 7 | 48 | 46 | 6 | 2 | 1 |  |  |  |  |
|  | Schoelch 1999[44] | p27 | 10621856 | nuclear | 7 | 48 | 33 | 4 | 15 | 3 |  |  |  |  |
| *45* | Sinanoglu 2015[45] | Ki67 | 26229582 | nuclear | 19 | 11 |  |  |  |  | 36.01 | 4.14 | 39.49 | 6.94 |
| *46* | Shintani 2002[46] | CyclinD1 | 11978545 | nuclear | 20 | 42 | 2 | 0 | 40 | 20 |  |  |  |  |
|  | Shintani 2002[46] | CDK4 | 11978545 | nuclear | 20 | 42 | 12 | 0 | 30 | 20 |  |  |  |  |
|  | Shintani 2002[46] | P16 | 11978545 | nuclear | 20 | 42 | 37 | 20 | 5 | 0 |  |  |  |  |
|  | Shintani 2002[46] | P21 | 11978545 | nuclear | 20 | 42 | 24 | 20 | 18 | 0 |  |  |  |  |
|  | Shintani 2002[46] | P27 | 11978545 | nuclear | 20 | 42 | 37 | 20 | 5 | 0 |  |  |  |  |
| **S1 Table. Data extracted from articles for meta-analysis**. Data included author details, year of publication, marker, Pubmed ID and case number both in Non-Dysplastic Oral Lesions (ND-OL) and Dysplastic Oral Potentially Malignant Disorders (D-OPMD)**.** | | | | | | | | | | | | | | |

1. Abrahao AC, Bonelli BV, Nunes FD, Dias EP, Cabral MG. Immunohistochemical expression of p53, p16 and hTERT in oral squamous cell carcinoma and potentially malignant disorders. Braz Oral Res. 2011;25(1):34-41. Epub 2011/03/02. doi: 10.1590/s1806-83242011000100007. PubMed PMID: 21359449.

2. Agarwal S, Mathur M, Srivastava A, Ralhan R. MDM2/p53 co-expression in oral premalignant and malignant lesions: potential prognostic implications. Oral oncology. 1999;35(2):209-16. Epub 1999/08/06. doi: 10.1016/s1368-8375(98)00092-x. PubMed PMID: 10435158.

3. Goncalves AS, Mosconi C, Jaeger F, Wastowski IJ, Aguiar MCF, Silva TA, et al. Overexpression of immunomodulatory mediators in oral precancerous lesions. Human immunology. 2017;78(11-12):752-7. doi: 10.1016/j.humimm.2017.09.003. PubMed PMID: 28941745.

4. Bradley KT, Budnick SD, Logani S. Immunohistochemical detection of p16INK4a in dysplastic lesions of the oral cavity. Mod Pathol. 2006;19(10):1310-6. Epub 2006/06/27. doi: 10.1038/modpathol.3800649. PubMed PMID: 16799478.

5. Mack B, Gires O. CD44s and CD44v6 expression in head and neck epithelia. PloS one. 2008;3(10):e3360. doi: 10.1371/journal.pone.0003360. PubMed PMID: 18852874; PubMed Central PMCID: PMC2566597.

6. Buajeeb W, Poomsawat S, Punyasingh J, Sanguansin S. Expression of p16 in oral cancer and premalignant lesions. Journal of oral pathology & medicine : official publication of the International Association of Oral Pathologists and the American Academy of Oral Pathology. 2009;38(1):104-8. doi: 10.1111/j.1600-0714.2008.00710.x. PubMed PMID: 19192055.

7. Poosarla C, Ramesh M, Ramesh K, Gudiseva S, Bala S, Sundar M. Proliferating Cell Nuclear Antigen in Premalignancy and Oral Squamous Cell Carcinoma. Journal of clinical and diagnostic research : JCDR. 2015;9(6):ZC39-41. Epub 2015/08/13. doi: 10.7860/JCDR/2015/12645.6094. PubMed PMID: 26266215; PubMed Central PMCID: PMCPMC4525605.

8. Chen Q, Luo G, Li B, Samaranayake LP. Expression of p16 and CDK4 in oral premalignant lesions and oral squamous cell carcinomas: a semi-quantitative immunohistochemical study. Journal of oral pathology & medicine : official publication of the International Association of Oral Pathologists and the American Academy of Oral Pathology. 1999;28(4):158-64. PubMed PMID: 10235368.

9. Chiang CP, Lang MJ, Liu BY, Wang JT, Leu JS, Hahn LJ, et al. Expression of proliferating cell nuclear antigen (PCNA) in oral submucous fibrosis, oral epithelial hyperkeratosis and oral epithelial dysplasia in Taiwan. Oral oncology. 2000;36(4):353-9. PubMed PMID: 10899674.

10. Coltrera MD, Zarbo RJ, Sakr WA, Gown AM. Markers for dysplasia of the upper aerodigestive tract. Suprabasal expression of PCNA, p53, and CK19 in alcohol-fixed, embedded tissue. The American journal of pathology. 1992;141(4):817-25. PubMed PMID: 1384338; PubMed Central PMCID: PMC1886630.

11. Cruz IB, Snijders PJ, Meijer CJ, Braakhuis BJ, Snow GB, Walboomers JM, et al. p53 expression above the basal cell layer in oral mucosa is an early event of malignant transformation and has predictive value for developing oral squamous cell carcinoma. The Journal of pathology. 1998;184(4):360-8. doi: 10.1002/(SICI)1096-9896(199804)184:4<360::AID-PATH1263>3.0.CO;2-H. PubMed PMID: 9664901.

12. D'Souza B, Nayak R, Kotrashetti VS. Immunohistochemical Expression of Podoplanin in Clinical Variants of Oral Leukoplakia and Its Correlation With Epithelial Dysplasia. Applied immunohistochemistry & molecular morphology : AIMM. 2018;26(2):132-9. doi: 10.1097/PAI.0000000000000383. PubMed PMID: 27153448.

13. Fei B, Lu G, Wang X, Zhang H, Little JV, Patel MR, et al. Label-free reflectance hyperspectral imaging for tumor margin assessment: a pilot study on surgical specimens of cancer patients. Journal of biomedical optics. 2017;22(8):1-7. doi: 10.1117/1.JBO.22.8.086009. PubMed PMID: 28849631; PubMed Central PMCID: PMC5572439.

14. de Freitas Silva BS, Yamamoto-Silva FP, Pontes HA, Pinto Junior Ddos S. E-cadherin downregulation and Twist overexpression since early stages of oral carcinogenesis. Journal of oral pathology & medicine : official publication of the International Association of Oral Pathologists and the American Academy of Oral Pathology. 2014;43(2):125-31. doi: 10.1111/jop.12096. PubMed PMID: 23772858.

15. Funayama A, Cheng J, Maruyama S, Yamazaki M, Kobayashi T, Syafriadi M, et al. Enhanced expression of podoplanin in oral carcinomas in situ and squamous cell carcinomas. Pathobiology : journal of immunopathology, molecular and cellular biology. 2011;78(3):171-80. doi: 10.1159/000324926. PubMed PMID: 21613804.

16. Gadbail AR, Chaudhary MS, Sarode SC, Gawande M, Korde S, Tekade SA, et al. Ki67, CD105, and alpha-SMA expressions better relate the binary oral epithelial dysplasia grading system of World Health Organization. Journal of oral pathology & medicine : official publication of the International Association of Oral Pathologists and the American Academy of Oral Pathology. 2017;46(10):921-7. doi: 10.1111/jop.12612. PubMed PMID: 28672080.

17. Girod SC, Pfeiffer P, Ries J, Pape HD. Proliferative activity and loss of function of tumour suppressor genes as 'biomarkers' in diagnosis and prognosis of benign and preneoplastic oral lesions and oral squamous cell carcinoma. The British journal of oral & maxillofacial surgery. 1998;36(4):252-60. PubMed PMID: 9762452.

18. Huang WY, Coltrera M, Schubert M, Morton T, Truelove E. Histopathologic evaluation of proliferating cell nuclear antigen (PC10) in oral epithelial hyperplasias and premalignant lesions. Oral surgery, oral medicine, and oral pathology. 1994;78(6):748-54. Epub 1994/12/01. doi: 10.1016/0030-4220(94)90091-4. PubMed PMID: 7534896.

19. Iwasa M, Imamura Y, Noriki S, Nishi Y, Kato H, Fukuda M. Immunohistochemical detection of early-stage carcinogenesis of oral leukoplakia by increased DNA-instability and various malignancy markers. Eur J Histochem. 2001;45(4):333-46. Epub 2002/02/16. doi: 10.4081/1642. PubMed PMID: 11846001.

20. Kaur J, Sawhney M, DattaGupta S, Shukla NK, Srivastava A, Walfish PG, et al. Clinical significance of altered expression of beta-catenin and E-cadherin in oral dysplasia and cancer: potential link with ALCAM expression. PloS one. 2013;8(6):e67361. doi: 10.1371/journal.pone.0067361. PubMed PMID: 23840677; PubMed Central PMCID: PMC3696121.

21. Palani J, Lakshminarayanan V, Kannan R. Immunohistochemical detection of human telomerase reverse transcriptase in oral cancer and pre-cancer. Indian journal of dental research : official publication of Indian Society for Dental Research. 2011;22(2):362. doi: 10.4103/0970-9290.84281. PubMed PMID: 21891916.

22. Jordan RC, Bradley G, Slingerland J. Reduced levels of the cell-cycle inhibitor p27Kip1 in epithelial dysplasia and carcinoma of the oral cavity. The American journal of pathology. 1998;152(2):585-90. PubMed PMID: 9466585; PubMed Central PMCID: PMC1857966.

23. Kawaguchi H, El-Naggar AK, Papadimitrakopoulou V, Ren H, Fan YH, Feng L, et al. Podoplanin: a novel marker for oral cancer risk in patients with oral premalignancy. Journal of clinical oncology : official journal of the American Society of Clinical Oncology. 2008;26(3):354-60. doi: 10.1200/JCO.2007.13.4072. PubMed PMID: 18202409.

24. Kerdpon D, Rich AM, Reade PC. Expression of p53 in oral mucosal hyperplasia, dysplasia and squamous cell carcinoma. Oral diseases. 1997;3(2):86-92. PubMed PMID: 9467348.

25. Kodani I, Shomori K, Osaki M, Kuratate I, Ryoke K, Ito H. Expression of minichromosome maintenance 2 (MCM2), Ki-67, and cell-cycle-related molecules, and apoptosis in the normal-dysplasia-carcinoma sequence of the oral mucosa. Pathobiology : journal of immunopathology, molecular and cellular biology. 2001;69(3):150-8. doi: 10.1159/000048770. PubMed PMID: 11872961.

26. Kreppel M, Kreppel B, Drebber U, Wedemayer I, Rothamel D, Zoller JE, et al. Podoplanin expression in oral leukoplakia: prognostic value and clinicopathological implications. Oral diseases. 2012;18(7):692-9. doi: 10.1111/j.1601-0825.2012.01927.x. PubMed PMID: 22471854.

27. Kurokawa H, Matsumoto S, Murata T, Yamashita Y, Tomoyose T, Zhang M, et al. Immunohistochemical study of syndecan-1 down-regulation and the expression of p53 protein or Ki-67 antigen in oral leukoplakia with or without epithelial dysplasia. Journal of oral pathology & medicine : official publication of the International Association of Oral Pathologists and the American Academy of Oral Pathology. 2003;32(9):513-21. Epub 2003/09/13. doi: 10.1034/j.1600-0714.2003.00117.x. PubMed PMID: 12969225.

28. Lakkam B, Majage B, Astekar M, Gugwad RS, Giri G, Ramasahayam S. Immunohistochemical expression of syndecan-1 in oral dysplastic epithelium. Journal of cancer research and therapeutics. 2014;10(1):103-6. doi: 10.4103/0973-1482.131407. PubMed PMID: 24762495.

29. Basnaker M, Sp S, Bnvs S. Cyclin d1 gene expression in oral mucosa of tobacco chewers"-an immunohistochemical study. Journal of clinical and diagnostic research : JCDR. 2014;8(5):ZC70-5. doi: 10.7860/JCDR/2014/9456.4406. PubMed PMID: 24995250; PubMed Central PMCID: PMC4080071.

30. Bascones-Martinez A, Lopez-Duran M, Cano-Sanchez J, Sanchez-Verde L, Diez-Rodriguez A, Aguirre-Echebarria P, et al. Differences in the expression of five senescence markers in oral cancer, oral leukoplakia and control samples in humans. Oncology letters. 2012;3(6):1319-25. Epub 2012/07/12. doi: 10.3892/ol.2012.649. PubMed PMID: 22783442; PubMed Central PMCID: PMCPMC3392562.

31. Pigatti FM, Taveira LA, Soares CT. Immunohistochemical expression of Bcl-2 and Ki-67 in oral lichen planus and leukoplakia with different degrees of dysplasia. Int J Dermatol. 2015;54(2):150-5. Epub 2014/10/01. doi: 10.1111/ijd.12279. PubMed PMID: 25265990.

32. Poomsawat S, Buajeeb W, Khovidhunkit SO, Punyasingh J. Alteration in the expression of cdk4 and cdk6 proteins in oral cancer and premalignant lesions. Journal of oral pathology & medicine : official publication of the International Association of Oral Pathologists and the American Academy of Oral Pathology. 2010;39(10):793-9. Epub 2010/07/14. doi: 10.1111/j.1600-0714.2010.00909.x. PubMed PMID: 20618617.

33. Raghunandan BN, Sanjai K, Kumaraswamy J, Papaiah L, Pandey B, Jyothi BM. Expression of human telomerase reverse transcriptase protein in oral epithelial dysplasia and oral squamous cell carcinoma: An immunohistochemical study. Journal of oral and maxillofacial pathology : JOMFP. 2016;20(1):96-101. doi: 10.4103/0973-029X.180953. PubMed PMID: 27194869; PubMed Central PMCID: PMC4860945.

34. Raju B, Mehrotra R, Oijordsbakken G, Al-Sharabi AK, Vasstrand EN, Ibrahim SO. Expression of p53, cyclin D1 and Ki-67 in pre-malignant and malignant oral lesions: association with clinicopathological parameters. Anticancer research. 2005;25(6C):4699-706. PubMed PMID: 16334163.

35. Ralhan R, Chakravarti N, Kaur J, Sharma C, Kumar A, Mathur M, et al. Clinical significance of altered expression of retinoid receptors in oral precancerous and cancerous lesions: relationship with cell cycle regulators. International journal of cancer. 2006;118(5):1077-89. doi: 10.1002/ijc.21483. PubMed PMID: 16161051.

36. Rich AM, Kerdpon D, Reade PC. p53 expression in oral precancer and cancer. Australian dental journal. 1999;44(2):103-5. PubMed PMID: 10452165.

37. Saito T, Nakajima T, Mogi K. Immunohistochemical analysis of cell cycle-associated proteins p16, pRb, p53, p27 and Ki-67 in oral cancer and precancer with special reference to verrucous carcinomas. Journal of oral pathology & medicine : official publication of the International Association of Oral Pathologists and the American Academy of Oral Pathology. 1999;28(5):226-32. PubMed PMID: 10226946.

38. Viveka TS, Shyamsundar V, Krishnamurthy A, Ramani P, Ramshankar V. p53 Expression Helps Identify High Risk Oral Tongue Pre- malignant Lesions and Correlates with Patterns of Invasive Tumour Front and Tumour Depth in Oral Tongue Squamous Cell Carcinoma Cases. Asian Pacific journal of cancer prevention : APJCP. 2016;17(1):189-95. doi: 10.7314/apjcp.2016.17.1.189. PubMed PMID: 26838208.

39. Varun BR, Ranganathan K, Rao UK, Joshua E. Immunohistochemical detection of p53 and p63 in oral squamous cell carcinoma, oral leukoplakia, and oral submucous fibrosis. Journal of investigative and clinical dentistry. 2014;5(3):214-9. doi: 10.1111/jicd.12038. PubMed PMID: 23776093.

40. Turatti E, da Costa Neves A, de Magalhaes MH, de Sousa SO. Assessment of c-Jun, c-Fos and cyclin D1 in premalignant and malignant oral lesions. Journal of oral science. 2005;47(2):71-6. PubMed PMID: 16050486.

41. Kamat SS, Kumar GS, Koshy AV. Immunohistochemical analysis of syndecan-1 in leukoplakia and oral submucous fibrosis. Dental research journal. 2013;10(3):321-7. PubMed PMID: 24019799; PubMed Central PMCID: PMC3760354.

42. Surendran S, Siddappa G, Mohan A, Hicks W, Jr., Jayaprakash V, Mimikos C, et al. Cancer stem cell and its niche in malignant progression of oral potentially malignant disorders. Oral oncology. 2017;75:140-7. doi: 10.1016/j.oraloncology.2017.11.003. PubMed PMID: 29224811; PubMed Central PMCID: PMC5880641.

43. Sridevi U, Jain A, Nagalaxmi V, Kumar UV, Goyal S. Expression of E-cadherin in normal oral mucosa, in oral precancerous lesions and in oral carcinomas. European journal of dentistry. 2015;9(3):364-72. Epub 2015/10/03. doi: 10.4103/1305-7456.163238. PubMed PMID: 26430364; PubMed Central PMCID: PMCPMC4569987.

44. Schoelch ML, Regezi JA, Dekker NP, Ng IO, McMillan A, Ziober BL, et al. Cell cycle proteins and the development of oral squamous cell carcinoma. Oral oncology. 1999;35(3):333-42. PubMed PMID: 10621856.

45. Sinanoglu A, Soluk-Tekkesin M, Olgac V. Cyclooxygenase-2 and Ki67 Expression in Oral Leukoplakia: a Clinicopathological Study. Journal of oral & maxillofacial research. 2015;6(2):e3. doi: 10.5037/jomr.2015.6203. PubMed PMID: 26229582; PubMed Central PMCID: PMC4516855.

46. Shintani S, Mihara M, Nakahara Y, Kiyota A, Ueyama Y, Matsumura T, et al. Expression of cell cycle control proteins in normal epithelium, premalignant and malignant lesions of oral cavity. Oral oncology. 2002;38(3):235-43. PubMed PMID: 11978545.
