# Supplementary material for: CD44-SNA1 integrated cytopathology for delineation of high grade dysplastic and neoplastic oral lesions
Source: PLoS One. 2023 Sep 25;18(9):e0291972. doi: 10.1371/journal.pone.0291972 (PMC10519609; doi:10.1371/journal.pone.0291972)
Supplement: S2 Table — Marker details, binding property, dilution factor and methodology used for analysis. (DOCX) [file pone.0291972.s023.docx]

| Number | Marker | Binding Property | Dilution | Experiment | Catalogue Number |
| --- | --- | --- | --- | --- | --- |
| 1 | CyclinD1 | Nuclear | Ready to Use | IHC | AN815-5M (Biognex) |
| 2 | P53 | Nuclear | Ready to Use | IHC | IS61630-2 (Dako) |
| 3 | hnRNPK | Nuclear | 1:5000 | IHC | ab23644 (abcam) |
| 4 | PTMA | Nuclear | 1:3500 | IHC | LSB2322  (Life Span Bioscience) |
| 5 | S100A7 | Nuclear/Cytoplasmic | 1:500 | IHC | MAB4475 (R&D) |
| 6 | Podoplanin | Cell Membrane | Ready to Use | IHC | ISO07230-2 (DAKO) |
| 7 | CD44 | Cell Membrane/Nucleus | Ready to Use | IHC, ICC | AM310-5M (Biogenex) |
| 8 | WGA (HRP - Wheat Germ Agglutinin) | Cell Membrane | 1:5000 | Lectin Histochemistry | L3892 (Sigma) |
| 9 | SNA-1 (HRP -Sambucus Nigra Agglutinin) | Cell Membrane | 1:250 | Lectin Histochemistry | H-6802 -1 (Ey Labs) |
| 10 | MAA (HRP -Mackia Amurenesis Agglutinin) | Cell Membrane | 1:100 | Lectin Histochemistry | H-7801-1 (EY Labs) |
| 11 | SNA-1 FITC, TRITC | Cell Membrane | 1:10 | ICC | F-6802-1, F-6802-1 (EY Labs) |
| 12 | MAA-FITC | Cell Membrane | 1:10 | ICC | F7801-1(EY Labs) |
| 13 | CD44-FITC | Cell Membrane | 1:10 | ICC | 103005 (BioLegend) |
| 14 | DAPI | Nucleus | 1:1000 | ICC | H-1200 |
| **S2 Table. Details of the markers selected for IHC and ICC validation.** Marker details, binding property, dilution factor and methodology used for analysis. | | | | | |
