# Supplementary material for: CD44-SNA1 integrated cytopathology for delineation of high grade dysplastic and neoplastic oral lesions
Source: PLoS One. 2023 Sep 25;18(9):e0291972. doi: 10.1371/journal.pone.0291972 (PMC10519609; doi:10.1371/journal.pone.0291972)
Supplement: S3 Table — Sensitivity, Specificity and Receiver Operating Characteristic Curve (ROC-AUC) of the markers in differentiating LRL from OSCC/HGD is shown. Table also depicts IHC scores of different cohorts. (DOCX) [file pone.0291972.s024.docx]

| **Marker** | **Sensitivity** | **Specificity** | **AUC** | **LRL** | **HGD** | **OSCC** |
| --- | --- | --- | --- | --- | --- | --- |
|  |  |  |  | (mean± SE) | (mean± SE) | (mean± SE) |
| CD44 | 80 | 84 | 0.87 | 119.74±10.62 | 232.08± 13.46 | 343.20±20.04 |
| SNA-1 | 75 | 78 | 0.85 | 64.27±10.69 | 164.89± 15.53 | 222.64±13.90 |
| Cyclin D1 | 93 | 54 | 0.79 | 72.93±11.79 | 257.56± 15.45 | 253.94±19.68 |
| s100A7 | 80 | 54 | 0.71 | 53.88±9.32 | 98.1±19.47 | 176.85± 17.45 |
| MAA | 88 | 65 | 0.8 | 123.86±16.80 | 216.02± 13.34 | 252.15±17.36 |
| WGA | 46 | 74 | 0.58 | 360.16±10.78 | 366.33± 11.50 | 313.81±12.79 |
| P53 | 58 | 76 | 0.7 | 72.92±11.79 | 103.26±10.04 | 223.17±26.23 |
| **S3 Table. Immunohistochemical Analysis of Phase II validation.** Sensitivity, Specificity and Receiver Operating Characteristic Curve (ROC-AUC) of the markers in differentiating LRL from OSCC/HGD is shown. Table also depicts IHC scores of different cohorts. | | | | | | |
