# Supplementary material for: CD44-SNA1 integrated cytopathology for delineation of high grade dysplastic and neoplastic oral lesions
Source: PLoS One. 2023 Sep 25;18(9):e0291972. doi: 10.1371/journal.pone.0291972 (PMC10519609; doi:10.1371/journal.pone.0291972)
Supplement: S4 Table — Best combination of markers selected by logistic regression were CD44, Cyclin D1 and SNA-1. These markers were significant and showed a Receiver Operating Characteristic Curve Area under Curve (ROC-AUC) of 0.95 in delineating LRL from HGD/OSCC. (DOCX) [file pone.0291972.s025.docx]

| **Variable** | **Coefficient** | **Std. Error** | **P** |
| --- | --- | --- | --- |
| CD44 | 0.017256 | 0.004246 | <0.0001 |
| Cyclin-D1 | 0.009463 | 0.003022 | 0.0017 |
| SNA-1 | 0.018156 | 0.004263 | <0.0001 |
| Constant | -6.6352 |  |  |
| **Variables not included in the model** | | | |
| MAA |  | | |
| P53 |  |  |  |
| S100A7 |  |  |  |
| WGA |  |  |  |
| **Variable** | **Odds ratio** | **95% CI** | |
| CD44 | 1.0174 | 1.0090 to 1.0259 | |
| Cyclin-D1 | 1.0095 | 1.0035 to 1.0155 | |
| SNA-1 | 1.0183 | 1.0098 to 1.0269 | |
| **ROC analysis** | | | |
| AUC | 0.959 | 0.91-0.99 | |
| **S4 Table. Logistic regression model combining all IHC markers by step-wise method.** Best combination of markers selected by logistic regression were CD44, Cyclin D1 and SNA-1. These markers were significant and showed a Receiver Operating Characteristic Curve Area under Curve (ROC-AUC) of 0.95 in delineating LRL from HGD/OSCC. | | | |
