# Supplementary material for: CD44-SNA1 integrated cytopathology for delineation of high grade dysplastic and neoplastic oral lesions
Source: PLoS One. 2023 Sep 25;18(9):e0291972. doi: 10.1371/journal.pone.0291972 (PMC10519609; doi:10.1371/journal.pone.0291972)
Supplement: S5 Table — Selection of best combination of markers was performed by logistic regression analysis. The best markers selected were SNA-1, CD44 and Cyclin D1 with a test sensitivity and specificity of 84% (AUC = 0.88). (DOCX) [file pone.0291972.s026.docx]

|  | coef | std err | z | P>\|z\| | [0.025 | 0.975] |
| --- | --- | --- | --- | --- | --- | --- |
| **const** | -3.0219 | 0.948 | -3.186 | 0.001 | -4.881 | -1.163 |
| **SNA1MAX** | 6.8554 | 1.969 | 3.482 | 0.000 | 2.997 | 10.714 |
| **CD44N%** | -0.0253 | 0.031 | -0.814 | 0.416 | -0.086 | 0.036 |
| **CD44N+** | 3.6843 | 0.937 | 3.931 | 0.000 | 1.847 | 5.521 |
| **CyD1N+** | -1.9367 | 0.835 | -2.318 | 0.02 | -3.574 | -0.299 |
| **Differentiating HRL Vs LRL** | | | | | | |
|  | Training | Test | AUC |  |  |  |
| Sensitivity | 86.53 (45/52) | 84(21/25) | 0.84 |  |  |  |
| Specificity | 76 (19/25) | 84.61(11/13) |  |  |  |  |
| **S5 Table. Logistic regression model of Phase 1 ICC**. Selection of best combination of markers was performed by logistic regression analysis. The best markers selected were SNA-1, CD44 and Cyclin D1 with a test sensitivity and specificity of 84% (AUC =0.88). | | | | | | |
