# Supplementary material for: CD44-SNA1 integrated cytopathology for delineation of high grade dysplastic and neoplastic oral lesions
Source: PLoS One. 2023 Sep 25;18(9):e0291972. doi: 10.1371/journal.pone.0291972 (PMC10519609; doi:10.1371/journal.pone.0291972)
Supplement: S6 Table — Random Forest model was given sensitivity and specificity of 80% and 85% respectively. The most important features selected were SNA-1 and CD44 in delineating LRL from HGD/OSCC (HRL). (DOCX) [file pone.0291972.s027.docx]

| **Markers** | | **Importance** |
| --- | --- | --- |
| SNA1% positivity | | 0.35 |
| CD44%>4 Intensity | | 0.2 |
| SNA1 Maximum Intensity | | 0.15 |
| CD44 maximum Intensity | | 0.05 |
| SNA-1 Average Intensity | | 0.04 |
| **HRL Vs LRL** | **Training** | **Test** |
| Accuracy | 87% | 82% |
| Sensitivity | 88% (46/52) | 80%(20/25) |
| Specificity | 87% (22/25) | 84.62%(11/13) |
| **S6 Table. Random Forest model for feature selection.** Random Forest model was given sensitivity and specificity of 80% and 85% respectively. The most important features selected were SNA-1 and CD44 in delineating LRL from HGD/OSCC (HRL). | | |
