# Supplementary material for: CD44-SNA1 integrated cytopathology for delineation of high grade dysplastic and neoplastic oral lesions
Source: PLoS One. 2023 Sep 25;18(9):e0291972. doi: 10.1371/journal.pone.0291972 (PMC10519609; doi:10.1371/journal.pone.0291972)
Supplement: S7 Table — Multiple machine learning models were trained and validated using single marker Phase I ICC (SNA-1 +CD44) and multiplex marker Phase II ICC in delineating LRL from HGD/OSCC (HRL). (DOCX) [file pone.0291972.s028.docx]

| Single Marker Validation (Phase I ICC) : Split (70:30); HRL Vs LRL | | | | | | | | | | | | | |
| --- | --- | --- | --- | --- | --- | --- | --- | --- | --- | --- | --- | --- | --- |
| Model | Logistic Regression | | Regularized Log regression (L2) | | Random Forest | | SVM | | KNN | | XgBoost | | |
|  | Train | Test | Train | Test | Train | Test | Train | Test | Train | Test | Train | Test |  |
| Sensitivity | 92.73  (47/55) | 87.5  (21/24) | 83.64 (46/55) | 83.33 (20/24) | 87.27 (48/55) | 83.33 (20/24) | 89.09 (49/55) | 75 (18/24) | 90.9 (45/55) | 83.33 (20/24) | 92.73 (51/55) | 87.5 (21/24) |  |
| Specificity | 73.68 (28/38) | 88.23 (15/17) | 81.58 (31/38) | 94.12 (16/17) | 84.21 (32/38) | 88.24 (15/17) | 84.21 (32/38) | 82.35 (14/17) | 81.58 (31/38) | 82.35 (13/17) | 76.32 (29/38) | 70.58 (12/17) |  |
| Accuracy | 84.95 | 87.8 | 82.8 | 87.8 | 86.02 | 85.37 | 87.1 | 78.04 | 81.72 | 82.9 | 86.02 | 80.49 |  |
| AUC | 0.92 | 0.88 | 0.83 | 0.89 | 0.86 | 0.86 | 0.87 | 0.79 | 0.86 | 0.83 | 0.85 | 0.85 |  |
| Hyperparameter / Features | SNA1maximum Intensity (p=0.002), CD44% >4Intensity (p=0.049), CD44 Nucleus Positive (p=0.000), No Habit (p= 0.06) | | Logistic penality = L2, PCA_component =7, Logistic_c = 0.2 | | Class Weight = balanced, Max_depth =3, Min sample leaf =5, minimum sample spit =6, max features =6, n_estimators = 30, | | class_weight= 'balanced', C=10, gamma=0.01, kernel='rbf' | | N=7 | | n_estimators=20, max_depth=3, learning_rate=0.001, colsample_bytree=0.5, gamma=0.2, min_child_weight=2, objective='binary:logistic', booster='gbtree' | | |
| Multiplex Data (Phase II ICC): Split (60:40); HRL Vs LRL | | | | | | | | | | | | | |
| Sensitivity | 86.11 (31/36) | 79.16 (19/24) | 91.66 (33/36) | 91.67 (22/24) | 83.33 (30/36) | 87.5 (21/24) | 97.22 (35/36) | 91.67 (22/24) | 77.78 (28/36) | 83.33 (20/24) | 86.11 (31/36) | 83.33 (20/24) |  |
| Specificity | 93.48 (43/46) | 84.38 (27/32) | 91.30 (42/46) | 84.37 (27/32) | 89.13 (41/46) | 90.62 (29/32) | 82.61 (38/46) | 75 (24/32) | 91.30 (42/46) | 93.75 (30/32) | 95.66 (44/46) | 90.63 (29/32) |  |
| Accuracy | 90.24 | 82.14 | 91.46 | 87.5 | 86.59 | 89.29 | 89.02 | 82.14 | 85.37 | 89.29 | 91.46 | 97.5 |  |
| AUC | 0.9 | 0.82 | 0.92 | 0.88 | 0.86 | 0.89 | 0.9 | 0.83 | 0.85 | 0.89 | 0.91 | 0.96 |  |
| Hyperparameter / Features | SNA1 Average (p=0.004), SNA1 max (0.004), SNA1 average*CD4average (p=0.002), No Habit(p=0.000) | | Class Weight = balanced, Logistic penality = L2, PCA_component =11, Logistic_c = 7 | | Class Weight = balanced, Max_depth =4, Min sample leaf =10, minimum sample spit =6, max features =15, n_estimatore = 50, | | class_weight= 'balanced', C=10, gamma=0.01, kernel='rbf' | | N=9 | | n_estimators=55, max_depth=3, learning_rate=0.04, colsample_bytree=0.5, gamma=0.2, min_child_weight=3, objective='binary:logistic', booster='gbtree' | | |
| S7 Table. Training and test results of machine learning models for ICC Validation. Multiple machine learning models were trained and validated using single marker Phase I ICC (SNA-1 +CD44) and multiplex marker Phase II ICC in delineating LRL from HGD/OSCC (HRL). | | | | | | | | | | | | | |
