# Supplementary material for: CD44-SNA1 integrated cytopathology for delineation of high grade dysplastic and neoplastic oral lesions
Source: PLoS One. 2023 Sep 25;18(9):e0291972. doi: 10.1371/journal.pone.0291972 (PMC10519609; doi:10.1371/journal.pone.0291972)
Supplement: S8 Table — Molecular multiplex cytology was compared with conventional Haematoxylin and Eosin slides interpreted by pathologist. (DOCX) [file pone.0291972.s029.docx]

|  | *Molecular Cytology* | | *Conventional Cytology* | |
| --- | --- | --- | --- | --- |
|  | ***OSCC Vs LRL*** | ***HGD Vs LRL*** | ***OSCC Vs LRL*** | ***HGD Vs LRL*** |
| **Sensitivity** | 91.43 (32/35) | 92 (23/25) | 80(20/25) | 29.41(5/17) |
| **Specificity** | 92.31(72/78) | 80.77(63/78) | 76.25(61/80) | 76.25(61/80) |
| **Accuracy** | 92.04 | 83.5 | 77.14 | 68.04 |
| **S8 Table. Phase II ICC Vs Conventional Cytology.** Molecular multiplex cytology was compared with conventional Haematoxylin and Eosin slides interpreted by pathologist. | | | | |
