# Supplementary material for: CD44-SNA1 integrated cytopathology for delineation of high grade dysplastic and neoplastic oral lesions
Source: PLoS One. 2023 Sep 25;18(9):e0291972. doi: 10.1371/journal.pone.0291972 (PMC10519609; doi:10.1371/journal.pone.0291972)
Supplement: S9 Table — Sensitivity and specificity of neural network for classification of cancer cells from normal cells. (DOCX) [file pone.0291972.s030.docx]

| Model | Training/test | Sensitivity | Specificity | Accuracy |
| --- | --- | --- | --- | --- |
| Inception-V3 | Training | 90.27 | 99.80 | 96.41 |
|  | Test | 75.47 | 100 | 93.36 |
| Cancer-Net | Training | 90.81 | 96.22 | 92.79 |
|  | Test | 88.67 | 97.90 | 95.41 |
| **S9 Table. Validation and Testing of Neural network for atypical cell classification**. Sensitivity and specificity of neural network for classification of cancer cells from normal cells. | | | | |
