# Supplementary material for: CD44-SNA1 integrated cytopathology for delineation of high grade dysplastic and neoplastic oral lesions
Source: PLoS One. 2023 Sep 25;18(9):e0291972. doi: 10.1371/journal.pone.0291972 (PMC10519609; doi:10.1371/journal.pone.0291972)
Supplement: S10 Table — The normal distribution and variance among cohorts were tested using the Normality test and ANOVA respectively. The colored rows showed significant features (p<0.05). Lectin intensity = SNA-1 Intensity; N_C ratio = Nuclear cytoplasmic ratio, prob_cancer = CancerNet prediction, Maj_axis = Major axis ratio, Min_axis = Minor axis ratio. (DOCX) [file pone.0291972.s031.docx]

| **Feature** | **Normality (p values)** | **Kruskal Wallis (p value)** |
| --- | --- | --- |
| Prob average of cancer cells (Cancer Net prediction) | 7.928103e-09 | 4.533553e-16 |
| Ratio of cancer cells | 1.083874e-08 | 2.996957e-10 |
| lectin_intensity_mean (SNA-1) | 2.905723e-10 | 9.682751e-04 |
| DAPI_int_mean | 1.104959e-01 | 5.798418e-03 |
| N_C_ratio_mean | 2.075023e-08 | 2.430528e-05 |
| diameter_ratio_mean | 2.518709e-01 | 7.688180e-05 |
| maj_axis_ratio_mean | 9.881509e-01 | 7.319062e-05 |
| min_axis_ratio_mean | 3.002991e-01 | 5.175003e-05 |
| perimeter_ratio_mean | 3.024525e-08 | 2.120859e-04 |
| solidity_cell_mean | 3.860311e-45 | 1.351070e-02 |
| solidity_nucleus_mean | 1.087845e-06 | 3.376104e-02 |
| orientation_cell_mean | 3.317483e-02 | 9.075433e-01 |
| orientation_nucleus_mean | 2.886982e-01 | 7.206306e-01 |
| eccentricity_cell_mean | 9.524385e-01 | 1.278467e-03 |
| eccentricity_nucleus_mean | 3.228254e-01 | 6.206579e-06 |
| convexarea_cell_mean | 2.957515e-01 | 7.242315e-05 |
| convexarea_nucleus_mean | 4.427612e-08 | 7.356807e-06 |
| Prob_cancer_mean | 1.192017e-08 | 2.765573e-12 |
| lectin_intensity_max (SNA-1) | 8.800300e-03 | 1.150672e-05 |
| DAPI_int_max | 1.435599e-24 | 1.714040e-01 |
| N_C_ratio_max | 8.101527e-43 | 2.113654e-04 |
| diameter_ratio_max | 4.677356e-16 | 4.932378e-01 |
| maj_axis_ratio_max | 1.150070e-22 | 6.014400e-01 |
| min_axis_ratio_max | 3.489533e-18 | 4.373673e-01 |
| perimeter_ratio_max | 8.645963e-18 | 7.504691e-01 |
| solidity_cell_max | 5.761558e-03 | 4.797374e-02 |
| solidity_nucleus_max | 9.564120e-03 | 5.005092e-01 |
| orientation_cell_max | 2.161366e-18 | 1.141723e-01 |
| orientation_nucleus_max | 6.169836e-18 | 8.634207e-01 |
| eccentricity_cell_max | 2.178275e-15 | 2.930633e-01 |
| eccentricity_nucleus_max | 1.684031e-14 | 7.120016e-02 |
| convexarea_cell_max | 1.768217e-01 | 2.163972e-06 |
| convexarea_nucleus_max | 1.248986e-02 | 1.601433e-03 |
| Prob_cancer_max | 3.706278e-14 | 4.340262e-14 |
| lectin_intensity_ std deviation (SNA-1) | 1.802155e-03 | 9.947832e-05 |
| DAPI_int_ std deviation | 3.689325e-01 | 6.356504e-02 |
| N_C_ratio_ std deviation | 1.499910e-40 | 5.450725e-05 |
| diameter_ratio_ std deviation | 5.398588e-09 | 6.334123e-01 |
| maj_axis_ratio_ std deviation | 4.004834e-10 | 6.952509e-02 |
| min_axis_ratio_ std deviation | 9.723782e-09 | 5.736468e-01 |
| perimeter_ratio_ std deviation | 5.608427e-16 | 5.104016e-01 |
| solidity_cell_ std deviation | 1.344118e-52 | 1.821618e-02 |
| solidity_nucleus_std deviation | 8.298905e-03 | 5.313098e-03 |
| orientation_cell_std | 1.212774e-01 | 1.209370e-01 |
| orientation_nucleus_std | 1.689251e-03 | 2.643775e-01 |
| eccentricity_cell_std | 9.786186e-02 | 2.151901e-01 |
| eccentricity_nucleus_std | 4.122973e-03 | 1.464969e-01 |
| convexarea_cell_std | 3.937257e-01 | 9.322854e-03 |
| convexarea_nucleus_std | 6.865593e-05 | 6.581274e-04 |
| Prob_cancer_std | 7.668758e-05 | 1.763871e-14 |
| **S10 Table. Normality and ANOVA test of patients wise features.** The normal distribution and variance among cohorts were tested using the Normality test and ANOVA respectively. The colored rows showed significant features. Lectin intensity=SNA-1 Intensity; N_C ratio= Nuclear cytoplasmic ratio, prob_cancer= CancerNet prediction, Maj_axis= Major axis ratio, Min_axis= Minor axis. | | |
