# Supplementary material for: CD44-SNA1 integrated cytopathology for delineation of high grade dysplastic and neoplastic oral lesions
Source: PLoS One. 2023 Sep 25;18(9):e0291972. doi: 10.1371/journal.pone.0291972 (PMC10519609; doi:10.1371/journal.pone.0291972)
Supplement: S11 Table — Final model developed by tuning the VIF and significance. VIF = Variation Inflation Factor (automated Phase I ICC SNA-1 data). (DOCX) [file pone.0291972.s032.docx]

|  | coef | std err | z | P>\|z\| | [0.025 | 0.975] | VIF |
| --- | --- | --- | --- | --- | --- | --- | --- |
| constant | 0.0626 | 0.343 | 0.182 | 0.855 | -0.610 | 0.735 |  |
| Probability_Cancer_avg | 2.4937 | 0.747 | 3.337 | 0.001 | 1.029 | 3.959 | 1.45 |
| Nuclear Cytoplasmic area ratio_mean | -1.6888 | 0.702 | -2.406 | 0.016 | -3.064 | -0.313 | 3.22 |
| min_axis_ratio_mean | -1.9851 | 0.750 | -2.648 | 0.008 | -3.454 | -0.516 | 3.26 |
| convexarea_cell_maximum | 0.5480 | 0.331 | 1.657 | 0.098 | -0.100 | 1.196 | 1.12 |
| **S11 Table. Logistic Regression model.** Final model developed by tuning the VIF and significance. VIF=Variation Inflation Factor. | | | | | | | |
