# Supplementary material for: CD44-SNA1 integrated cytopathology for delineation of high grade dysplastic and neoplastic oral lesions
Source: PLoS One. 2023 Sep 25;18(9):e0291972. doi: 10.1371/journal.pone.0291972 (PMC10519609; doi:10.1371/journal.pone.0291972)
Supplement: S12 Table — Sensitivity, specificity of machine learning models for classifying oral cancer and HGD from LRL. TP = true positive; TN = True Negative; FP = False Positive; FN = False Negative (automated Phase I ICC SNA-1 data). (DOCX) [file pone.0291972.s033.docx]

|  | | | | |
| --- | --- | --- | --- | --- |
| **Method** | **Training (70%; n= 93)** | | **Test (30%; n=40)** | |
|  | Sensitivity % (TP / TP+FN) | Specificity % (TN/ TN+FP) | Sensitivity % (TP / TP+FN) | Specificity % (TN/ TN+FP) |
| Logistic Regression  (AUC= 0.92) | 89 (49/55) | 82 (31/38) | 87 (20/23) | 82 (14/17) |
| PCA and Regularized Logistic regression (AUC =0.87) | 87 (48/55) | 87 (33/38) | 83(19/23) | 88(15/17) |
| Random Forest  (AUC =0.95) | 95(52/55) | 95(36/38) | 83(19/23) | 88(15/17) |
| **S12 Table. Comparison of models.** Sensitivity, specificity of machine learning models for classifying oral cancer and HGD from LRL. TP=true positive; TN=True Negative; FP=False Positive; FN=False Negative. | | | | |
