# Supplementary material for: CD44-SNA1 integrated cytopathology for delineation of high grade dysplastic and neoplastic oral lesions
Source: PLoS One. 2023 Sep 25;18(9):e0291972. doi: 10.1371/journal.pone.0291972 (PMC10519609; doi:10.1371/journal.pone.0291972)
Supplement: S1 Appendix — (DOCX) [file pone.0291972.s034.docx]

1. **IHC Protocol**

5μM sections were made of FFPE tissue blocks on 2% tri-ethoxy silane coated slides. Before starting the IHC, the slides were incubated for 12 hours at 58-60^0^C. The IHC protocol is described as follows. The slides were kept in Xylene two times, 5 mins each. After wiping, the slides were kept at 100% ethanol and changed to 70% and 50% ethanol for 3 min each. Slides were kept under running tap water for 5 mins, followed by distilled water for 2 mins. After wiping, the slides were kept in 3% H2O2 (6ml H2O2+ 194ml methanol) for 30 mins. The slides were washed with water for 5 mins and kept for 2 mins in distilled water. After wiping, slides were kept in TRIS-EDTA (6gm TRIS+740 mg EDTA dissolved in distilled water, PH=9) and heated in a microwave oven for 17 minutes total: 5 mins in 100^0^C, 5 mins in 80^0^C, 5 mins in 60^0^C, 2 mins in 30^0^C. Slides were cooled at room temperature for 1 hour and washed under tap water for 5 mins and in distilled water for 2 mins. Slides were kept in TBST (40gm NaCl, 3.025gm TRIS, 500ul Tween 20, 22ml 1N HCl, PH=7.5- 8) for two times (2 mins) each. After wiping, the tissue sections on the slides were marked with a PAP pen, and BSA (3% in PBS) was added to the sections and kept for 15-20 mins. Slides were incubated with primary antibody (1hr) in a humid chamber. After two TBST washes, each for 2 mins, a secondary antibody was added to the tissue sections and kept for 30 mins. Slides were again washed in TBST two times, 2 mins each. After wiping, DAB (1ml substrate buffer + 15ul chromogen) was added to each section and was kept for one minute. Washing was done in TBST for 3 mins. Hematoxylin was added to each slide and kept for one minute. Slides were washed in tap water for 5mins. After completing air drying, slides were mounted with DPX and coverslip and kept for drying.

Lectin histology: HRP-conjugated Lectin was used to evaluate the expression in the same patient cohorts by lectin histochemistry. The slides were processed as detailed in the IHC protocol, incubated with HRP conjugated WGA (30 min), and subsequent hematoxylin staining. The scoring protocol will be followed as mentioned before. A known positive control and negative control were stained for each antibody to confirm the presence of appropriate immunostaining activity. Staining in the nucleus, cytoplasm, and/or cell membranes indicates positive expression.

The sections were visualized at 10x and 40x magnification (Nikon Eclipse E200), and the intensity (2+, 4+, or 6+), the pattern of staining, and percentage positivity (0-100%) were assessed (5-10 images/slide; 40x) (Nikon DSFi2 and NIS elements D4 20.0). The total score is obtained by multiplying the percentage of cells stained with the intensity.

1. **Immunocytology (ICC)- Single marker staining**

**ICC Materials** SurePath was obtained from BD Biosciences, a Secondary Detection system (# K5007) from Dako Real Envision). Primary antibodies were procured. All common chemicals, such as phosphate buffer saline (PBS), were from Sigma Aldrich, while DAPI (1:1000) was from Vector Laboratories (Catalog #: H1200); slides, coverslips, and other glassware were obtained from Biogen laboratories.

The medium used for cell culture was Dulbecco’s Modified Essential Medium (DMEM, Cat # 11965092, GIBCOTM, Massachusetts, USA) and Dulbecco’s Modified Essential Medium: Nutrient Mix F12 (DMEM: F12, Cat # 11320033 , GIBCOTM, Massachusetts, USA) along with fetal bovine serum (FBS, Cat # RM10434, HiMedia Laboratories Pvt. Ltd., Mumbai, India). The growth supplements used were Hydrocortisone (Cat # H0888, Sigma Aldrich, St. Louis, MO, USA); Non-Essential Amino Acids (NEAA, Cat # 11140050, GIBCOTM, Massachusetts, USA); Insulin (Cat # I9278-5ml, Sigma Aldrich, St. Louis, MO, USA)

**Cytology Sample collection *Cell lines:*** The chosen markers were first tested in OSCC cell lines produced from various phases. Cal-27 (derived from a moderately differentiated squamous cell carcinoma of the tongue; obtained as a gift from the Institute of Bioinformatics in Bangalore) was cultured in DMEM (FBS 10% + antibiotics 1%), while DOK (a dysplastic cell line obtained from RPCI in New York, USA) was cultured in DMEM (FBS 10% +5ugm/ml hydrocortisone). For cytology investigations, the cell lines were expanded using conventional methods, passaged, and cultured to 70-80% confluence. The expression of OSCC cell lines/DOK will be compared to the expression of normal epithelial cells.

***Oral Brush Biopsy:*** After obtaining written informed consent, oral epithelial cells were obtained from oral lesions and contralateral normal areas prior to the biopsy. The participants who were not candidates for biopsy (benign lesions, normal mucosa) were monitored as clinically necessary to confirm the clinical diagnosis. Cells from the buccal mucosa, tongue, and gingiva of healthy persons with no habits or oral diseases were used to create the nomogram. For ulcerated and proliferative lesions, the cytology brush was rotated ten times, or >25 times for non-ulcerated lesions, until blood tinge was noticed. Cells were harvested using a cervical cytology brush or Rover Orocellex brush (Rovers Medical Devices B.V., Netherlands). The latter was used if the mouth opening of the patients was less than two finger width or lesion was in poorly accessible locations such as retro molar trigone floor of mouth or palate. The tip of the brush was triturated into a cell preservative solution (BD SurePath, BD Biosciences, USA) by rotating 10 -15 times in one direction and stored.

**Cytology staining Protocol** Immunocytology was performed by three different methods: HRP-DAB, immunofluorescence-based method, and multiplex method. Immunocytology (HRP-DAB methods) was carried out using standard methods. The cells were collected in SurePath and the cytology slides were prepared using Cytospin 4 (Thermo-Scientific) by centrifugation at 500rpm for 5 minutes. The primary antibody was then applied to the prepared slides in accordance with precise dilutions for an hour, and staining was discovered utilizing the secondary detection method (Dako Real Envision, K5007). A known positive and negative control was stained for each antibody to confirm the presence of appropriate immunostaining activity. Staining in the nucleus, cytoplasm, and/or cell membranes indicates positive expression. The slides were visualized at 10x, 20x, and 40x magnification (Nikon Eclipse E200), and the intensity, the pattern of staining, and percentage positivity (0-100%) assessed (15 images/slide; 20x) (Nikon DSFi2 and NIS elements D4 20.0).

***Immunofluorescence method*** Oral epithelial cells were extracted from the participants and kept in SurePath solution (BD SurePath), after which slides were prepared according to usual procedures and stained with Lectin for 15 minutes. The Cytospin (Thermo-Scientific) was used to create the cytology slides, which were spun at 500rpm for 5 minutes and counterstained with the nuclear stain DAPI. Images were captured with a fluorescent microscope (Zeiss C, Axiocam, and Zen lite 2012), the intensity of uptake was quantified (50-70 cells, Image-J), and the differences in aberrant sialylation were compared across the different assays/samples. Normal, Benign, OPMD, and ML intensity levels were compared.

**Cytology Image analysis using ImageJ**

Image analysis using image J: The cytology images were taken in 200x, and individual cells were selected from less clumped areas. The intensity of marker staining was measured using ImageJ. The cells of interest were selected using drawing/selection tools (i.e., polygon or freeform), and from the analyze menu, "set measurements" were selected. The intensity is measured using the "Measure" tab from the analyze menu, and this step is repeated for the other cells in the field of view. The results were extracted, and the mean and maximum intensity scores were measured from each cell/patient. The steps are given in detail below:

i. Select the cell of interest using any of the drawing/selection tools (i.e., polygon or freeform) and press to select multiple Region of interest

ii. From the Analyze menu, select "set measurements".

iii. Now select "Measure" from the analyze menu. Now will see a popup box with a stack of values for that first cell and the cells that are selected.

iv. Repeat this step for the other cells in the field of view that you want to measure.

v. Once you have finished, select all the data in the Results window and copy and paste them into a new excel worksheet (or similar program).

vi. The mean and maximum intensity scores will be measured in each image and will consider for statistical analysis.

As the image analysis pipeline is developed after the visualization of ICC data, detailed deep learning and machine learning methods are explained in detail within each chapter.


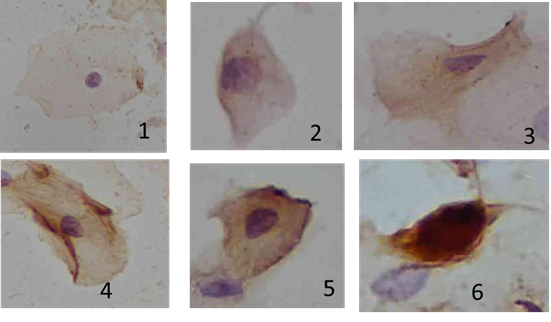


**Figure SF-Fig1. Scoring intensity of HRP-DAB-stained cells:** Microscopic image depicts ICC intensity score and related images of single cell.

**Staining with Cyclin D1 and CD44** The slides were visualized in 200x magnification, and the intensity (0-6; ***Figure SF-Fig1***), the pattern of staining, and percentage positivity were assessed (10-15) images per slide (number of cells =70-100). **Immunofluorescence (SNA-1, MAA)** images: The methodology of the staining procedure and Image J- Image Analysis Pipeline (https://imagej.nih.gov/ij/). The cells of interest were selected using drawing/selection tools (i.e., polygon or freeform) and from the analyze menu "set measurements" selected. The intensity is measured using the "Measure" tab from the analyze menu, and this step is repeated for the other cells in the field of view. The results were extracted, and the mean and maximum intensity scores measured from each cell/patient (Figure SF-Fig 2)

**Figure SF2 Image analysis Pipeline using Image J:** Workflow depicting manual method of image analysis using image J. Selecting single cells and measuring the mean density of image


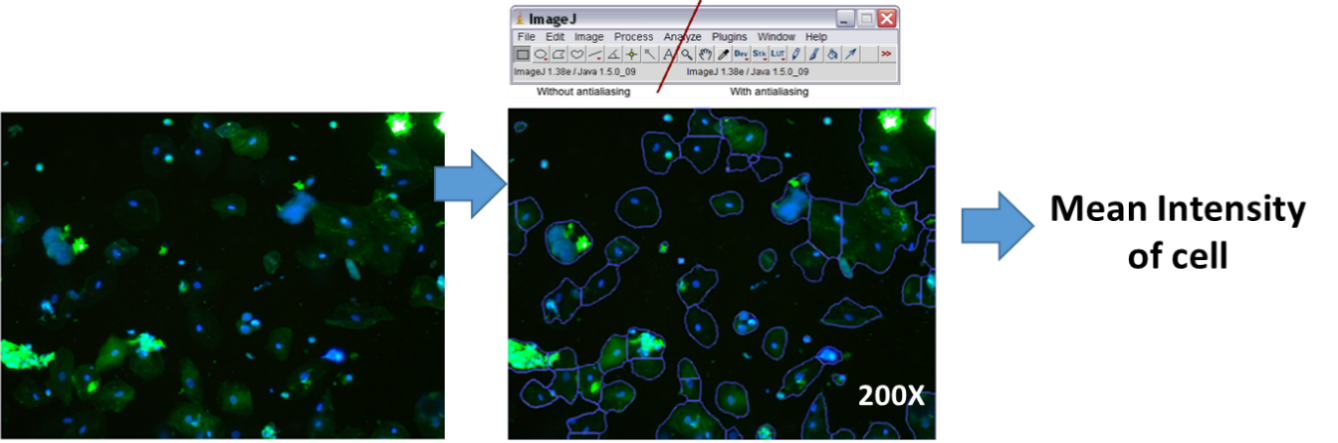


1. **Multiplex cytology standardization**

The selected markers, fluorescent conjugated CD44-FITC and SNA-1-TRITC were carried forward for standardization of the multiplexed assay. In order to establish the multiplex assay in terms of the fixation protocol, three different staining protocols with OSCC patients (n=3) in duplicates were carried out. The cytology protocol and analysis (Image J) were as described previously. Briefly, the cells were stained with the two markers (CD44/SNA-1 were stained for 30 minutes) sequentially with washes (twice with PBS) in between every step. The modifications in the fixation step were as follows.

- Experiment (ex) 1: stained initially with SNA-1, followed by fixation and staining with CD44/DAPI
- Experiment (ex) 2: the cells were fixed initially and then stained sequentially with SNA-1 and CD44
- Experiment (ex) 3: the cells were sequentially stained with SNA-1, CD44 and DAPI without fixing.

A comparison of the three experiments showed that fixation decreases the mean intensity of SNA-1 (***Figure SF-Fig 3***, p<0.005) with no significant difference observed in its absence (ex1 Vs. ex3, p=0.19). CD44 expression *(****Figure SF-Fig4****)*, showed that fixation is essential for optimal mean intensity (ex2=ex3); however, CD44 maximum intensity is not affected (p<0.05) (***Figure SF-Fig4***). Considering these results, we proceeded with a next step validation with patient samples of different cohorts using both the protocols of experiments 3 and 1.

Experiment 3: Validation in patient samples was carried out in benign (n=4), HGD (n=3), and OSCC (n=7) stained with SNA-1-TRITC (30 min) and CD44-FITC (30 min), sequentially (no fixation involved) and the intensity profile was evaluated by image J (***Figure SF-Fig 5 A, B***). However, the results showed no significant difference between the cohorts in CD44 expression (P>0.05), while SNA-1 could differentiate between the cohorts (P<0.05). Experiment 1**:** A sequential staining carried out with SNA-1 and CD44 in the same patient cohort, with a fixation step prior to CD44 staining, indicated that the intensity of CD44 correlated with the severity of the disease (***Figure SF-Fig 5 C, D***). Accordingly, experiment 1 was selected for further experiments.


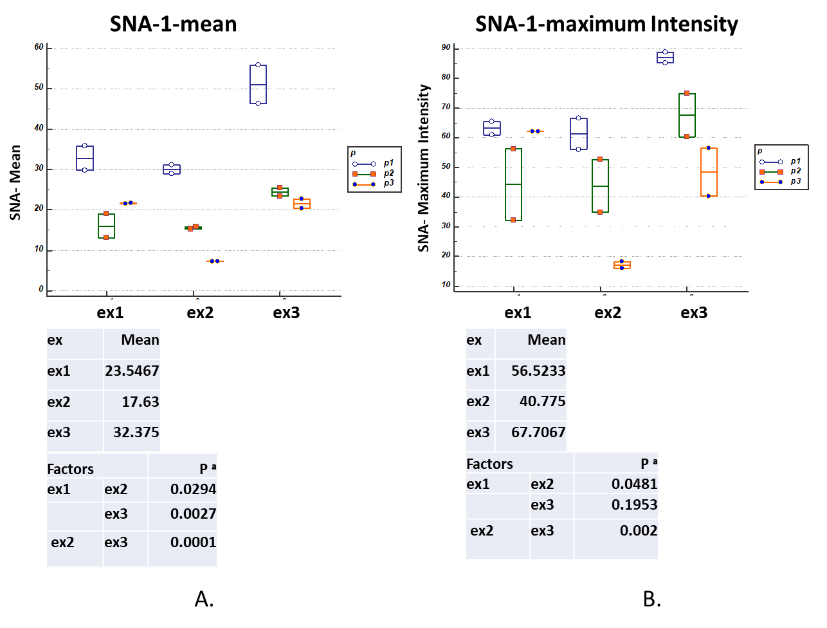


**Figure SF-Fig 3 Depicts the SNA-1 mean intensity and max intensity:** The experiment was carried out in three ways; fixing prior to only CD44 staining (Ex1), prior to staining of both markers (Ex2) and no fixing (Ex3). Fixing before the SNA-1(A) staining decreases the intensity (A) of SNA-1 (ex2) significantly (table, p<0.005). The maximum intensity (B) shows no significant difference between without fixing (ex3 and 1).


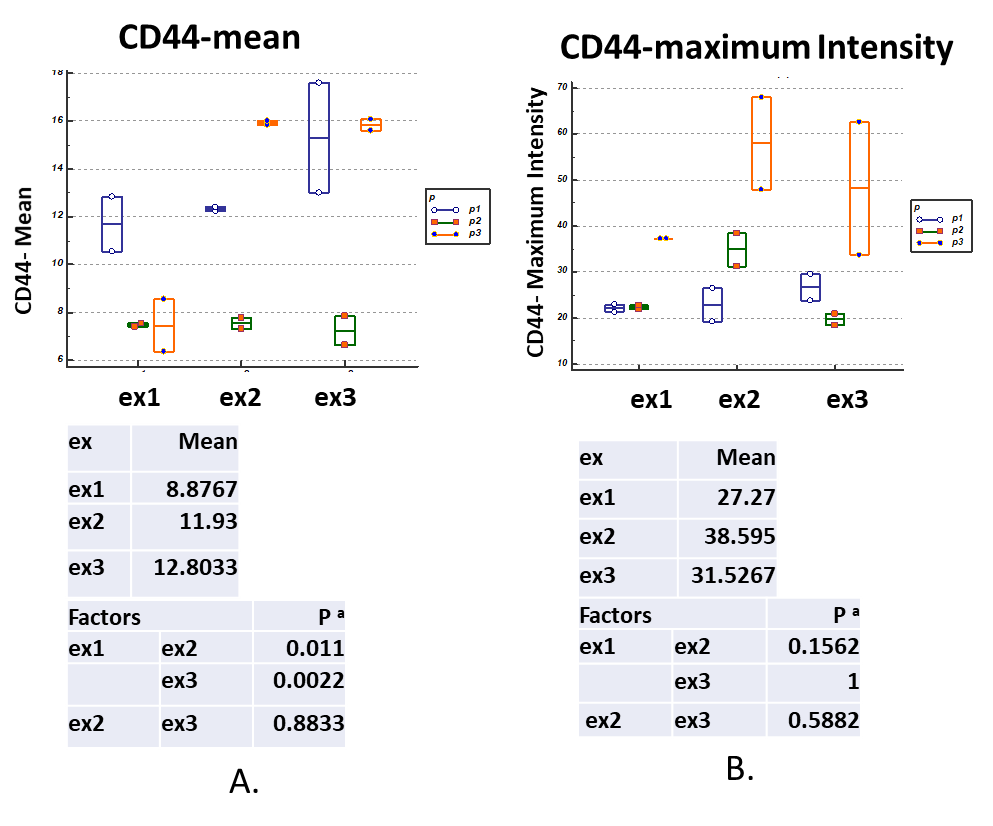


**Figure SF-Fig 4 Depicts the CD44 mean intensity and max intensity:** CD44 expression shows there is only a significant effect if fixing is done before CD44 staining (ex2=ex3). CD44 maximum intensity is not affected by fixing (p<0.05).


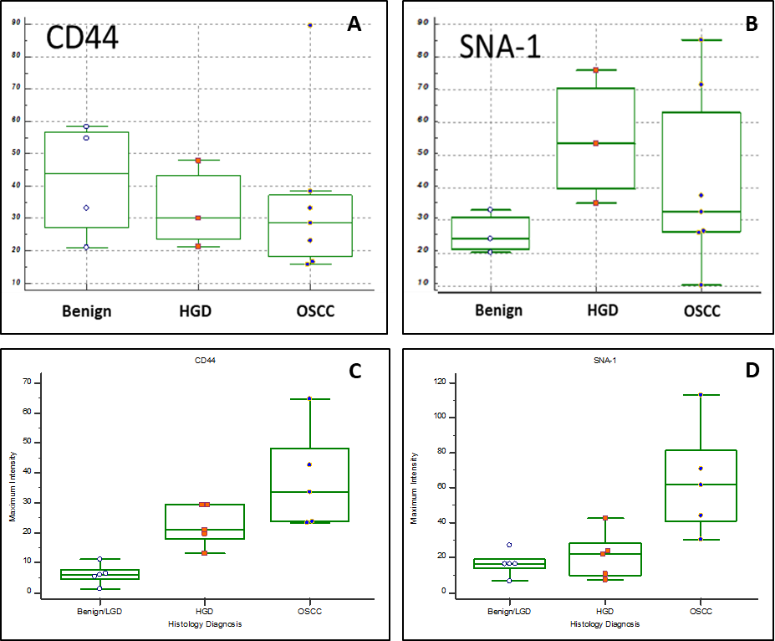


**Figure SF-Fig 5**: **Intensity profile of multiplex pilot study:** representing the maximum intensity of each patient of benign, HGD, and OSCC. CD44 (p>0.05) shows there is no significant difference between cohorts in CD44 (A), and SNA-1 (B, C, D) shows the significant difference between cohorts (p<0.05).
